# SorLA regulates B cell receptor uptake, trafficking, and B cell–mediated immune responses *in vivo*

**DOI:** 10.1084/jem.20252357

**Published:** 2026-08-04

**Authors:** Melibea Berzosa, Adam N. McShane, Anna E. Kliszczak, Aure Aflalo, Pratiti Nanda, Yixuan Shen, Manon H. Williams, Fiona Hills, Chloe M. Shepherd, Kevin O. Saunders, S. Munir Alam, Nicola Ternette, Olav Michael Andersen, Persephone Borrow, Dessislava Malinova

**Affiliations:** 1 Wellcome-Wolfson Institute for Experimental Medicine, Queen’s University Belfast, Belfast, UK; 2 Nuffield Department of Clinical Medicine, Centre for Immuno-Oncology, University of Oxford, Oxford, UK; 3 School of Life Sciences, University of Dundee, Dundee, Scotland, UK; 4 Duke Human Vaccine Institute, Duke University School of Medicine, Duke University, Durham, NC, USA; 5 Department of Surgery, Duke University School of Medicine, Duke University, Durham, NC, USA; 6 Department of Biomedicine, Aarhus University, Aarhus, Denmark

## Abstract

A high-affinity antibody response to both infection and vaccination critically relies on the ability of B cells to capture and process antigen for presentation to CD4^+^ T cells. The cellular processes from antigen recognition to full B cell activation require a finely orchestrated series of events, involving signalling and intracellular trafficking mechanisms. Here, we describe a novel regulator of B cell receptor (BCR) endocytosis and intracellular trafficking. Multidomain trafficking protein sorting-related receptor with A-type repeats (SorLA) associates with the BCR and regulates uptake of both soluble and substrate-bound antigens. SorLA deletion results in altered BCR–antigen intracellular trafficking to degradative compartments, modulating eventual antigen presentation. Crucially, this change in antigen trafficking results in a significant reduction in plasma cells and humoral responses *in vivo*. Given the critical importance of antigen presentation in immunity, as well as autoimmune disease and malignancy, these results identify a new cellular pathway in B cell biology with potential implications for immune regulation.

## Teaser

Berzosa et al. identify SorLA as a key regulator of B cell receptor trafficking, which binds immunoglobulin and controls endosomal sorting. Loss of SorLA impairs antigen degradation and reduces antigen-specific responses *in vivo*, revealing how intracellular trafficking pathways can fine-tune humoral immunity.

## Graphical Abstract

**Figure:**
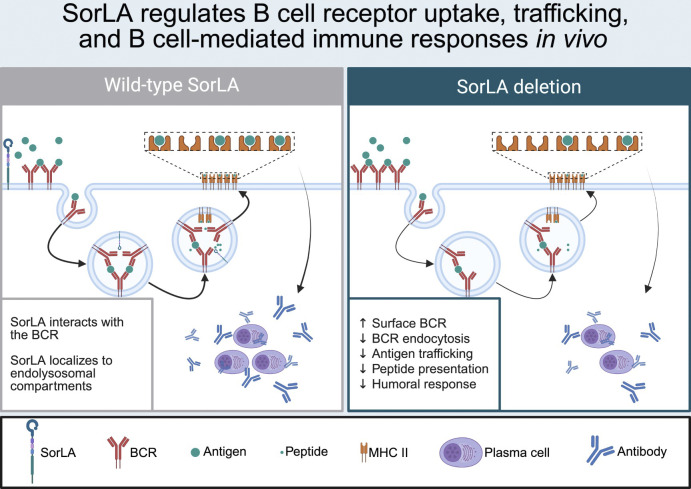

## Introduction

Humoral immune responses are initiated upon antigen binding to a cognate B cell receptor (BCR) at the cell surface, resulting in BCR–antigen endocytosis, trafficking into lysosomal antigen-processing compartments, and loading of the antigenic peptides on MHC class II molecules (MHC II) for their delivery to the cell surface. Display of peptide-loaded MHC II on the B cell surface allows B cells to elicit help from cognate CD4^+^ T cells. This, together with antigen-induced BCR signalling, stimulates metabolic and transcriptional B cell activation, resulting in the formation of germinal centers (GCs) and production of high-affinity antibodies for antigen clearance, as well as memory responses for protection against future antigenic challenge (Kwak et al., 2019; Victora and Nussenzweig, 2022).

In contrast, dysregulated antigen internalization and presentation can lead to B cell immunodeficiency, affecting both susceptibility to microbial infection and vaccination efficacy (Diks et al., 2021). Since BCR–antigen internalization determines the repertoire of peptide–MHC II molecules on the B cell surface, it can affect the quality and quantity of T cell help received and the overall outcome of humoral responses. Consequently, B cell responses are sensitive to mutations or inhibitors impairing the endolysosomal system (Chatterjee et al., 2012; Chaturvedi et al., 2011; Onabajo et al., 2008; Veselits et al., 2014).

The binding between BCR and cognate antigen triggers both downstream signalling cascades, and endocytosis and trafficking of the BCR–antigen complex (Kwak et al., 2019). Early signalling requires phosphorylation of the Igα/Igβ immunoreceptor tyrosine-based activation motifs through recruitment and activation of Src family kinases (Kurosaki, 1999). Concurrently, the BCR–antigen complex can be internalized by several mechanisms: clathrin-mediated endocytosis, fast endophilin-mediated endocytosis, caveolin-dependent endocytosis, and phagocytosis (McShane and Malinova, 2022). The internalized BCR–antigen can be trafficked from the early endosome to endosomal carriers to be recycled back to the plasma membrane or delivered to late endosomes and lysosome-associated membrane protein (LAMP1)^+^ lysosomes for proteolytic processing and loading on MHC II molecules for peptide–MHC II presentation (Barroso et al., 2015; Grant and Donaldson, 2009; Hernández-Pérez et al., 2020; Huotari and Helenius, 2011).

The final destination of internalized cargo depends on posttranslational modifications and interactions with distinct intracellular trafficking adaptors (Drake, 2018; Drake et al., 2006; Li et al., 2020; Olabisi et al., 2006; Veselits et al., 2014; Zhang et al., 2007). However, the molecular composition of BCR–antigen endocytic and intracellular trafficking machinery, and their coordination with B cell signalling dynamics, remain poorly understood. In support of this, genome-wide screening recognized the extent of this coordination, identifying a vast network of intracellular trafficking regulators whose disruption critically affected antigen accumulation in B cells (Malinova et al., 2021).

Here we characterize the role of type-1 transmembrane sorting-related receptor with A-type repeats (SorLA) as a novel regulator of BCR–antigen trafficking in B cells. SorLA, encoded by *SORL1*, is a multifunctional intracellular sorting protein consisting of a large and complex ectodomain that contains a vacuolar protein sorting 10 protein domain, multiple complement-type repeat domains, EGF/YWTD-domains, 3Fn-domains, together with a transmembrane domain, and a short cytoplasmic tail (Schmidt et al., 2017). These domains are essential for SorLA’s sorting functions to direct protein cargos to their correct cellular location. Regulated intracellular trafficking is vital to cell function, and SorLA dysfunction in other cells has been described as the underlying cause of common human diseases by mislocalization of cargos (Schmidt et al., 2017; Talbot et al., 2019). In the genome-wide screen, *SORL1* deletion reduced surrogate antigen uptake in Ramos-Cas9 B cells (Malinova et al., 2021).

In this study, we establish the role of SorLA in B cell antigen uptake, intracellular trafficking of BCR, and eventual antigen presentation by assessing SorLA interactions, antigen degradation dynamics, and B cell function using *in vitro* and *in vivo* models.

## Results

### SorLA localizes to endolysosomal compartments in Ramos B cells

**Figure 1.**
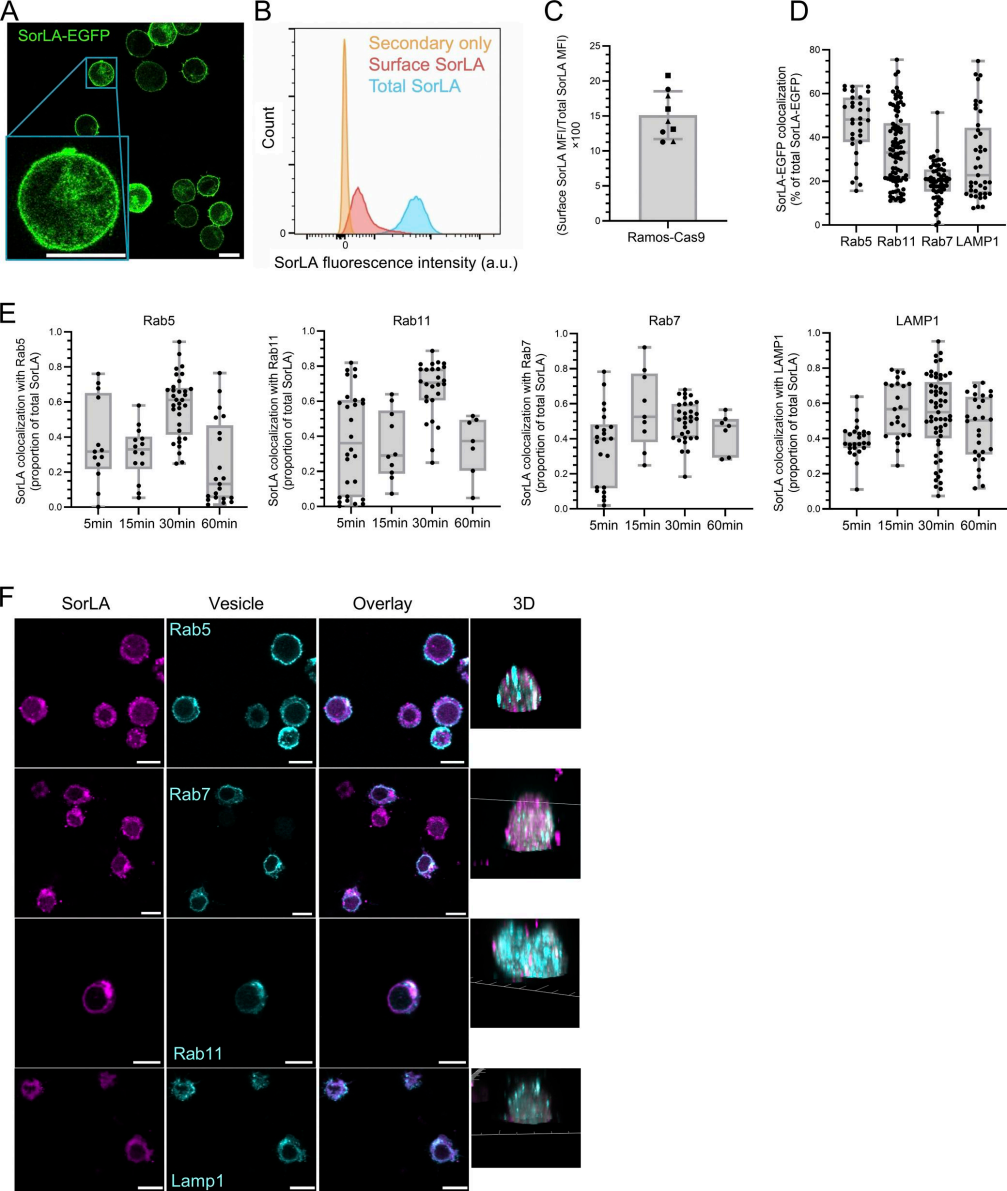
**SorLA localizes to endolysosomal compartments in Ramos B cells. (A)** Confocal micrograph showing surface and intracellular SorLA-EGFP localization in Ramos cells. 100× magnification; inset (blue box) shows an enlarged view of an individual cell. Scale bars = 10 µm. **(B)** Representative flow cytometry histograms for control secondary antibody only stain (orange), unpermeabilized cell surface SorLA stain (red), and 0.01% Triton X-100 permeabilized total SorLA stain (blue). **(C)** Surface SorLA expression as % of total by flow cytometry. Surface SorLA expression = (surface SorLA MFI/total SorLA MFI) × 100. *n* = 9 independent Ramos-Cas9 transductions with three NTg sgRNAs (indicated by symbols) from three independent experiments. Data show mean ± SEM. **(D)** Quantification of SorLA-EGFP colocalization with organelle markers. Voxel-based Manders’ coefficient. Rab5: *n* = 31 cells, Rab11: *n* = 90 cells, Rab7: *n* = 60 cells, and LAMP1: *n* = 41 cells from 10 images from two independent experiments. **(E)** Quantification of endogenous SorLA colocalization with organelle markers at 5, 15, 30, and 60 min after addition to PMS presenting anti-IgM surrogate antigen. Manders’ coefficient. Rab5: *n* = 13–31 cells, Rab11: *n* = 7–29 cells, Rab7: *n* = 7–31 cells, and LAMP1: *n* = 27–58 cells from four independent experiments. These intracellular compartments are not mutually exclusive; thus, SorLA puncta may be positive for several markers and be counted under >1 organelle. Data are shown as box and whisker plots, where the line indicates the median, the box represents the interquartile range, and whiskers denote the minimum and maximum values. **(F)** Representative fluorescence micrographs of Ramos-Cas9 cells on PMS presenting anti-IgM surrogate antigen at 15 min. Images show SorLA (magenta), vesicle markers (cyan), and an overlay from a single z-stack slice (ImageJ). “3D” column shows a 3D projection of the overlay z-stack (Imaris). Colocalization appears white.

SorLA has been described to localize and direct trafficking to a number of distinct cellular compartments, often in a cell type–dependent manner (Fjorback et al., 2012; Hung et al., 2021; Kitago et al., 2015; Larsen and Petersen, 2017; Pietilä et al., 2019; Schmidt et al., 2017). First, to investigate SorLA’s localization in B cells, we established a stable SorLA-EGFP–expressing Ramos B cell line. Confocal microscopy revealed SorLA-EGFP is prominently expressed at the cell surface as well as on intracellular compartments (Fig. 1 A). Using flow cytometry, we quantified the surface and total expression of endogenous SorLA by immunostaining for SorLA N terminus (Fig. 1 B), indicating that around 15.6% of total cellular SorLA is present on the surface of Ramos B cells (Fig. 1 C).

To characterize SorLA’s subcellular organization and its potential role in BCR–antigen intracellular trafficking, we imaged SorLA-EGFP localization in Ramos cells with endolysosomal markers following fluorescent surrogate antigen uptake from plasma membrane sheets (PMS). A median of 48.2% of SorLA-EGFP localized to Rab5^+^ vesicles, associated with early cargo trafficking. On average, 33.1% of SorLA-EGFP localized to Rab11^+^ recycling compartments. 20.2 and 22.8% of SorLA-EGFP localized to late Rab7^+^ and LAMP1^+^ vesicles, associated with antigen processing (Fig. 1 D). To confirm these results, we analyzed the localization of endogenous SorLA by immunostaining at distinct time points over the course of 60 min (Fig. 1, E and F). These data show that at 5 min, the greatest proportion of SorLA is localized to Rab5^+^ and Rab11^+^ vesicles. The proportion of SorLA localized to Rab7^+^ and LAMP1^+^ vesicles increases gradually until 30 min after incubation with PMS. This dynamic pattern is consistent with a role for SorLA in regulating endolysosomal trafficking following BCR stimulation.

### SorLA interacts with the BCR in Rmos cells and primary murine B cells

**Figure 2.**
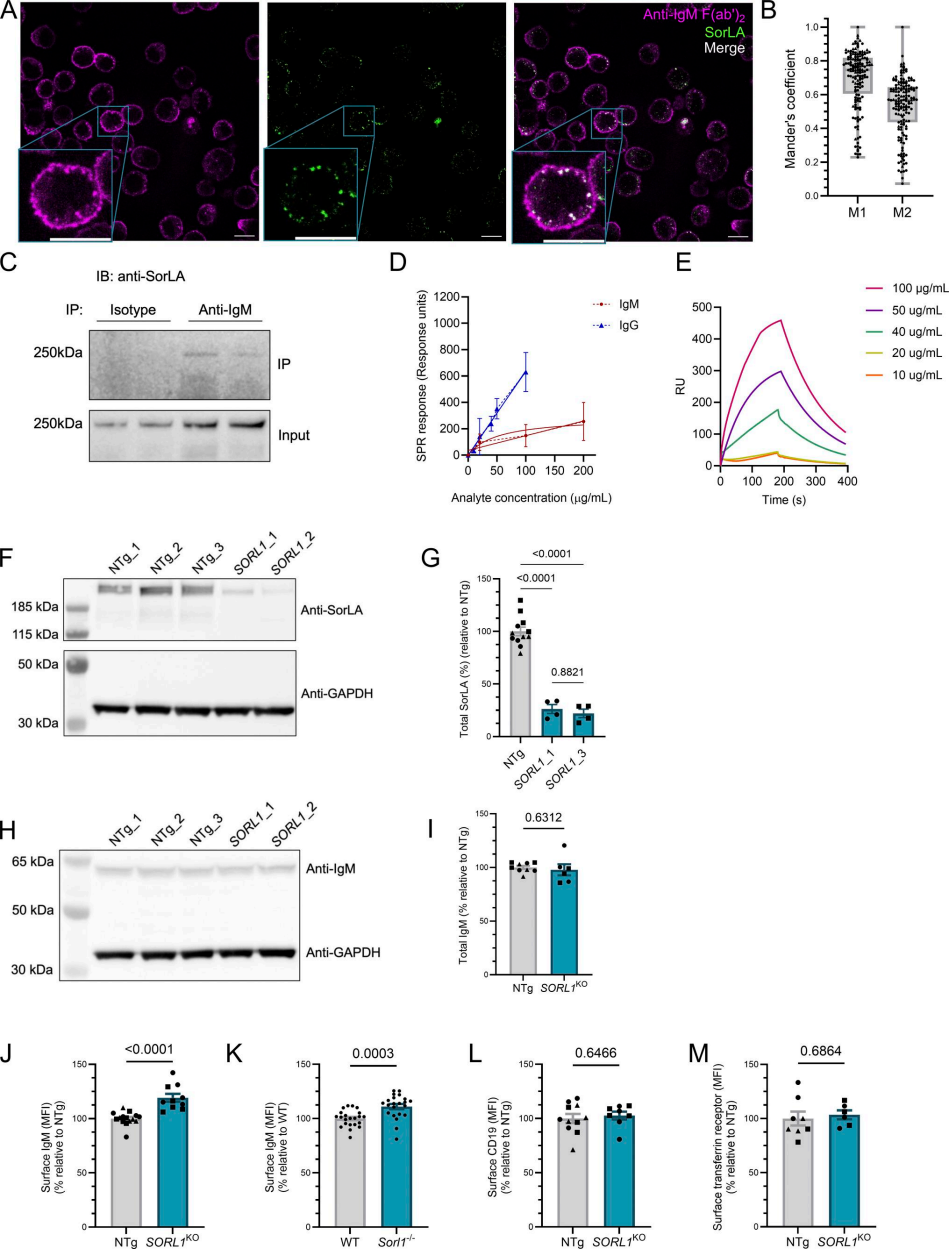
**SorLA interacts with the BCR in Ramos B and primary B cells. (A)** Representative confocal micrographs of SorLA (green) and anti-IgM surrogate antigen (magenta) following 5-min incubation at 37°C. 100× magnification; inset (blue box) shows an enlarged view of an individual cell. Colocalization appears white. Scale bars = 10 µm. **(B)** Quantification of A by Manders’ colocalization coefficient. *n* = 159 cells from five images. Data are shown as box and whisker plots, where the line indicates the median, the box represents the interquartile range, and whiskers denote the minimum and maximum values. **(C)** Anti-IgM immunoprecipitation in primary B cells. Representative immunoblot of two independent experiments. Cell lysates were incubated with biotinylated anti-IgM F(ab′)_2_ or biotinylated anti-CD43, as a negative control not expressed in B cells, and Dynabeads M-280 Streptavidin. Immunoprecipitation eluate was analyzed by western blot (probed with anti-SorLA antibody). *n* = 2 of two pooled mice per sample. **(D)** SPR response at different concentrations of human IgM (red) and IgG (blue) to immobilized His-tagged SorLA (captured at 10 µg/ml). Two independent experiments. **(E)** Representative SPR sensorgram showing blank- and reference cell–subtracted data for concentration-dependent binding of human Ig to immobilized His-tagged SorLA (captured at 10 µg/ml). Increasing analyte concentrations (indicated) were injected over the sensor surface, and binding responses were recorded in response units (RU) over time. The approximate dissociation constants were determined to be *K*_*D*_ ≈ 495–668 nM and 1.84–2.2 µM for IgG and IgM, respectively. **(F)** Representative *SORL1* knockout immunoblot showing total SorLA protein expression in Ramos-Cas9 cell lines expressing one of three different NTg sgRNAs or two sgRNAs targeting *SORL1*. GAPDH used as protein-loading control. **(G)** Quantification of SorLA protein depletion per sgRNA-transduced cell line (indicated by symbols) relative to mean SorLA expression in NTg Ramos-Cas9 cell lines per experiment. SorLA depletion quantified by densitometry of *SORL1* knockout immunoblots. *n* = 4–12 independent Ramos-Cas9 transductions with three NTg sgRNAs and two *SORL1*-targeting sgRNAs (indicated by symbols), quantified from four immunoblots. Data show mean ± SEM. P < 0.0001 calculated by unpaired *t* test. **(H)** Representative immunoblot showing total IgM expression in NTg and *SORL1*^KO^ Ramos-Cas9 cell lines. GAPDH used as protein-loading control. **(I)** Densitometry quantification of total IgM expression in NTg and *SORL1*^KO^ Ramos-Cas9 cell lines relative to mean total IgM expression in NTg Ramos-Cas9 cell lines per experiment. *n* = 6–9 independent Ramos-Cas9 transductions with three NTg sgRNAs and two *SORL1*-targeting sgRNAs (indicated by symbols) quantified from three immunoblots. **(J)** Surface BCR expression for NTg and *SORL1*^KO^ Ramos-Cas9 cells relative to mean NTg surface BCR expression per experiment. *n* = 10–15 from five independent experiments. **(K)** Surface BCR expression for WT and *Sorl1*^−/−^ cells relative to mean WT surface BCR expression per experiment. *n* = 16-17 mice from three independent experiments. **(L)** Surface CD19 expression for NTg and *SORL1*^KO^ Ramos-Cas9 cells relative to mean NTg surface CD19 expression per experiment. *n* = 8–11 independent Ramos-Cas9 transductions with three NTg sgRNAs and two *SORL1*-targeting sgRNAs (indicated by symbols) from four independent experiments. **(M)** Surface transferrin receptor expression for NTg and *SORL1*^KO^ Ramos-Cas9 cell lines relative to mean NTg surface transferrin receptor expression per experiment. *n* = 6–8 independent Ramos-Cas9 transductions with three NTg sgRNAs and two *SORL1*-targeting sgRNAs (indicated by symbols) from three independent experiments. **(I–M)** Data show mean ± SEM. P < 0.0001 calculated by unpaired *t* test. Source data are available for this figure: SourceData F2.

Given this pattern of localization, we next sought to understand the interaction of endogenous cell surface SorLA with the BCR and internalized antigens. We stimulated Ramos cells, which exhibit intact BCR endocytosis and signalling (Gomes de Castro et al., 2019; Rex et al., 2015), with fluorescent anti-IgM F(ab′)_2_ as surrogate antigen. Endogenous SorLA and surrogate antigen localization was visualized after 5 min at 37°C (Fig. 2 A), and colocalization analysis was conducted using the JaCoP ImageJ plug-in (Bolte and Cordelières, 2006), manually excluding cell circumferences from analysis to ensure only internalized surrogate antigen was analyzed. 69.7% of SorLA colocalized with internalized anti-IgM (Manders’ coefficient, M1), while 52.9% of the endocytosed anti-IgM colocalized with SorLA (M2) (Fig. 2 B). To further characterize the potential interaction of endogenous SorLA with the BCR, we conducted co-immunoprecipitation experiments in isolated resting splenic B cells from WT mice. This revealed a stable interaction between a proportion of SorLA molecules and the BCR (Fig. 2 C) and suggests the interaction does not depend on BCR antigen engagement. To further investigate this interaction, we performed surface plasmon resonance (SPR) analysis to assess binding of human IgM and IgG to SorLA (Fig. 2, D and E). Both immunoglobulins exhibited concentration-dependent binding responses, consistent with a direct interaction. The recombinant His-tagged SorLA used in these experiments lacks the cytoplasmic domain, indicating that binding is mediated via the ectodomain.

### SorLA regulates BCR endocytosis in human Ramos cells and primary murine B cells

To investigate SorLA’s specific role in B cell antigen uptake and trafficking, we deleted *SORL1* using two independent sgRNAs in Ramos cells stably expressing *Streptococcus pyogenes* Cas9. In addition to the *SORL1*^KO^ cell lines, we established three non-targeting sgRNA (NTg) cell lines as WT controls.

We confirmed SorLA depletion by immunoblot (Fig. 2 F), showing a 75.9% decrease in protein expression in the population (Fig. 2 G). *SORL1*^KO^ Ramos-Cas9 B cells expressed comparable amounts of total IgM relative to the NTg Ramos-Cas9 B cell population (Fig. 2 H), as quantified by densitometry (Fig. 2 I). While total IgM BCR expression was similar, SorLA deletion resulted in significantly increased surface IgM BCR levels in both Ramos-Cas9 B cells (Fig. 2 J) and primary B cells from *Sorl1*^−/−^ mice (Fig. 2 K). Surface expression of other receptors tested, including CD19 and transferrin receptor, were comparable with that of NTg Ramos-Cas9 B cells (Fig. 2, L and M). Since the reduction in surface IgM BCR levels was not attributable to differences in total IgM expression, we hypothesized that they reflect altered endocytosis, recycling or degradation. Therefore, we next sought to investigate IgM uptake and trafficking in Ramos-Cas9 B cell lines and primary B cells from *Sorl1*^−/−^ mice.

**Figure 3.**
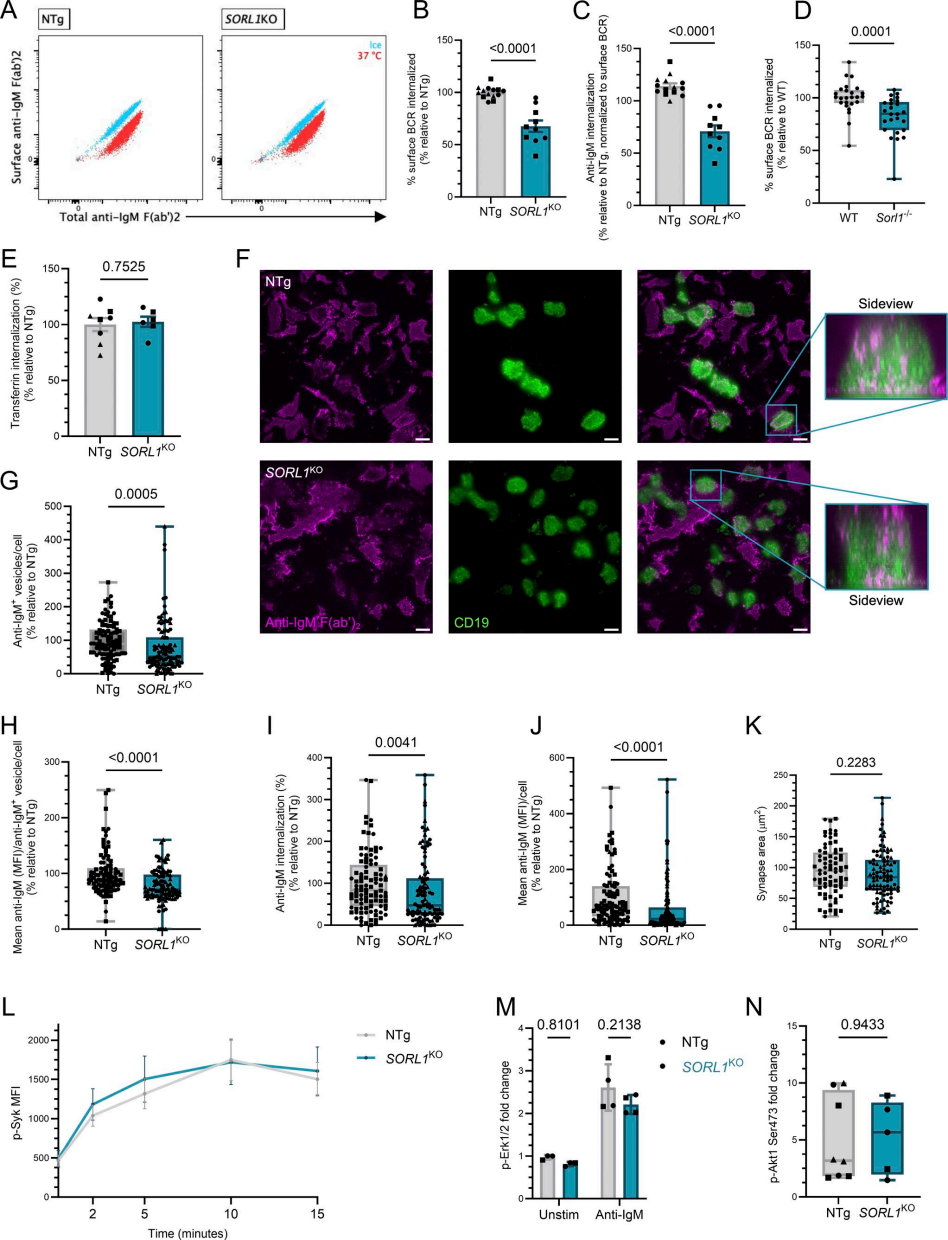
**SorLA regulates BCR uptake in Ramos-Cas9 and primary murine B cells. (A)** Representative flow cytometry dot plots showing anti-IgM F(ab′)_2_ internalization in NTg and *SORL1*^KO^ Ramos-Cas9 cells. Surface (streptavidin fluorescence) and total surrogate antigen (anti-IgM fluorescence) stains of single live cells analyzed by flow cytometry following 15-min incubation on ice (blue) or at 37°C (red). **(B)** Quantification of soluble anti-IgM uptake using % Internalization calculation $\left(\%\;\text{internalization}=100\;\left[1-\left(\text{Ice}\;\text{surface}÷37℃\;\text{surface}\right)\right]\right)$ in NTg and *SORL1*^KO^ Ramos-Cas9 cell lines relative to mean NTg % anti-IgM internalization per experiment. *n* = 10–14 independent Ramos-Cas9 transductions with three NTg sgRNAs and two *SORL1*-targeting sgRNAs (indicated by different symbols) from five independent experiments. **(C)** Soluble anti-IgM surrogate antigen uptake in NTg and *SORL1*^KO^ Ramos-Cas9 cell lines normalized to the initial surface IgM level (MFI at time = 0 on ice). *n* = 10–14 independent Ramos-Cas9 transductions with three NTg sgRNAs and two *SORL1*-targeting sgRNAs (indicated by different symbols) from five independent experiments. **(D)** Soluble anti-IgM surrogate antigen uptake in WT and *Sorl1*^−/−^ primary murine B cells (CD43^−^). Quantified using % internalization calculation and normalized relative to mean WT % anti-IgM internalization per experiment. P value shown calculated by Mann–Whitney U test, *n* = 19–20 mice from four independent experiments. **(E)** Soluble transferrin internalization using % internalization calculation for NTg and *SORL1*^KO^ Ramos-Cas9 cell lines relative to mean NTg % transferrin internalization per experiment. *n* = 6–8 independent Ramos-Cas9 transductions with three NTg sgRNAs and two *SORL1*-targeting sgRNAs (indicated by different symbols) from three independent experiments. **(F)** Representative fluorescence micrographs showing NTg (top) and *SORL1*^KO^ (bottom) Ramos-Cas9 anti-IgM F(ab′)_2_ surrogate antigen uptake from PMS. Anti-IgM-loaded PMS (left), Ramos-Cas9 cells interacting with PMS (center), merge (right), magnified side view 3D projection (blue box). 100× magnification; scale bars = 10 μm. **(G)** Number of anti-IgM^+^ vesicles per cell in NTg and *SORL1*^KO^ Ramos-Cas9 cell lines relative to mean NTg per experiment. **(H)** Mean anti-IgM fluorescence per anti-IgM^+^ vesicle per cell for NTg and *SORL1*^KO^ Ramos-Cas9 cell lines relative to mean NTg per experiment. **(I)** Anti-IgM internalization as a percentage of total anti-IgM at the synapse per cell for NTg and *SORL1*^KO^ Ramos-Cas9 cell lines relative to mean NTg cell lines. **(J)** Mean total anti-IgM fluorescence representing total amount internalized per cell for NTg and *SORL1*^KO^ cell lines relative to mean NTg per experiment. **(K)** Synapse area of NTg and *SORL1*^KO^ Ramos-Cas9 cells at contact with PMS at 15 min, measured using Analyze particles macro (ImageJ). *n* = 74–95 cells in three independent experiments. **(L)** Syk phosphorylation over 15 min at 37°C per phospho-flow time course experiment, *n* = 2–3 per experiment, two independent experiments. Background MFI subtraction using secondary only control. **(M)** p-Erk1/2 fold change in NTg and *SORL1*^KO^ Ramos-Cas9 B cell lines, relative to unstimulated. p-Erk1/2 fold change quantified by densitometry of a p-Erk1/2 immunoblot. *n* = 4. **(N)** p-Akt1 Ser473 phospho-flow. p-Akt1 Ser473 fold change in NTg and *SORL1*^KO^ Ramos-Cas9 B cell lines at 15 min, relative to 0-min time point. P value shown calculated by Mann–Whitney U test, *n* = 5–8 from three independent experiments. Data in B, C, E, L, and M show mean ± SEM. P values are calculated by unpaired *t* test. Data in D and G–K are shown as box and whisker plots, where the line indicates the median, the box represents the interquartile range, and whiskers denote the minimum and maximum values. P values calculated by Mann–Whitney U test, *n* = 100 cells from two independent experiments.

To investigate SorLA’s role in B cell soluble antigen uptake, we performed an established flow cytometry based uptake assay using biotinylated, fluorescent anti-IgM F(ab′)_2_ as surrogate antigen in the *SORL1*^KO^ and NTg Ramos-Cas9 B cell lines (Fig. 3 A). Percentage antigen internalization was calculated using a fluorescent streptavidin counterstain to bind remaining surface antigen (Fig. 3 A). *SORL1*^KO^ Ramos-Cas9 B cells internalized significantly less soluble surrogate antigen compared with NTg cell lines (Fig. 3 B). When normalized to the initial surface IgM BCR levels, *SORL1*^KO^ cells exhibited reduced internalization despite higher surface BCR (Fig. 3 C). Similar to *SORL1*^KO^ Ramos-Cas9 B cells, primary B cells from *Sorl1*^−/−^ mice internalized significantly less surrogate antigen relative to WT murine B cells (Fig. 3 D). In contrast, transferrin receptor internalization was not affected in *SORL1*^KO^ cell lines, which internalized comparable amounts of transferrin relative to NTg cell lines (Fig. 3 E). This indicates that the BCR endocytosis defects observed are not due to a global disruption in endocytosis.

Most physiologically relevant antigens are recognized and physically extracted from immune synapses with antigen-presenting cells (Batista et al., 2001; Carrasco and Batista, 2007; Kosco-Vilbois et al., 1993; Natkanski et al., 2013). To define SorLA’s role in surface-bound antigen uptake in B cells, we used PMS, a model mimicking the cell–cell interface, to visualize flexible membrane-bound surrogate antigen uptake (Fig. 3 F). Ramos B cell antigen extraction from PMS was analyzed and quantified using Analyze Internalization GUI, an image analysis tool to measure the amount of ligand that cells extract and internalize from planar substrates (Nowosad and Tolar, 2017). *SORL1*^KO^ Ramos-Cas9 B cells formed significantly fewer antigen-positive vesicles (Fig. 3 G), containing less antigen per vesicle (Fig. 3 H). Surrogate antigen internalization from PMS was significantly reduced in *SORL1*^KO^ relative to NTg Ramos-Cas9 B cells, both as a percentage of total antigen at the synapse (Fig. 3 I) and as a total amount internalized (Fig. 3 J). This reduced antigen accumulation did not result from defective cell spreading, as synapse area was comparable between *SORL1*^KO^ and NTg cells (Fig. 3 K).

**Figure S1.**
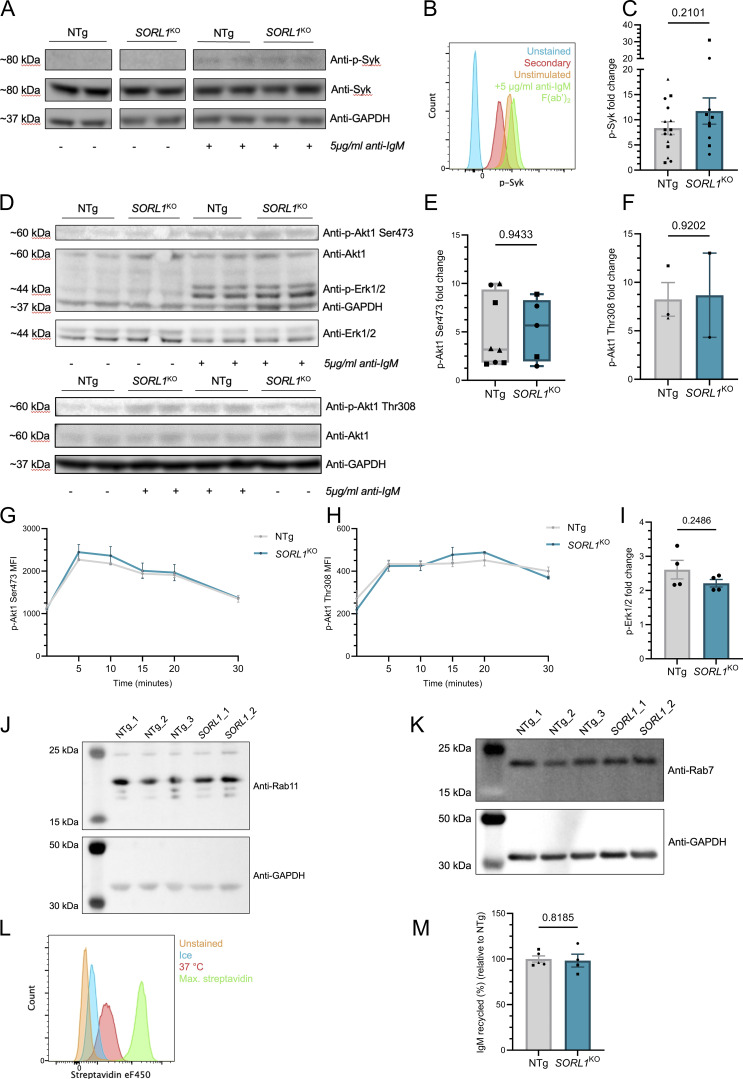
**SorLA does not play a significant role in BCR signalling or recycling. (A)** Representative immunoblot showing total and phosphorylated Syk (p-Syk) expression in NTg and *SORL1*^KO^ Ramos-Cas9 B cell lines following 5-min incubation at 37°C with or without 5 μg/ml F(ab′)_2_ anti-IgM stimulation. GAPDH used as protein-loading control. **(B)** Flow cytometry population histograms for phospho-Syk showing unstained (blue), secondary antibody only (red), unstimulated (orange), and stimulated (green). **(C)** p-Syk phospho-flow. p-Syk fold change in NTg and *SORL1*^KO^ Ramos-Cas9 cells at 5 min, relative to 0-min time point. *n* = 10–15 independent Ramos-Cas9 transductions with three NTg sgRNAs and two *SORL1*-targeting sgRNAs (indicated by different symbols) from six independent experiments. **(D)** Representative immunoblot showing total and phosphorylated Akt1 (Ser473 and Thr308) and Erk1/2 expression in NTg and *SORL1*^KO^ Ramos-Cas9 B cell lines following 15-min incubation at 37°C with or without 5 μg/ml F(ab′)_2_ anti-IgM stimulation. GAPDH used as protein-loading control. **(E)** p-Akt1 Ser473 fold change in NTg and *SORL1*^KO^ Ramos-Cas9 B cell lines, relative to unstimulated. p-Akt1 Ser473 fold change quantified by densitometry of a p-Akt1 Ser473 immunoblot. *n* = 5–8 in two independent immunoblots. P value shown are calculated by Mann–Whitney U test. **(F)** p-Akt1 Thr308 fold change in NTg and *SORL1*^KO^ Ramos-Cas9 cell lines, relative to unstimulated. p-Akt1 Thr308 fold change quantified by densitometry of a p-Akt1 Thr308 immunoblot. *n* = 2–3. **(G)** Akt1 phosphorylation at Ser473 over 30 min per phospho-flow time course experiment, *n* = 2–3 per experiment, two independent experiments. Background MFI subtraction using secondary only control. **(H)** Akt1 phosphorylation at Thr308 over 30 min per phospho-flow time course experiment, *n* = 2–3 per experiment, two independent experiments. Background MFI subtraction using secondary only control. **(I)** p-Erk1/2 fold change in NTg and *SORL1*^KO^ Ramos-Cas9 B cell lines, relative to unstimulated. p-Erk1/2 fold change quantified by densitometry of a p-Erk1/2 immunoblot. *n* = 4. **(J)** Immunoblot showing total Rab11 expression in NTg and *SORL1*^KO^ Ramos-Cas9 cell lines. GAPDH used as protein-loading control. **(K)** Immunoblot showing total Rab7 expression in NTg and *SORL1*^KO^ Ramos-Cas9 cell lines. GAPDH used as protein-loading control. **(L)** Flow cytometry population histograms for unstained (orange), ice (blue), 37°C (red), and maximum streptavidin (green). **(M)** Recycled IgM in NTg and *SORL1*^KO^ Ramos-Cas9 cell lines relative to mean NTg surface recycled IgM per experiment. P value shown are calculated by unpaired *t* test, *n* = 4–5 from two independent experiments. P values shown in C, F, and I are calculated by unpaired *t* test. Data in C, F–I, and M show mean ± SEM. Source data are available for this figure: SourceData FS1.

Dysregulated surface BCR levels can result in altered downstream signalling (Coulter et al., 2018; Davis et al., 2010). The increased surface BCR levels following *SORL1* deletion led us to investigate antigen-induced BCR signalling through early Syk phosphorylation and downstream Akt1 and Erk1/2 phosphorylation in the Ramos-Cas9 B cell lines. While there was some evidence of a modest increase in Syk phosphorylation after anti-IgM–induced BCR engagement following SORL1 deletion, this did not reach statistical significance, suggesting limited direct effects on early BCR signalling (Fig. 3 L) (Fig. S1, A–C). Further, no differences were detected in Erk and Akt phosphorylation in the downstream signalling cascade (Fig. 3, M and N; and Fig. S1, D–I).

These results confirm SorLA’s important role in B cell antigen uptake, an essential B cell process for acquisition of antigens for presentation. As both soluble antigen uptake and extraction from PMS are affected by SorLA depletion, we hypothesize this is not due to differences in contractile strength at the B cell synapse, but instead to a previously undescribed role of SorLA in BCR endocytosis or trafficking.

### SorLA regulates antigen-induced BCR degradation in Ramos-Cas9 cells

**Figure 4.**
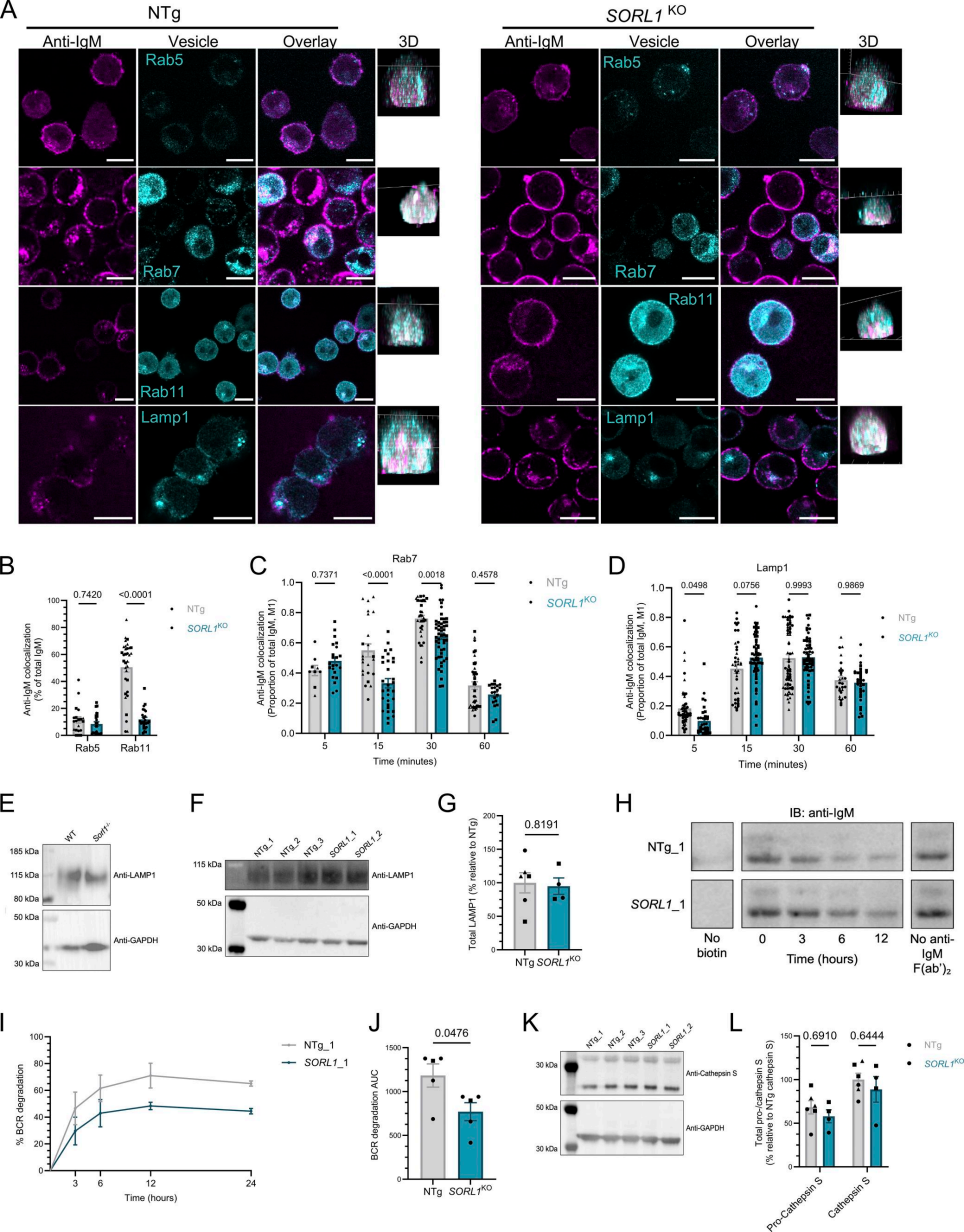
**SorLA regulates antigen-induced BCR degradation in Ramos-Cas9 cells. (A)** Representative confocal micrographs of anti-IgM surrogate antigen (magenta) and distinct vesicle markers (cyan) following 15-min incubation at 37°C. 100× magnification. Images show individual fluorescent channels and an overlay from a single z-stack slice (ImageJ). 3D column shows a 3D projection of the overlay z-stack (Imaris). Colocalization appears white. Scale bars = 10 µm. **(B)** Quantification of anti-IgM colocalization with organelle markers in NTg and *SORL1*^KO^ Ramos-Cas9 cell lines following surrogate antigen stimulation for 15 min. Manders’ coefficient. Rab5: *n* = 20–23 cells, Rab11: *n* = 25–33 cells. Three independent experiments. **(C)** Quantification of anti-IgM colocalization with Rab7 in NTg and *SORL1*^KO^ Ramos-Cas9 cell lines following surrogate antigen stimulation for indicated times. Manders’ coefficient. *n* = 9–51 cells in three independent experiments. **(D)** Quantification of anti-IgM colocalization with LAMP1 in NTg and *SORL1*^KO^ Ramos-Cas9 cell lines following surrogate antigen stimulation for indicated times. Voxel-based Manders’ coefficient. *n* = 29–68 cells in three independent experiments. **(E)** Representative immunoblot showing total LAMP1 expression in WT and *SORL1*^KO^ mouse splenic B cells. GAPDH used as protein-loading control. **(F)** Representative immunoblot showing total LAMP1 expression in NTg and *SORL1*^KO^ Ramos-Cas9 cell lines. GAPDH used as protein-loading control. **(G)** Densitometry quantification of total LAMP1 expression in NTg and *SORL1*^KO^ Ramos cell lines. *n* = 4–6 independent Ramos-Cas9 transductions with three NTg sgRNAs and two *SORL1*-targeting sgRNAs (indicated by different symbols) quantified from two immunoblots. P values shown are calculated by unpaired *t* test. **(H)** Representative immunoblot showing BCR degradation in NTg and *SORL1*^KO^ Ramos-Cas9 cell lines over 12 h following surrogate antigen stimulation. No biotin and no anti-IgM controls are included at 6 h. **(I)** % BCR degradation calculation using densitometry values from BCR degradation immunoblots (*n* = 5, three independent experiments) $\left(\%\;\text{BCR}\;\text{degradation}=100\;\left[1-\left(\text{Timepoint}÷0\;\text{hours}\right)\right]\right)$ in NTg and *SORL1*^KO^ Ramos-Cas9 cell lines over 24 h following surrogate antigen stimulation. **(J)** Quantification of the area under curve shown in C relative to NTg cells per experiment. Distinct sgRNAs are indicated by symbols. **(K)** Representative immunoblot showing total pro-cathepsin S (37 kDa) and cathepsin S (24 kDa) expression in NTg and *SORL1*^KO^ Ramos-Cas9 cell lines. GAPDH is used as protein-loading control. **(L)** Quantified total pro-cathepsin S and cathepsin S expression in NTg and *SORL1*^KO^ Ramos-Cas9 cell lines. Total pro-cathepsin S and cathepsin expression quantified by densitometry of total two independent pro-cathepsin S and cathepsin immunoblots. *n* = 4–6. P values shown in B, C, D, and L are calculated by two-way ANOVA. P values shown in G and J are calculated by unpaired *t* test. Data show mean ± SEM. Source data are available for this figure: SourceData F4.

We next investigated the role of SorLA in BCR–antigen intracellular trafficking using the CRISPR/Cas9-engineered NTg and *SORL1*^KO^ Ramos cells. Cells internalized fluorescent surrogate antigen from PMS and were then immunostained for endolysosomal markers to determine antigen localization to distinct cellular compartments (Fig. 4 A). Colocalization analysis revealed no difference in quantity of antigen observed in early endosomes (Rab5^+^ [Wandinger-Ness and Zerial, 2014]) (Fig. 4 B). However, *SORL1*^KO^ Ramos-Cas9 B cells exhibited a downstream trafficking defect through Rab11^+^ and Rab7^+^ compartments, suggesting SorLA may play a role in both BCR recycling and downstream BCR–antigen trafficking through degradative lysosomal compartments (Fig. 4, C and D). Total expression levels of Rab11 and Rab7 were not affected by SorLA disruption (Fig. S1, J and K). Total LAMP1 protein expression was also similar between *SORL1*^KO^ and NTg Ramos cell lines and between WT and *Sorl1*^−/−^ murine B cells (Fig. 4, E–G).

To understand the extent to which recycling was affected in the absence of SorLA, we quantified anti-IgM–bound BCR recycling dynamics by flow cytometry. In this assay, surrogate antigen remaining on the cell surface is counterstained with streptavidin, followed by a further incubation at 37°C to allow recycling. BCR–antigen complexes recycled to the surface are then identified using streptavidin conjugated to a different fluorophore. No significant difference was observed in BCR recycling, suggesting reduced antigen localization to Rab11^+^ compartments may not affect overall dynamics of recycling (Fig. S1, L and M).

To investigate the role of SorLA in lysosomal BCR–antigen trafficking and in antigen processing, we carried out a BCR degradation assay in *SORL1*^KO^ Ramos-Cas9 B cells. Briefly, cells were surface biotinylated and stimulated with anti-IgM to induce BCR internalization. Cell lysates were taken at defined time points, and biotinylated proteins were pulled down with streptavidin-coated magnetic beads. Eluted proteins were analyzed by immunoblot, probing membranes for IgM to quantify % BCR degradation by densitometry (Fig. 4, H and I). *SORL1*^KO^ Ramos-Cas9 B cells were less efficient at degrading antigen–BCR complexes, following surrogate antigen stimulation, over a 24-h period (Fig. 4 I). The cumulative effect is quantified as area under the curve over this time course in Fig. 4 J.

**Figure S2.**
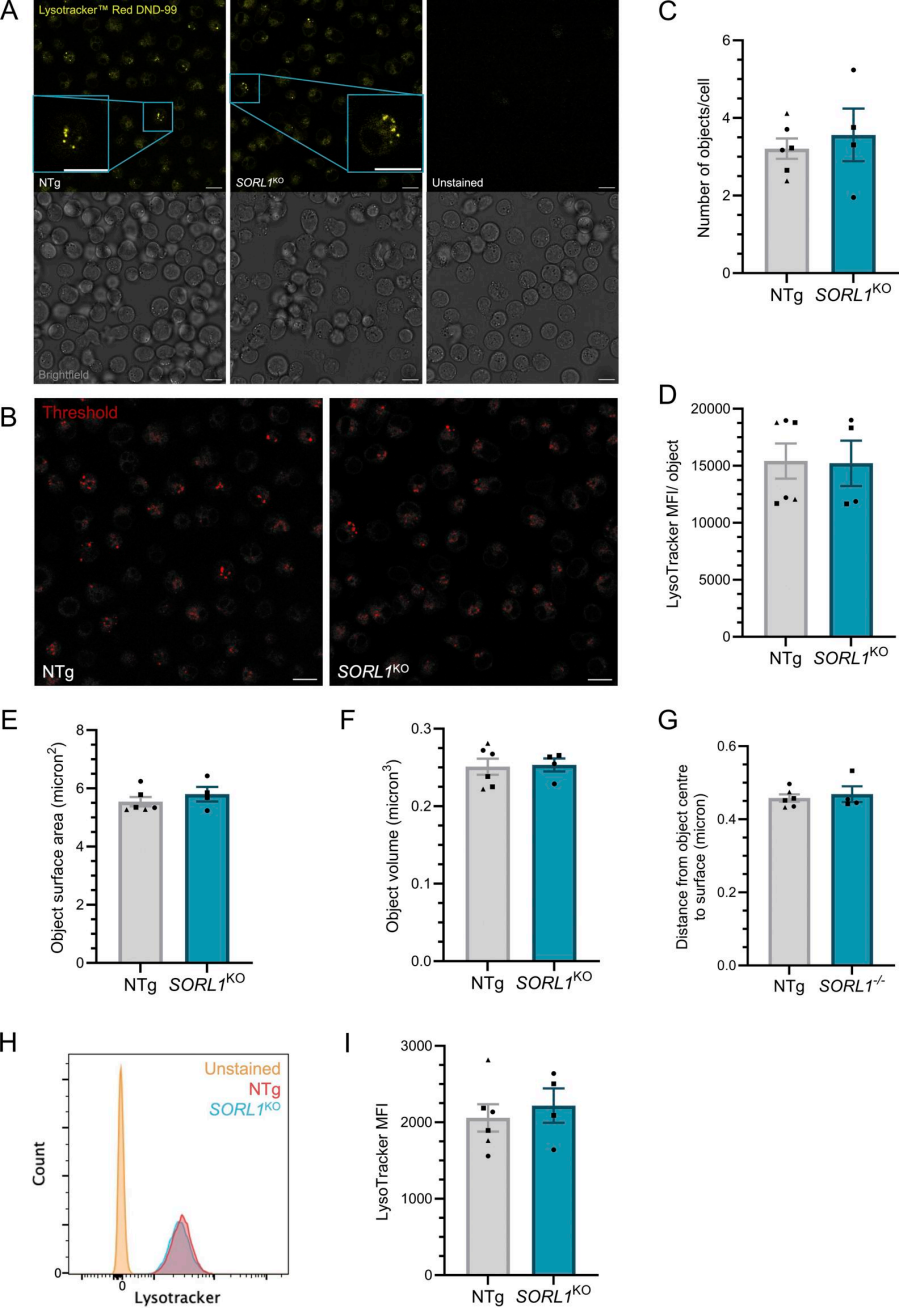
**SorLA does not play a role in lysosome maintenance in Ramos-Cas9 B cells. (A)** Confocal micrographs showing NTg and *SORL1*^KO^ Ramos-Cas9 B cell lines labelled with LysoTracker Red DND-99 (yellow). NTg (left), SORL*1*^KO^ (center), and unstained cells (right); inset (blue box) shows an enlarged view of an individual cell. 100× magnification; scale bars = 10 μm. Corresponding bright-field micrographs below. **(B)** 3D objects analysis thresholding of A. 3D objects analysis detected LysoTracker-positive objects 10–280 voxels in size (∼500–1,500 nm diameter). **(C)** Mean number of LysoTracker-positive objects per cell in NTg and *SORL1*^KO^ Ramos-Cas9 cell lines. **(D)** MFI of LysoTracker-positive objects per cell in NTg and *SORL1*^KO^ Ramos-Cas9 cell lines. **(E)** Mean surface area of LysoTracker-positive objects per cell in NTg and *SORL1*^KO^ Ramos-Cas9 cell lines. **(F)** Mean object volume of LysoTracker-positive objects per cell in NTg and *SORL1*^KO^ Ramos-Cas9 cell lines. **(G)** Mean distance from center to surface of LysoTracker-positive objects per cell in NTg and *SORL1*^KO^ Ramos- Cas9 cell lines. **(H)** Flow cytometry population histograms for unstained (orange), NTg (blue), and *SORL1*^KO^ (red) I LysoTracker MFI in NTg and *SORL1*^KO^ Ramos-Cas9 cell lines. **(I)** Quantification of LysoTracker MFI in NTg and *SORL1*^KO^ Ramos-Cas9 cell lines. *n* = 4–6 independent Ramos-Cas9 transductions with three NTg sgRNAs and two *SORL1*-targeting sgRNAs (indicated by different symbols). **(C–G)***n* = 4–6, each the mean of ∼100 cells from six images from two independent experiments. Data show mean ± SEM.

To assess whether this reduction in BCR degradation was due to lysosome dysfunction, we characterized lysosomes in *SORL1*^KO^ Ramos-Cas9 B cells using LysoTracker imaging with 3D objects analysis, and quantification of lysosome-associated protein expression. *SORL1*^KO^ and NTg Ramos-Cas9 B cells had similar numbers of LysoTracker-positive vesicles per cell (Fig. S2, A and B) and exhibited similar LysoTracker mean fluorescent intensity per object (Fig. S2, C and D). Lysosomal vesicles had similar surface area and volume (Fig. S2, E and F), as well as a similar shape (measured by distance from object center to surface values) (Fig. S2 G). Equivalent LysoTracker MFI per cell was observed by flow cytometry (Fig. S2, H and I). Moreover, lysosome-associated protein expression was unchanged upon SorLA deletion in Ramos-Cas9 B cells. Total pro-cathepsin S and cleaved cathepsin S (Fig. 4, K and L), a protease which promotes degradation in the endolysosomal pathway, were unchanged following SorLA deletion. These data point to normal lysosomal structure and function following SorLA disruption in B cells.

### Loss of SorLA does not impact B cell development

Expression studies showing that SorLA is highly expressed in the bone marrow and in lineage^−^ human bone marrow cells (Zhang et al., 2000) led us to investigate SorLA’s role in immune cell development. A functional BCR is required for full B cell development, and as we observed decreased BCR uptake and increased surface BCR expression following *SORL1*/*Sorl1* deletion, we hypothesized that this may alter B cell development *in vivo*. To investigate this, we analyzed developing and mature B cell populations in bone marrow, spleen, and lymph node in *Sorl1*^−/−^ mice, a ubiquitous *Sorl1* knockout, and in CRISPR/Cas9-targeted bone marrow chimeras (hematopoietic deletion of *Sorl1*) to better assess B cell–intrinsic effects.

**Figure S3.**
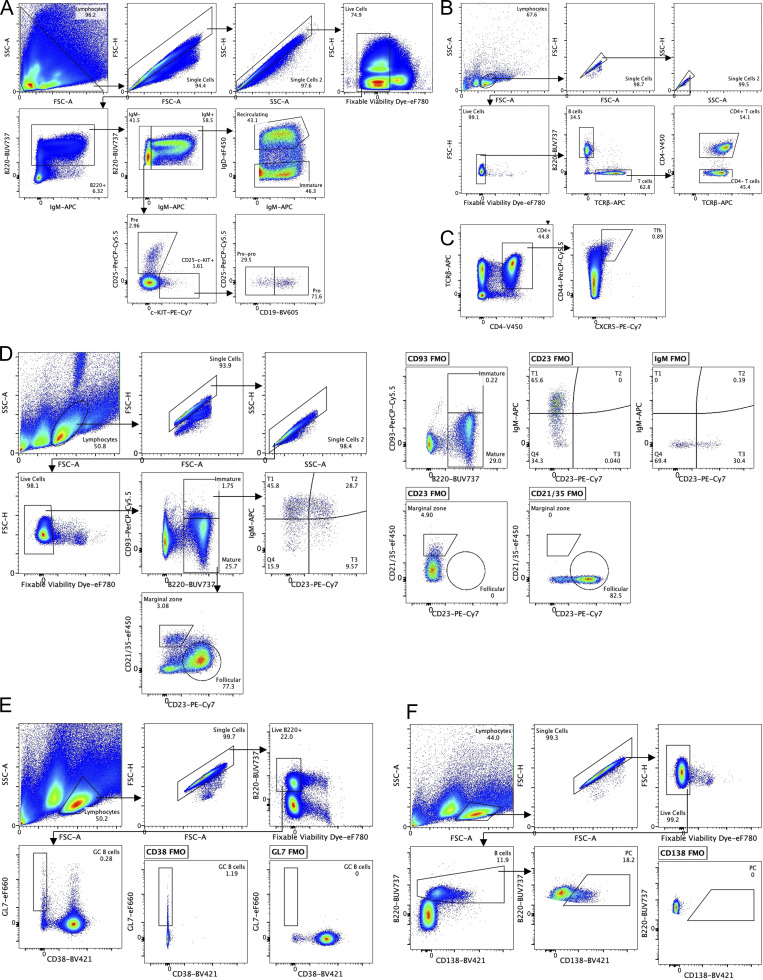
**B cell immunophenotyping gating strategies. (A)** Bone marrow gating strategy for early B cell development and recirculating B cells. **(B and C)** Lymph node gating strategy for B cells, CD4 T cells, and T follicular helper cells. **(D)** Spleen gating strategy for T1, T2, T3, follicular, and marginal zone compartments. **(E)** Gating strategy for GC B cells, showing unimmunized control and immunized WT and *Sorl1*^−/−^ mice. **(F)** Gating strategy for plasma cells. FMO indicates fluorescence minus one control.

B220^+^ B cell populations were identified in bone marrow, spleen, and lymph nodes and characterized based on cell surface expression of stage-specific markers. Full gating strategies are shown in Fig. S3.

**Figure 5.**
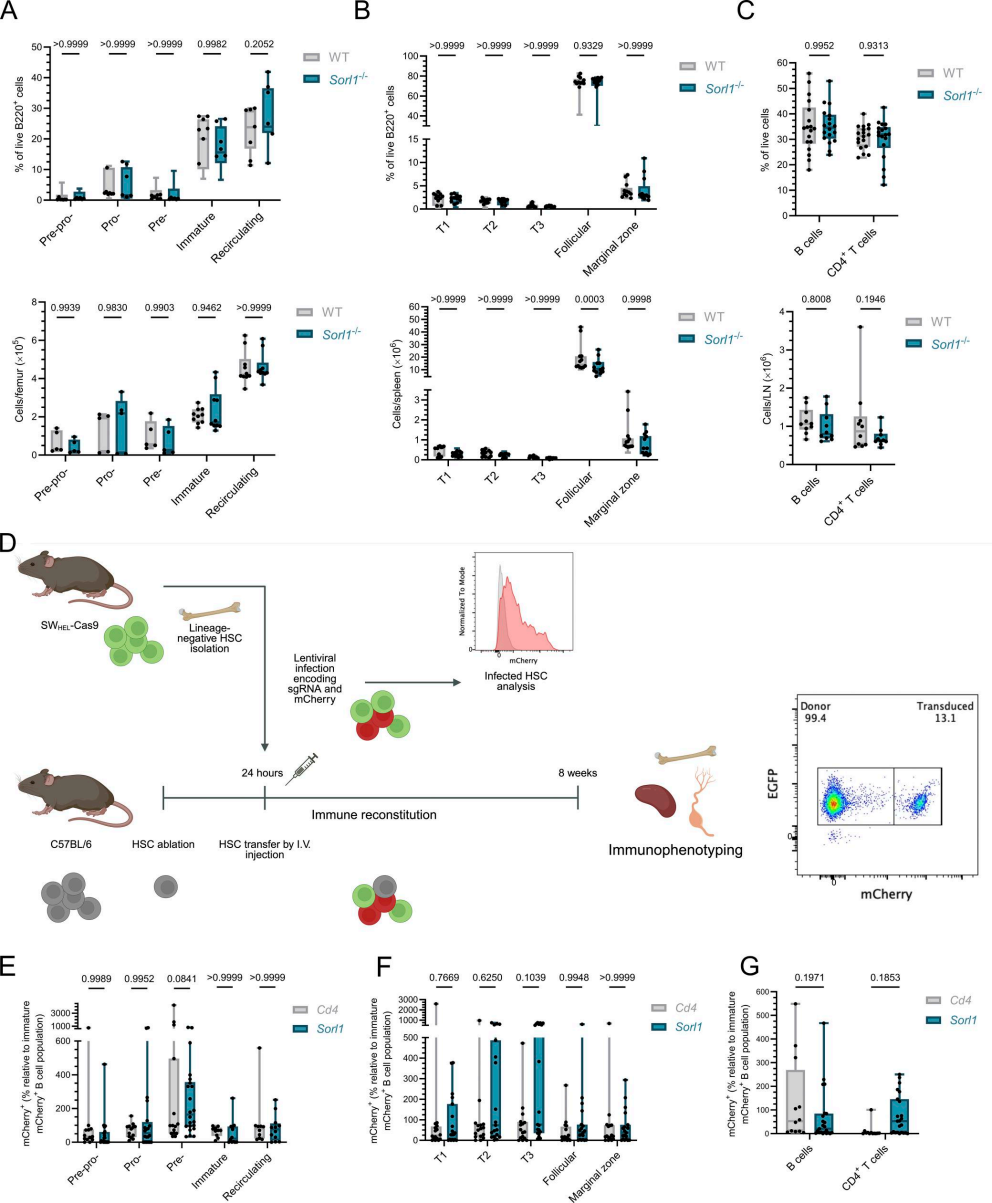
**B cell development is not affected by Sorl1 deletion. (A)** Bone marrow B cell populations in WT and *Sorl1*^−/−^ mice; top graph shows percentage, bottom shows cell counts. *n* = 8–11 mice from three independent experiments. **(B)** Spleen B cell populations in WT and *Sorl1*^−/−^ mice; top graph shows percentage, bottom shows cell counts. *n* = 13 mice from three independent experiments. **(C)** Lymph node lymphocyte populations in WT and *Sorl1*^−/−^ mice; top graph shows percentage, bottom shows cell counts. *n* = 18 mice from four independent experiments. **(D)** Schematic depicting setup and analysis of CRISPR-targeted bone marrow chimeras. **(E)** Development of B cell populations in chimeras’ bone marrow from *Cd4*- or *Sorl1*-targeted HSCs. % mCherry^+^ cells at each stage is normalized to the mean immature mCherry^+^ B cell population for each sgRNA per experiment. *n* = 15–22 mice from four independent experiments. **(F)** Development of B cell populations in chimeras’ spleen from *Cd4*- or *Sorl1*-targeted HSCs. % mCherry^+^ cells at each stage is normalized to the mean immature mCherry^+^ B cell population for each sgRNA per experiment. *n* = 15–22 mice from four independent experiments. **(G)** Development of B and T cell populations in chimeras’ lymph node from *Cd4*- or *Sorl1*-targeted HSCs. % mCherry^+^ cells at each stage is normalized to the mean immature mCherry^+^ B cell population for each sgRNA per experiment. *n* = 12–23 mice from four independent experiments. P values shown in A–C and E–G are calculated by two-way ANOVA. Data are shown as box and whisker plots, where the line indicates the median, the box represents the interquartile range, and whiskers denote the minimum and maximum values.

In *Sorl1*^−/−^ mice, we observed no noticeable differences in developing and recirculating bone marrow B cell populations when compared with WT mice (Fig. 5 A). We also observed no changes in the proportion of different immature (CD93^HI^) and mature (CD93^LO^) splenic B cell populations (Fig. 5 B), but a slight decrease in absolute number of follicular B cells. There were no significant alterations in the lymph node total B cell population (B220^+^) (Fig. 5 C).

To further investigate the role SorLA may play in B cell development, we used a chimeric mouse system (described in detail by Malinova et al., [2021]; Newman and Tolar, [2021]) (Fig. 5 D). In this system, donor hematopoietic stem cells (HSCs) from Cas9 mice are EGFP^+^, and CRISPR-targeted cells also express mCherry, allowing tracking of their expansion or loss, which can result from survival defects and competition from non-targeted cells. Chimeric animals typically exhibit 20–60% untargeted immune cells; thus, B cells can still elicit sufficient help from WT immune populations enabling investigation of B cell–intrinsic effects.

*Sorl1*-targeted mCherry^+^ B cell populations in the bone marrow and transitional populations in spleen remained relatively constant and similar to control *Cd4*-targeted mCherry^+^ B cell populations, indicating that early B cell development progressed normally in SorLA-depleted cells (Fig. 5, E and F). A small increase in the pre-B cell compartment was observed in *Sorl1*-targeted mice, though this did not reach statistical significance (Fig. 5 E). This was supported by normal development of mature B cell populations in the spleen and lymph nodes (Fig. 5 F). *Cd4*-targeted mCherry^+^ CD4^+^ T cell populations in lymph node were depleted, showing excellent gene editing efficiency *in vivo* (Fig. 5 G).

### SorLA promotes efficient B cell antigen presentation

**Figure 6.**
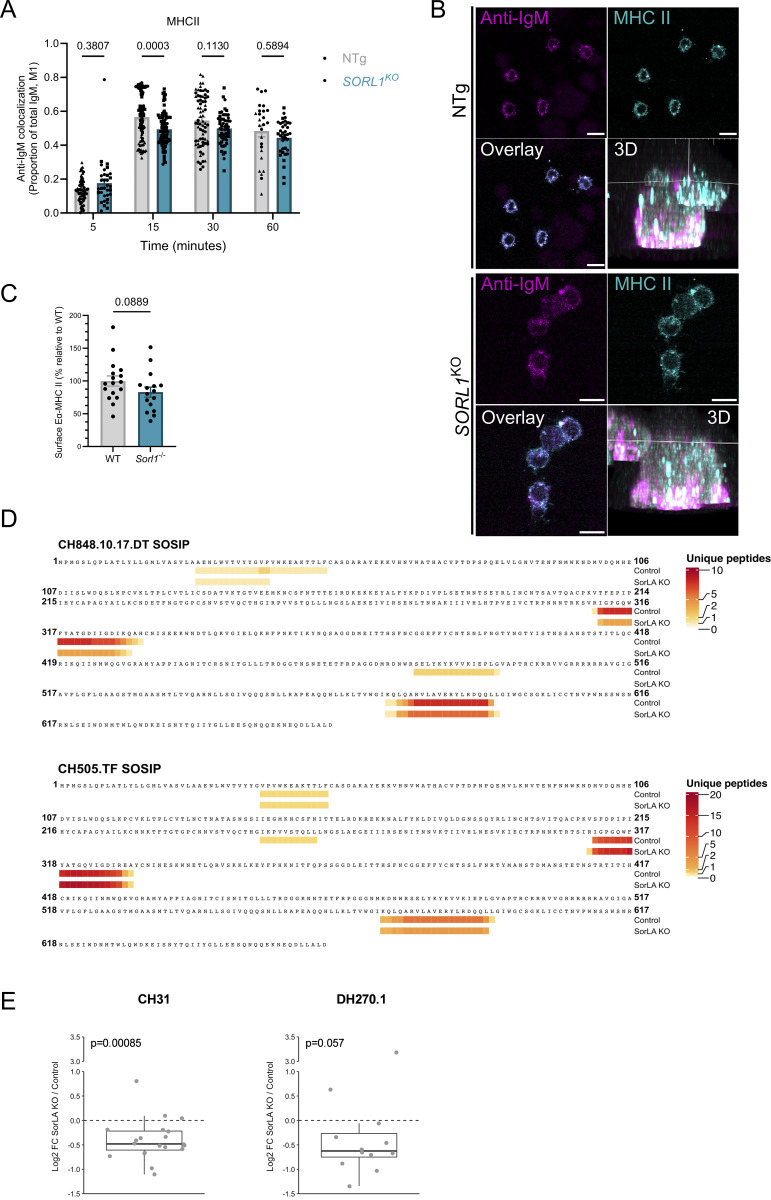
**SorLA contributes to B cell antigen presentation. (A)** Representative fluorescent micrographs of anti-IgM surrogate antigen and MHC II in NTg and *SORL1*^KO^ Ramos-Cas9 cell lines. **(B)** Quantification of anti-IgM colocalization with MHC II molecules in NTg and *SORL1*^KO^ Ramos-Cas9 cell lines. Manders’ coefficient. *n* = 100–120 cells from three independent experiments. P value shown are calculated by two-way ANOVA. **(C)** Surface Eα-MHC II surface expression for WT and *Sorl1*^−/−^ cells relative to mean WT surface Eα-MHC II expression per experiment. *n* = 19–20 mice from four independent experiments. P value shown are calculated by unpaired *t* test. **(D)** HIV-1 Env SOSIP-derived peptides presented with HLA II by *SORL1*^KO^ and NTg control Ramos CH31 and Ramos DH270.1 HIV-1 bnAb IgM BCR-expressing B cell lines pulsed with CH505TFv4.1 SOSIP and CH848 10.17DT SOSIP, respectively. HIV-1 Env peptides identified in the HLA II–bound immunopeptidome of each cell line 24 h after the SOSIP antigen was added are indicated in heatmap format, aligned to the amino acid sequence of the CH505TFv4.1 SOSIP or CH848 10.17DT SOSIP. **(E)** Relative abundance of SOSIP-derived peptides (individual datapoints) presented by *SORL1*^KO^ versus NTg control cells. The peptide intensities in each sample were normalized to the total signal intensity of that sample using the IonQuant package. Relative abundance of peptides was calculated using the normalized signal intensities of each peptide and plotted as the log2 of the ratio of intensities of each peptide in the *SORL1*^KO^ versus NTg cells. P values shown are calculated by Wilcoxon ranked-signed test. Data in B and C show mean ± SEM.

As B cell development was unaffected, we next sought to define mature naive B cell function in *Sorl1*^−/−^ mice. To investigate if the reduction in BCR–antigen uptake and intracellular trafficking to lysosomes affects peptide–MHC II presentation, we first assessed surrogate antigen transport to MHC II loading compartments in the CRISPR/Cas9-engineered NTg and *SORL1*^KO^ Ramos B cells. Cells that had internalized fluorescent surrogate antigen from PMS were fixed and immunostained for MHC II (Fig.6 A). *SORL1*^KO^ Ramos-Cas9 cells exhibited lower colocalization between antigen and MHC II compared with NTg cells.

Next, we directly assessed antigen presentation using the established Eα peptide flow cytometry assay (Ghimire et al., 2012; Itano et al., 2003). We cultured isolated WT and *Sorl1*^−/−^ B cells with Eα peptide conjugated to anti-IgM F(ab′)_2_ by biotin-streptavidin bridge. Anti-IgM facilitates uptake of the complex, and Eα presentation in the context of MHC II is quantified by immunostaining at 16 h after stimulation. Surface levels of Eα-MHC II indicated a trend showing reduced peptide–MHC II presentation in the absence of SorLA (Fig. 6 B).

**Figure S4.**
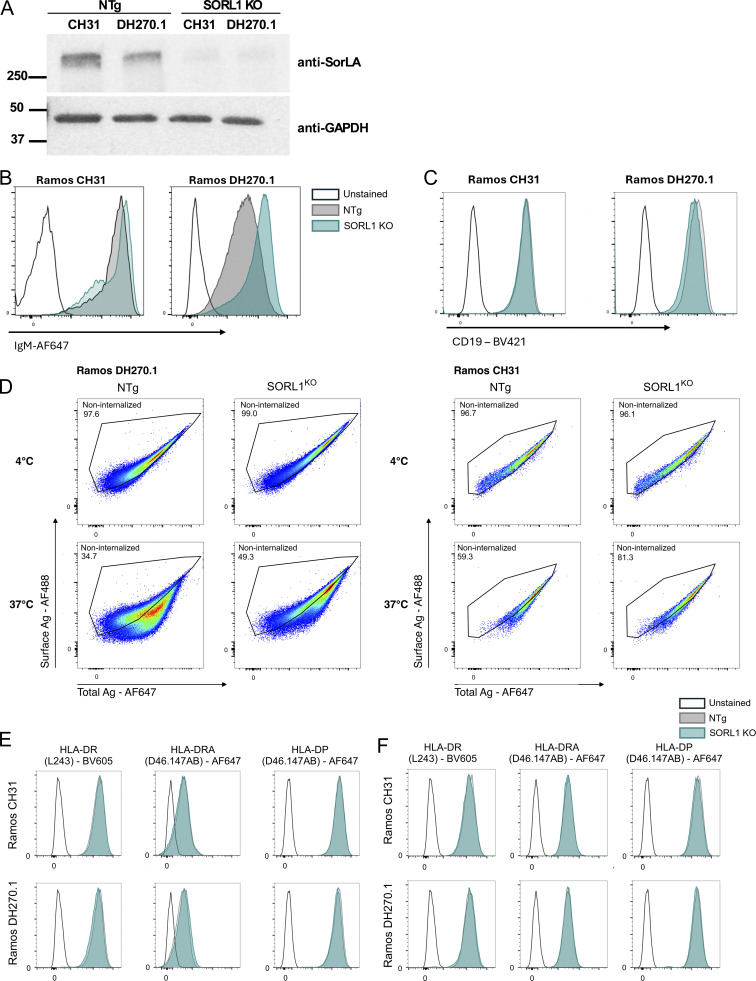
**Generation and validation of *SORL1*** ^**KO**^ **versions of Ramos B cell lines expressing IgM BCRs with specificities of HIV-1 bnAbs CH31 and DH270.1. (A)** Western blot analysis of SorLA protein expression in *SORL1*^KO^ and NTg control Ramos CH31 and Ramos DH270.1 HIV-1 bnAb IgM BCR-expressing B cell lines. **(B)** Surface IgM staining of the same cell lines. **(C)** Surface CD19 staining of the same cell lines. Ramos CH31 used as unstained controls. **(D)** Antigen internalization assay data from *SORL1*^KO^ and NTg control lines. Cells were incubated with a surrogate antigen (fluorescent, biotinylated anti-IgM F(ab′)_2_) (total antigen) and subsequently stained with fluorescent streptavidin (surface antigen). Flow cytometry dotplots show data from cells incubated at 4°C to prevent BCR endocytosis and cells incubated at 37°C. **(E and F)** (E) Surface staining and (F) intracellular staining of *SORL1*^KO^ and NTg control Ramos CH31 and Ramos DH270.1 lines with antibodies that recognize mature, peptide-loaded HLA-DR (L243), or monomorphic epitopes in the C-terminal domain of the HLA-DRα chain (DA6.147) or HLA–DP (B7/21). Ramos CH31 used as unstained controls. Data in all panels are representative of results obtained in at least two independent experiments. Source data are available for this figure: SourceData FS4.

To advance on this finding and gain insight into effects on the repertoire of peptides generated following processing of BCR-bound antigens in addition to levels of peptide–MHC II presentation, we generated *SORL1*^KO^ (and NTg) versions of two Ramos-Cas9 cell lines expressing IgM BCRs with specificities of HIV-1 broadly neutralizing antibodies (bnAbs) targeting different sites on the viral envelope (Env) glycoprotein: the CD4-binding site (CH31) (Bonsignori et al., 2012; Hossain et al., 2022) and V3 glycan site (DH270.1) (Bonsignori et al., 2017; Saunders et al., 2019) Fig. S4 A). Initial characterization indicated slightly higher levels of surface IgM (but similar surface CD19 expression) (Fig. S4, B and C) and reduced antigen internalization (Fig. S4 D) in these *SORL1*^KO^ lines compared with NTg versions, paralleling the phenotype of previously generated Ramos-Cas9 *SORL*^KO^ lines and *Sorl1*^−/−^ primary murine B cells (Figs. 2 and 3). No differences were apparent in total cellular levels of HLA-DR and HLA–DP (assessed by staining with antibody clones DA6.147 and B7/21, antibodies that recognize monomorphic epitopes in the C terminus of the HLA-DRα chain or in HLA–DP, respectively), or in cell surface or total levels of mature, peptide-loaded HLA-DR molecules (assessed by staining with antibody clone L243, which binds to mature peptide-loaded but not invariant chain-bound HLA-DR (Walseng et al., 2008), in *SORL1*^KO^ lines versus NTg versions (Fig. S4, E and F).

To evaluate HLA II presentation of antigen internalized via the BCR, cells were incubated with soluble, stabilized gp140 SOSIP HIV-1 Env trimers (SOSIPs) recognized by the CH31 (CH505TFv4.1 SOSIP) or DH270.1 (CH848 10.17DT SOSIP) HIV-1 bnAbs, which engage the BCRs of and trigger calcium flux in CH31 and DH270.1 IgM-expressing Ramos cell lines, respectively (Hossain et al., 2022; Saunders et al., 2019). After allowing 24 h for cells to internalize the Env trimers and process and present Env-derived peptides, cells were lysed, HLA II immunoprecipitated, and HLA II–bound peptides were isolated and analyzed by mass spectrometry. Multiple nested sets of Env peptides sharing a common HLA II–binding core with varying N- and C-terminal overhangs were detected in the immunopeptidome, with no evidence of substantial differences in the repertoire of peptides presented by NTg and *SORL1*^KO^ Ramos-Cas9 lines (Fig. 6 C). Use of label-free quantification to compare the relative abundance of Env-derived peptides presented by *SORL1*^KO^ cells and NTg cells revealed that Env-derived peptides were, on average, less abundant in the immunopeptidome of *SORL1*^KO^ cells than that of NTg cells, with the difference reaching statistical significance in the Ramos-Cas9 CH31 dataset (Fig. 6 D). These results indicate that the reduction observed in BCR-mediated antigen internalization and degradation in SorLA-deficient B cells has a subsequent impact on levels of antigen-derived peptide–MHC II presentation, which may have a detrimental effect on humoral immune responses by reducing the ability of B cells to attract cognate help from CD4^+^ T cells (Schwickert et al., 2011).

### SorLA contributes to humoral immune responses *in vivo*

To investigate how reduced antigen presentation influences B cell–mediated immune responses *in vivo*, we immunized *Sorl1*^−/−^ mice with a T-dependent antigen, NP-CGG adjuvanted with alum by subcutaneous injection into each leg flank to target inguinal draining lymph nodes.

**Figure 7.**
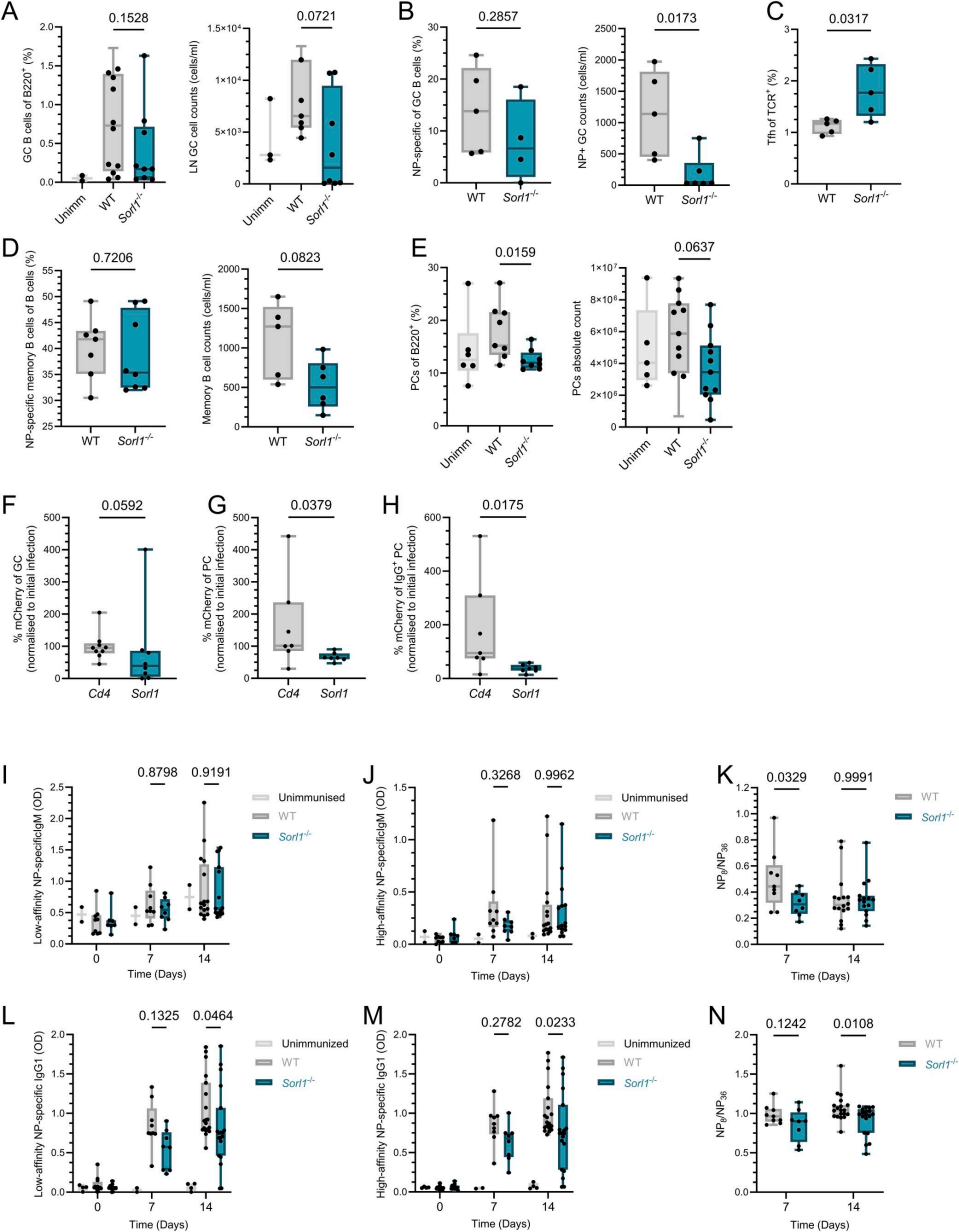
**SorLA contributes to humoral immune responses *in vivo*. (A)** Percentage and absolute counts of lymph node GC B cell populations 14 days following immunization with NP-CGG in WT, *Sorl1*^−/−^, and unimmunized WT mice. P value shown calculated by Mann–Whitney U test, *n* = 9–12 mice from three experiments. **(B)** Percentage and absolute counts of lymph node NP-specific GC B cell populations 14 days following immunization with NP-CGG in WT, *Sorl1*^−/−^, and unimmunized WT mice. *n* = 4–5 mice from two experiments. **(C)** Lymph node Tfh cell populations 14 days following immunization with NP-CGG in WT, *Sorl1*^−/−^, and unimmunized WT mice. *n* = 5 mice from one experiment. **(D)** Percentage and absolute counts of lymph node memory B cell populations 14 days following immunization with NP-CGG in WT, *Sorl1*^−/−^, and unimmunized WT mice. *n* = 7–8 mice from two experiments. **(E)** Percentage and absolute counts of lymph node plasma cell populations 14 days following immunization with NP-CGG in WT, *Sorl1*^−/−^, and unimmunized WT mice. *n* = 8–10 mice from two experiments. **(F)** Development of GC cell populations in spleen of *Cd4*- or *Sorl1*-targeted bone marrow chimeras at 14 days after immunization. % mCherry^+^ cells is normalized to the initial infection (% mCherry^+^) of HSC for each sgRNA per experiment. *n* = 7 mice from two independent experiments. **(G)** Development of plasma cell populations in spleen of *Cd4*- or *Sorl1*-targeted bone marrow chimeras at 14 days after immunization. % mCherry^+^ cells is normalized to the initial infection (% mCherry^+^) of HSC for each sgRNA per experiment. *n* = 7 mice from two independent experiments. **(H)** mCherry expression in class-switched IgG^+^ plasma cell populations in spleen of *Cd4*- or *Sorl1*-targeted bone marrow chimeras at 14 days after immunization. % mCherry^+^ cells is normalized to the initial infection (% mCherry^+^) of HSC for each sgRNA per experiment. *n* = 7 mice from two independent experiments. **(I)** Low-affinity NP-specific IgM antibody production, quantified by NP36-binding ELISA. *n* = 19–20 mice from four independent experiments. **(J)** High-affinity NP-specific IgM antibody production, quantified by NP(8) binding ELISA. *n* = 19–20 mice from four independent experiments. **(K)** Ratio of IgM binding to NP(8) and NP(36). *n* = 19–20 mice from four independent experiments. **(L)** Low-affinity NP-specific IgG1 antibody production, quantified by NP36-binding ELISA. *n* = 19–20 mice from four independent experiments. **(M)** High-affinity NP-specific IgG1 antibody production, quantified by NP(8)-binding ELISA. *n* = 19–20 mice from four independent experiments. **(N)** Ratio of IgG1 binding to NP(8) and NP(36). *n* = 19–20 mice from four independent experiments. Data are shown as box and whisker plots, where the line indicates the median, the box represents the interquartile range, and whiskers denote the minimum and maximum values. P values shown in A–H are calculated by Mann–Whitney U test. P values shown in I–N are calculated by two-way ANOVA with multiple comparisons.

At 14 days after immunization, total GC B cell numbers were increased in immunized mice, but comparable between *Sorl1*^−/−^ and WT mice (Fig. 7 A). NP-specific GC B cells were slightly reduced as a proportion of GC B cells in *Sorl1*^−/−^ mice and significantly decreased in absolute number (Fig. 7 B). Interestingly, Tfh cell populations, identified as TCR^+^CD4^+^CD44^+^CXCR5^+^, were increased in *Sorl1*^−/−^ mice, suggesting that there may be some compensatory mechanisms to boost the Tfh response (Fig. 7 C). NP-specific memory B cell frequency was similar between *Sorl1*^−/−^ and WT mice as a proportion of total B cells and modestly reduced in absolute number in the absence of SorLA (Fig. 7 D). In contrast, plasma cell frequencies in inguinal lymph nodes were significantly decreased with *Sorl1* deletion (Fig. 7 E), suggesting SorLA may play a greater role in GC selection or PC differentiation.

To directly test the B cell–intrinsic role of SorLA in antigen-specific responses, we immunized Cd4- and Sorl1-targeting SW_HEL_-Cas9 bone marrow chimera mice with sheep RBCs (SRBCs) conjugated to hen egg lysozyme (HEL). At 14 days after immunization, *Sorl1*-targeted mCherry^+^ populations were reduced in the GC (Fig. 7 F), PC (Fig. 7 G), and IgG^+^ PC (Fig. 7 H) compartments, compared with *Cd4*-targeted cells. Although we cannot exclude a role for SorLA in other lineages, these data support a B cell–intrinsic role in GC and PC responses.

**Figure S5.**
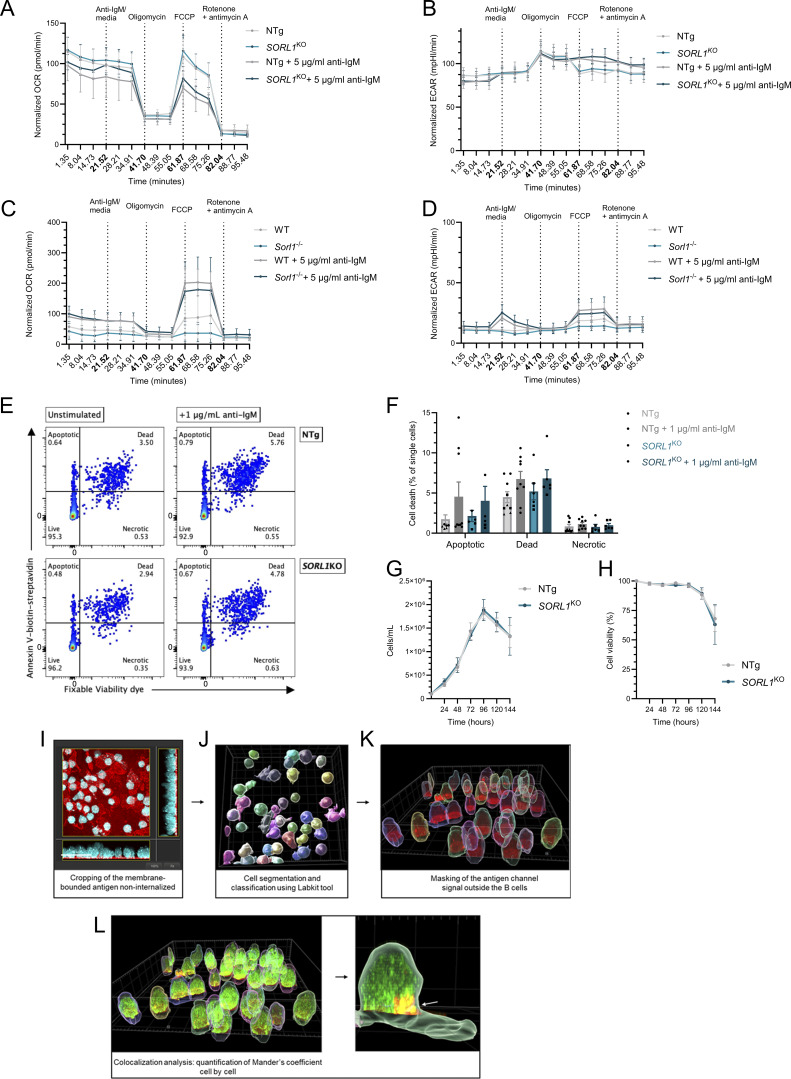
**SorLA does not regulate Ramos-Cas9 B cell metabolism. (A)** Adapted Seahorse XF Cell Mito Stress Test. OCR, normalized to DAPI MFI per well for NTg and *SORL1*^KO^ Ramos-Cas9 B cells. *n* = 2–3 with three technical replicates each. **(B)** Adapted Seahorse XF Cell Mito Stress Test. ECAR, normalized to DAPI MFI per well for NTg and *SORL1*^KO^ Ramos-Cas9 B cells. *n* = 2–3 with three technical replicates each. **(C)** Adapted Seahorse XF Cell Mito Stress Test. OCR, normalized to DAPI MFI per well for WT and *Sorl1*^*−/−*^ mice B cells. *n* = 3 with three technical replicates each. **(D)** Adapted Seahorse XF Cell Mito Stress Test. ECAR, normalized to DAPI MFI per well for WT and *Sorl1*^*−/−*^ mice B cells. *n* = 3 with three technical replicates each. **(E)** Gating strategy for live, apoptotic, dead, and necrotic cells using Annexin V-biotin-streptavidin eFluor 450 and fixable viability dye eFluor 780. Q1: apoptotic, Q2: dead, Q3: necrotic, and Q4: live. **(F)** Apoptotic, dead, and necrotic cells at 48-h after treatment with or without 1 μg/ml anti-IgM stimulation. *n* = 8–12 independent Ramos-Cas9 transductions with three NTg sgRNAs and two SORL1-targeting sgRNAs from four independent experiments. **(G)** Proliferation in NTg and *SORL1*^KO^ Ramos-Cas9 B cell populations over 144 h *n* = 4–6, from two independent experiments. **(H)** % Cell viability in NTg and *SORL1*^KO^ Ramos-Cas9 B cell populations over 144 h *n* = 4–6, from two independent experiments. **(I)** Cropping of the membrane-bounded antigen (red) non-internalized by SorLA-EGFP–expressing Ramos B cells or *SORL1*^KO^ or NTg Ramos-Cas9 B cells (blue). **(J)** Cell segmentation and classification using the Labkit machine learning tool in IMARIS software. **(K)** Masking of the antigen channel signal (red) and vesicle marker channel signals (Rab5, Rab7, Rab11, Lamp1, or MHC II) to include staining inside masked B cells. **(L)** Quantification of thresholded Mander’s coefficient cell by cell (colocalization analysis). Gray arrow indicates colocalized antigens (red) with SorLA proteins (green), which are shown as yellow dots. Data in A–D and F–H show mean ± SEM.

As GC B cell metabolism and apoptosis is crucial for GC output *in vivo* (Victora and Nussenzweig, 2022), we investigated Ramos- Cas9 B cell metabolism, apoptosis, and proliferation to understand if SorLA contributes to these cellular processes. Seahorse XF Cell Mito Stress Test revealed similar oxygen consumption rate (OCR) and extracellular acidification rate (ECAR) profiles between *SORL1*^KO^ and NTg Ramos-Cas9 B cells with and without anti-IgM stimulation (Fig. S5, A and B), suggesting no significant differences in metabolic capacity. Treatment of Ramos B cells with anti-IgM without T cell help leads to downregulation of proteins involved in B cell survival and proliferation, as well as the activation of caspase-3 and DNA damage, resulting in apoptotic cell death (Hui et al., 2022). Analysis revealed comparable anti-IgM–induced apoptosis between *SORL1*^KO^ and NTg Ramos-Cas9 B cells (Fig. S5 D), suggesting similar propensity to undergo apoptosis. In agreement with these data, *SORL1*^KO^ Ramos-Cas9 B cells proliferate and undergo apoptosis similarly to NTg cells in culture (Fig. S5, E and F).

Finally, we sought to understand whether the decreased plasma cell output following *Sorl1* deletion translated into decreased specific antibody production and affinity maturation. We quantified NP-specific serum antibodies in *Sorl1*^−/−^ and WT mice preimmunization, and at 7 and 14 day after immunization with NP-CGG, NP(36)-BSA and NP(8)-BSA were used to quantify low- and high-affinity NP-specific serum antibodies, respectively, with the NP(8)/NP(36) ratio used to measure affinity maturation between 7 and 14 days after immunization.

Levels of low-affinity NP-specific IgM antibodies were similar between *Sorl1*^−/−^ and WT mice (Fig. 7 I); however, levels of high-affinity NP-specific IgM antibodies were slightly reduced in *Sorl1*^−/−^ mice at 7-day after immunization (Fig. 7 J). This was reflected by reduced early IgM affinity maturation in *Sorl1*^−/−^ mice (Fig. 7 K).

Levels of both low and high-affinity NP-specific IgG1 antibodies were reduced in *Sorl1*^−/−^ mice early in the response and significantly reduced at 14 day after immunization (Fig. 7, L and M). Again, this was reflected by significantly delayed IgG1 affinity maturation in *Sorl1*^−/−^ mice at 14 day after immunization (Fig. 7 N).

## Discussion

In this study, we identify SorLA as a novel regulator of humoral immune responses and demonstrate its role in BCR–antigen uptake, trafficking, and antigen presentation.

A proportion of SorLA molecules are expressed at the B cell surface and interact with the BCR, regulating uptake of both soluble and membrane-bound antigens. This surface expression is in line with previous reports in other cells (Jacobsen et al., 1996), including myeloid and B cell malignancies, where SorLA levels are elevated (Sakai et al., 2012). While direct interaction with the BCR has not been described previously, proteomic analysis of the BCR signalosome revealed that many SorLA-interacting proteins (e.g., GGA2, VPS26, PACS1, and PPP1CA) undergo posttranslational modifications following BCR activation (Satpathy et al., 2015), strongly implicating this intracellular trafficking pathway in B cell processes following BCR engagement. Additionally, the Immunological Proteome Resource shows SorLA is predominantly expressed in follicular B cells, and expression increases upon BCR and CD40L stimulation (Brenes et al., 2023). These studies support a role for SorLA in cellular processes occurring after B cell activation. SorLA deletion resulted in increased surface BCR levels but did not significantly alter the BCR signalling cascade. This may be due to the small biological effect (around 10% change in surface BCR), which may not produce detectable differences in signalling, or individual surface BCR molecules in the *SORL1*-deleted cells exhibiting altered recruitment or association with signalling machinery.

As both soluble antigen uptake and extraction from PMS are affected by *SORL1* deletion, the defect is likely not due to differences in contractile strength at the B cell synapse. SorLA’s role in antigen uptake from PMS suggests it may play a role in endosome formation or antigen packaging following BCR stimulation, perhaps through membrane organization of the BCR, or SorLA’s ability to bind clathrin adaptor AP-2 required for CME (Nielsen et al., 2007).

Here, we reveal that SorLA localizes with endolysosomal compartments following B cell antigen uptake, regulating BCR–antigen trafficking. This is in agreement with SorLA’s prominent role in intracellular cargo trafficking through endosomal recycling and lysosomal degradation pathways of various ligands, such as amyloid precursor protein, human epidermal growth factor receptor, and glutamate receptor 1 (Andersen et al., 2005; Mishra et al., 2022; Pietilä et al., 2019; Schmidt et al., 2017). We demonstrate that downstream BCR–antigen trafficking is not entirely abolished but appears significantly reduced and delayed; this is associated with reduced lysosomal processing quantified as anti-IgM–induced BCR degradation. The reduced accumulation of antigen within LAMP1^+^ compartments may reflect altered intracellular routing, delayed trafficking kinetics, or a combination of both; the current data do not distinguish between these possibilities. Although the exact protein–protein interactions remain to be elucidated, our first report points to a role for SorLA in both antigen–BCR endocytosis and downstream degradation.

A role for SorLA in lysosome maintenance has been suggested previously, as SorLA-silenced HER2-dependent cancer cells and microglia exhibited enlarged, dysfunctional lysosomes (Mishra et al., 2024; Pietilä et al., 2019). To investigate whether the observed reduction in BCR degradation in *SORL1*^KO^ Ramos-Cas9 cell lines is attributed to lysosome dysfunction, we conducted a comprehensive characterization of lysosomes, through quantification of lysosome-associated protein expression, and employing LysoTracker imaging with 3D object analysis. Our analyses revealed comparable lysosome numbers, structure, and acidification between *SORL1*^KO^ and NTg Ramos-Cas9 cells. SorLA does not seem to play a role in B cell lysosome maintenance, highlighting distinct lysosomal functions in different cells.

BCR–antigen degradation is a critical step for generating antigenic peptides. This occurs in late endosomal/lysosomal compartments to which trafficking of recently synthesized invariant chain-bound MHC II molecules is promoted following BCR signalling, enabling loading of antigen-derived peptides onto MHC II for subsequent presentation (Lanzavecchia, 1985; Roche and Furuta, 2015; Yuseff et al., 2013). SorLA-deficient B cells did not exhibit defects in BCR signalling, so although a role for SorLA in MHC II trafficking cannot be excluded, the reduced BCR-mediated antigen internalization and altered downstream trafficking in these cells likely explain the reduced Eα presentation we observed in *Sorl1*^−/−^ primary cells *in vitro* and relatively lower abundance of HIV-1 Env protein–derived peptides presented by *SORL1*^KO^ versus control HIV-1 bnAb BCR-expressing Ramos-Cas9 cells. While overall levels of BCR-internalized antigen-derived peptide presentation were reduced, we did not observe differences in the repertoire of Env-derived peptides presented in *SORL1*^KO^ versus control B cells, providing further evidence for normal lysosomal function in *SORL1*^KO^ cells.

Presentation of antigen-derived peptides with MHC II is critical to enable B cells to attract help from cognate CD4^+^ T cells. Functional changes in T cell activation were not directly demonstrated here but are inferred from antigen presentation data. While B cell development was not perceptibly altered in *Sorl1*^*−/−*^ mice, humoral immunity was impacted. In this setting, where all B cells lacked expression of SorLA, antigen-specific IgM production was unimpaired, indicative of efficient initial B cell activation and plasmablast differentiation. However, class-switched antibody production was reduced, and although GCs were formed and antigen-specific memory B cells, which differentiate from GC B cells of a range of affinities (Viant et al., 2020), were produced, overall levels of plasma cell generation were diminished, and antibody affinity maturation was impaired. Together, these observations highlight reductions in processes dependent on CD4^+^ T cell help, as early encounters between CD4^+^ T cells and B cells support B cell class switching (Roco et al., 2019), and CD4^+^ T cells play a crucial role in B cell “refueling” to undergo efficient affinity maturation in GCs (Long et al., 2022).

While SorLA does not play a clear role in B cell development *in vivo*, it may play a role in development of other immune cells, such as CD8^+^ T cells. This is supported by SorLA’s expression in CD8^+^ T cells, but not other T cell subsets (Brenes et al., 2023). The role of SorLA in other immune compartments warrants further investigation.

The link between mutations in *SORL1* and Alzheimer’s disease is well-established (Fjorback et al., 2012; Jensen et al., 2023). Furthermore, studies demonstrate a link between *SORL1* mutations and Parkinson’s disease pathogenesis, likely through SorLA’s interaction with LRP10 (Grochowska et al., 2021; Wang et al., 2022; Xiromerisiou et al., 2021). Here, we present the first account of the critical role of SorLA in immunity. Interestingly, recent studies highlight significantly elevated risk of SARS-CoV-2 infection and subsequent hospitalization both before and after vaccination in Parkinson’s disease patients (Hippisley-Cox et al., 2021; Huang et al., 2023). Altered antigen presentation and reduced humoral responses reported here, may be one contributing factor to clinical observations in these patients. This work opens further research questions around novel immune regulation pathways related to intracellular trafficking; molecular mechanisms warrant further investigation to define any potential for therapeutic intervention.

Together, our results provide evidence of a novel role for SorLA in BCR–antigen trafficking and in modulation of immune responses *in vivo*. This study paves the way for critical investigations into immune regulation through intracellular trafficking and the role of soluble compared with surface-bound SorLA.

## Materials and methods

### Mice and cell isolation

C57BL/6, SW_HEL_-Cas9 (Brink et al., 2015; Malinova et al., 2021), and *Sorl1*^−/−^ (Andersen et al., 2005) mice on a C57BL/6 background were used as a source of primary B cells. *Sorl1*^−/−^ mice were bred as a homozygous line; age- and sex-matched WT C57BL/6 (Charles River) controls were housed identically, including joint handling and brief cohousing where possible to minimize environmental and microbiota effects. To generate bone marrow chimeras, gene-disrupted HSCs from SW_HEL_-Cas9 mice were adoptively transferred into recipient HSC-ablated C57BL/6 mice (RRID:IMSR_JAX:000664) by intravenous injection (100,000 cells/host). Immune cell ablation was achieved by either double-dose lethal whole-body irradiation (2 × 5 Gy) or triple-dose busulfan treatment by (25 mg/kg intraperitoneal injection). Cage water was supplemented with Baytril. 8 wk was allowed for immune cell reconstitution. For *in vitro* studies, untouched murine B cells were isolated by negative selection using anti-CD43 microbeads (Miltenyi). For bone marrow chimeras, HSCs were isolated from SW_HEL_-Cas9 mice using a lineage cell depletion kit (Miltenyi). All mice were bred and treated in accordance with guidelines set by the UK Home Office (Northern Ireland Department of Health) and following approval of QUB Animal Welfare and Ethics Review Body. Animals of both sexes were used throughout the study. Mice were randomly assigned to different treatments (different sgRNA).

### Cell lines

Suspension cells were maintained in complete RPMI (10% FBS [Gibco], 100 µM nonessential amino acids [Gibco], and 1× antibiotic-antimycotic solution [Sigma-Aldrich]) at 37°C and 5% CO2 upright in T-25, T-75, or T-175 culture flasks. Ramos Burkitt lymphoma cells (RRID: CVCL_0597) were passaged every 2–3 days with a 1:10 split in complete RPMI. Cells were authenticated every 3 years and tested for mycoplasma contamination every 6–8 wk.

HEK293T cell monolayers were grown and maintained in complete DMEM (10% FBS, 100 µM nonessential amino acids, and 1× antibiotic-antimycotic solution) at 37°C and 5% CO2 in T-75 or T-175 culture flasks. When 90% confluent, cells were washed with 1× PBS and incubated with 0.05% trypsin-EDTA (Gibco) for 5 min at 37°C detach cells. Cells were split 1:10 in complete DMEM.

### CRISPR-Cas9–mediated deletion of *SORL1*/*Sorl1*

Generation of Ramos cells stably expressing Cas9 was previously described (Malinova et al., 2021). The same method was used to stably express Cas9 in Ramos cell lines expressing IgM BCRs with specificities of HIV-1 bnAbs targeting the CD4-binding site (CH31) (Bonsignori et al., 2012; Hossain et al., 2022) or V3 glycan site (DH270.1) (Bonsignori et al., 2017; Saunders et al., 2019).

Human *SORL1* and non-targeting, and murine *Sorl1 and Cd4* sgRNA sequences were designed using the Broad Institute’s sgRNA designer (CRISPick).

Forward and reverse oligonucleotides were synthesized, annealed, and individually cloned into lentiGuide-Puro lentiviral CRISPR plasmid with ampicillin and puromycin resistance cassettes using the Feng Zhang Lab protocol (Shalem et al., 2014).

*SORL1*-targeting sequences:

- *SORL1*_1: 5′-TGG​ACC​TCA​CTA​CTA​CAC​AT-3′
- *SORL1*_3: 5′-GGC​TCC​GAT​GAA​CAG​CAC​TG-3′

Human non-targeting sequences:

- NT_3: 5′-GGT​TAG​AGA​CTA​GGC​GCG​CG-3′
- NT_4: 5′-GAA​TCG​ACC​GAC​ACT​AAT​GT-3′
- NT_5: 5′-ACT​GCG​GAG​CGC​CCA​ATA​TC-3′

*Sorl1*-targeting sequence:

- *Sorl1*_1: 5′-CAC​CGC​ATA​GAA​CCA​TTA​ATC​AGG​G-3′

*Cd4*-targeting sequence:

- *Cd4*_1: 5′-CAC​CGA​CTC​ACC​CTC​AAG​ATA​CCC​C-3′

*SORL1*-, non-, *Sorl1*-, and *Cd4*-targeting lentivirus was produced by co-transfecting HEK293T cells at 80% confluence in a T-175 flask using *Trans*IT-LT1 transfection reagent according to the manufacturer’s instructions, with pMD2.G and psPAX lentiviral Env and packaging plasmids along with the cloned pLentiGuide-Puro plasmids encoding puromycin resistance. Lentivirus was harvested 48 h following transfection, passed through a 0.2-µM filter and either used immediately or stored at −80°C. Lentivirus for HSC transduction was concentrated by cold ultracentrifugation for 120 min at 80,000 *g*. Lentiviral pellets were resuspended in Opti-MEM (Thermo Fisher Scientific), aliquoted, and used immediately or stored at −80°C.

For Ramos-Cas9 B cell transduction, 4 × 10^5^ cells were seeded in 500 μl of 10 μg/ml polybrene complete RPMI and spin infected with 1 ml of lentivirus-containing supernatant for 90 min at 1,350 *g*. This corresponds to approximate multiplicity of infection of 0.5–2, as determined by serial dilution and test infections with fluorophore-encoding lentivirus. 48 h following spinfection, cells were selected using 2.5 μg/ml puromycin for 2 wk. Gene-edited cell lines (2 *SORL1*-deleted, 3 WT) were maintained independently and are denoted by distinct symbols throughout the manuscript.

For HSC transduction, lineage-negative HSCs were cultured in StemSpan SFEM supplemented with 1% FBS, 1× antibiotic-antimycotic solution, 100 ng/ml mSCF, 100 ng/ml mFLT3L, and 20 ng/ml hTPO, then infected twice, 3 h apart, with lentivirus encoding mouse sgRNA and mCherry.

### SorLA-EGFP–expressing Ramos B cell line

The SorLA-EGFP expression construct was generated as previously described (Spoelgen et al., 2006). SorLA-EGFP lentivirus was produced as described above for pLentiGuide_puro.

### Antigens

Surrogate antigens used in solution or on PMS were F(ab′)_2_ fragment goat anti-human IgM Fc5µ fragment (Jackson ImmunoResearch) for Ramos cells and F(ab′)_2_ fragment goat anti-mouse IgM µ chain (Jackson ImmunoResearch) for murine splenic B cells). Antigens were biotinylated using EZ-Link Sulfo-NHS-Biotin No-Weigh Format (Thermo Fisher Scientific) and conjugated to Atto 647N NHS ester (Merck) or Atto 550 NHS ester (Merck) in sodium carbonate buffer, according to the manufacturer’s instructions. Excess dye was removed using Zeba Spin Desalting Columns (Thermo Fisher Scientific). Human holo-transferrin (Sigma-Aldrich) was also fluorescently labelled and biotinylated for transferrin internalization assays.

Soluble, stabilized gp140 SOSIP HIV-1 Env trimers (SOSIPs) recognized by the CH31 (CH505TFv4.1 SOSIP) and DH270.1 (CH848 10.17DT SOSIP) HIV-1 bnAbs that engage the BCRs of DH270.1 and CH31 IgM-expressing Ramos B cell lines, respectively, were produced as previously described (Hossain et al., 2022; Saunders et al., 2019).

### Soluble internalization assay

1 × 10^6^ cells were incubated with fluorescently labelled, biotinylated surrogate antigen or transferrin described above at 5 µg/ml and a Fixable Viability Dye eF780 marker in HBSS for 15 min on ice. Cell suspensions were washed with cold 1× PBS, split into two separate samples, and resuspended in either 37°C or ice cold HBSS and incubated at respective temperatures for 15 min 16% formaldehyde was added to a final concentration of 4% to fix the cells, and remaining surface surrogate antigen/transferrin was counterstained with Streptavidin eFluor 450 (Invitrogen) at 1:400 dilution.

For BCR recycling assay, cells were stimulated with 5 µg/ml biotinylated anti-IgM Fab or F(ab′)_2_ for 15 min at 37°C to allow binding and internalization. BCR remaining on the cell surface after anti-IgM stimulation was counterstained with excess streptavidin FITC for 30 min on ice. Cells were incubated again at 37°C to allow recycling or intracellular trafficking. Cells were fixed (4% formaldehyde) and recycled BCR–anti-IgM was counterstained with streptavidin eFluor 450. Samples were analyzed on a BD FACSCanto II, gating on live cells (Fixable Viability Dye eF780). A sample stained with streptavidin eF450 alone was used for background subtraction in FlowJo 10.

### Membrane-bound antigen internalization assay, endolysosomal localization, and LysoTracker analysis

PMS were generated as described previously (Nowosad and Tolar, 2017). Antigen-coating density was standardized within each experiment by measuring the MFI of the antigen channel and ensuring comparable levels across all wells in the experiment.

Ramos-Cas9 B cell lines were added to prewarmed imaging wells to interact with PMS at 37°C. Cells were fixed in 4% paraformaldehyde (PFA) after 15 min. Z-stacks were covering the PMS, and attached cells were acquired by epifluorescence, and antigen extraction from PMS was analyzed and quantified using the Analyze Internalization GUI, an image analysis tool to measure the amount of ligand that cells extract and internalize from planar substrates (Nowosad and Tolar, 2017). It uses fast bandpass filtering to detect fluorescent antigen clusters in 3D and quantifies their fluorescence using a local background subtraction to reduce the effect of light scattering from PMS.

For endolysosomal localization, fixed cells on PMS were permeabilized with 0.1% Triton X-100 (Thermo Fisher Scientific) for 5 min, then washed and blocked with 2.5% BSA. Primary antibody incubations were carried out at 4°C overnight. Fluorescently tagged secondary antibodies were incubated for 1 h, and then wells were imaged by confocal microscopy. Colocalization analysis was conducted on Imaris (Oxford Instruments). Image processing consisted of: (1) cropping the z-stack to remove the PMS-bound antigen (not internalized); (2) cell segmentation and classification using the machine learning segmentation tool trained on the surface marker channel; (3) masking of the fluorescent surrogate antigen and proteins of interest (Rab5, Rab7, Rab11, Lamp1, and MHC II) channels within segmented cells. To calculate voxel-based colocalization coefficients, thresholds were applied to each channel, standardized across conditions, and checked visually in Imaris and ImageJ. Finally, thresholded Mander’s coefficients were calculated per cell. The following thresholds are an example of one representative experiment: 647 channel (surrogate antigen) = 822; 488 channel (organelle markers) = 1,151; and 594 channel (organelle markers) = 1,163. Image processing and colocalization analysis pipeline are shown in Fig. S5.

For lysosomal analysis, cells were stained with LysoTracker Red DND-99 (Thermo Fisher Scientific) according to the manufacturer’s instructions at 37°C, washed, then seeded into a poly-L-lysine–treated 8-well chamber and imaged live by confocal microscopy.

Epifluorescence was carried out on a Leica TIRF MultiColor system with a DMi8 inverted microscope using a metal-halide lamp as a light source. Emitted photons were collected with the Digital Camera Andor Zyla 4.2 Megapixel sCMOS VSC03616. The HC PL APO 100×/1.47 OIL CORR TIRF was used for image acquisition. Appropriate filter cubes were used for the emitted fluorescence. Leica Application Suite X (LAS X) 3.9.0.28093 was used for image acquisition and processing (Leica Microsystems).

Confocal microscopy was carried out by laser confocal microscopy using Leica STELLARIS TCS microscope. Samples were excited with appropriate laser lines, and the emitted photons were collected via HyD GaAsP spectral detectors between 420 nm and 460 nm, 500 nm and 530 nm, and 570 and 620 nm as appropriate. The HC PL APO 100×/1,40 OIL STED was used for image acquisition. Fluorescence images were collected at a 16-bit depth 1,024 × 1,024-pixel resolution format, and a scanner speed of 600 Hz. LAS X 4.7.0.28176 was used for image acquisition and processing.

### Antibodies and flow cytometry

Erythrocyte-lysed single-cell suspensions were blocked with anti-CD16/32 for 15 min and stained with appropriate antibodies for 30 min on ice. The following stains and antibodies were used for murine B cell immunophenotyping: B220-BUV737 (RA3-6B2), CD93-PerCP-Cy5.5 (AA4.1), CD23-PE-Cy7 (B3B4), CD21/35-ef450 (4E3), IgM-APC (II/41), c-KIT-PE-Cy7 (2B8), CD25-PerCP-Cy5.5 (PC61), CD19-BV605 (1D3), and IgD-ef450 (11–26).

In spleen, follicular and marginal zone B cells were detected based on CD23 and CD21/35 expression in the mature B cell population (B220^+^CD93^−^). Transitional populations were detected in the immature population (B220^+^CD93^+^) based on expression of CD23 and IgM. In the bone marrow, early B cell populations were defined as follows: pre-pro-B (B220^+^c-KIT^+^CD25^−^CD19^−^), pro-B (B220^+^c-KIT^+^CD25^−^CD19^+^), and pre-B cells (B220^+^c-KIT^+^CD25^+^CD19^+^). Immature and recirculating populations were detected based on expression of IgM and IgD.

Immunophenotyping samples were acquired on a BD FACSSymphony and analyzed on FlowJo. Immunophenotyping gating strategies are shown in Fig. S4.

For analysis of surface IgM BCR expression by HIV bnAb BCR-expressing Ramos lines, 0.5 × 10^6^ cells were stained with LIVE/DEAD Fixable Aqua Dead Cell Stain Kit (Thermo Fisher Scientific) and Alexa Fluor 647–conjugated anti-human IgM antibody (BioLegend, Clone MHM-88) at 1:500 dilution in PBS for 15 min. Cells were then washed once with PBS, fixed with 2% PFA in PBS for 15 min, washed, and resuspended in 200 μl of PBS for flow cytometric analysis.

To analyze surface CD19 and HLA-II expression, 0.5 × 10^6^ cells were first incubated with TruStain FcX (BioLegend) diluted 1:25 in PBS for 10 min at room temperature (RT) to block nonspecific Fc-mediated interactions. Cells were then stained with LIVE/DEAD Fixable Aqua Dead Cell Stain Kit (1:500 dilution; Thermo Fisher), Brilliant Violet 421–conjugated anti-human CD19 antibody (1:100 dilution; Clone HIB19; BioLegend), BV605-conjugated anti-HLA-DR antibody (clone L243; BioLegend, 1:250 dilution), and BV711-conjugated anti-HLA–DP antibody (1:200 dilution; Clone B7/21; BD Biosciences) for 15 min. Cells were washed, fixed, washed again, and resuspended for flow cytometry as described for surface IgM analysis.

For intracellular staining, 0.5 × 10^6^ cells underwent Fc block and viability staining as described above. Cell were then washed and fixed using BD Cytofix Fixation Buffer (cat. no. 554655; BD Biosciences) for 15 min at RT, washed with 1× Intracellular Staining Permeabilization Wash Buffer (BD Biosciences), and resuspended in an antibody cocktail prepared in the same buffer: BV605-conjugated anti-HLA-DR (clone L243, 1:250 dilution), Alexa Fluor 674–conjugated anti-HLA-DRα-chain (clone D46-147; Santa Cruz Biotechnology, 1:50 dilution), and BV711-conjugated anti-HLA–DP (1:200 dilution) antibodies. Following incubation in this antibody cocktail for 15 min at RT, cells were washed once with permeabilization wash buffer then PBS and resuspended for flow cytometry analysis.

Samples were acquired using a BD LSRFortessa X-20 flow cytometer, and data were analyzed using FlowJo software version 10.1.

### Immunoblot, immunoprecipitation, and BCR degradation assay

For immunoblot, a minimum of 5 × 10^6^ cells were lysed in RIPA buffer (Sigma-Aldrich) containing cOmplete EDTA-free protease inhibitor cocktail (Roche) for 10 min on ice, and then cell debris was pelleted by cold centrifugation at 23,000 *g* for 10 minutes at 4°C. Samples were boiled in 1× NuPAGE LDS Sample Buffer (Invitrogen) and 1× NuPAGE Sample Reducing Buffer (Invitrogen) at 95°C for 5 min and then separated by SDS-PAGE using NuPAGE 4–12% Bis-Tris gels (Invitrogen). Proteins were transferred to a PVDF membrane and blocked for 1 h in 5% milk PBST or Intercept (PBS) Blocking Buffer (Li-Cor). Primary antibodies (mouse anti-human SorLA [BD Biosciences, diluted 1:500], mouse anti-human/mouse LR11 [BD Bioscience, diluted 1:500], mouse anti-human/mouse GAPDH [Proteintech; or Invitrogen, diluted 1:4,000], rabbit anti-human LAMP1 [Abcam, diluted 1:500], and goat anti-human cathepsin S [R&D Systems, diluted 1:500]) were incubated overnight in the same blocking solution. Fluorescently tagged secondary antibodies (goat anti-mouse IgG IRDye-680RD [LiCor, diluted 1:15,000], donkey anti-mouse IgG Alexa Fluor 647, goat anti-rabbit IgG Dylight 488, or chicken anti-goat IgG Alexa Fluor 647 [Invitrogen, diluted 1:2,000]) were incubated for 1 h. Membranes were imaged using a ChemiDoc MP Imaging system (Bio-Rad) or G:BOX imaging system (Syngene).

For immunoprecipitation, cell lysates from a minimum of 1 × 10^7^ cells were incubated with biotinylated anti-IgM F(ab′)_2_ or biotinylated anti-CD43 as isotype control and Dynabeads M-280 Streptavidin (Thermo Fisher Scientific) for 4 h at 4°C. Beads were washed in RIPA buffer on a Dynabead magnet three times and eluted in 1× NuPAGE Sample Reducing Buffer at 95°C. Samples were processed for immunoblot as above, and membranes were incubated overnight with mouse anti-human/mouse LR11 overnight in blocking solution. Donkey anti-mouse IgG Alexa Fluor 647 (A31571; Invitrogen) secondary antibody was used for detection.

For BCR degradation assay, 1 × 10^7^ cells/time point were taken, washed three times with 20 ml cold 1× PBS and resuspended in cold 1× PBS at 2.5 × 10^7^ cells/ml. Cells were surface biotinylated with the addition of 200 μl 10 mM biotin/ml (EZ-Link Sulfo-NHS-Biotin No-Weigh Format, Thermo Fisher Scientific) cell suspension on ice for 30 min. Excess biotin was quenched with three washes of 20 ml of 100 mM cold 1× PBS-glycine. Cells were then resuspended in 500 μl 10 µg/ml anti-IgM F(ab′)_2_ RPMI and incubated on ice for 15 min. Cells were then washed twice with cold 1× PBS and resuspended in 3 ml RPMI/time point and then incubated at 37°C and 5% CO_2_.

At each time point (0, 3, 6, 12, and 24 h) cells aliquots were taken, spun down, and lysed with RIPA lysis buffer for 10 min on ice. Cell debris was pelleted by cold centrifugation at 23,000 *g* for 10 min, and supernatants were mixed with 80 μl blocked (2.5% BSA-1 × PBS for 10 minutes at RT) Dynabeads M-280 Streptavidin (Thermo Fisher Scientific) and incubated at 4°C on a daisywheel for 20 h. Streptavidin Dynabeads were magnetically separated and washed, and supernatants were saved. Beads were boiled in sample reducing buffer to elute captured biotinylated proteins. Supernatants and eluates were analyzed by immunoblot as above.

### Eα peptide–MHC II presentation assay

To detect MHC II antigen presentation *in vitro*, primary B cells were incubated with streptavidin conjugated to biotinylated anti-IgM F(ab′)_2_ (final concentration 5 µg/ml) and Eα peptide (biotin-GSGFAKFASFEAQGALANIAVDKA-COOH) at 37°C for 16 h. Samples were then washed and blocked with 2.5% BSA-1 × PBS and Fc block before immunostaining with Y-Ae antibody (anti-Eα:IA^b^), which recognizes Eα_52–68_ bound to IA^b^ MHC II molecules, conjugated to ATTO 647N NHS ester.

### Immunopeptidomics

Two cell lines with distinct BCRs were tested for each NTg and *SORL1*^*KO*^; no technical replicates were performed. Each cell line represents a pooled population containing various sgRNA-directed gene edits rather than single cell clones. 2 × 10^8^ NTg and *SORL1*^KO^ Ramos-Cas9 cells expressing the HIV-1 Env-targeting CH31 (Ramos CH31) or DH270.1 IgM BCRs (Ramos DH270.1) were pulsed with 500 μg CH505TFv4.1 or CH848 10.17DT SOSIP, respectively, in 10 ml AIM-V media supplemented with 2% FBS for 2 h. Cells were then washed once with PBS, resuspended in AIM-V medium with 2% FBS at a final concentration of 1.25 × 10^6^ cells/ml, and cultured for 24 h to allow for antigen internalization, processing, and presentation on MHC II. Following harvesting, cells were washed again with PBS, and cell pellets were frozen at −20°C. Cells were then lysed, peptide–MHCI complexes were cleared by immunoprecipitation with W6/32 antibody, and peptide–HLA-DR and HLA–DP complexes were isolated by serial immunoprecipitation with L243 and B7/21 antibodies, as previously described (Parker et al., 2021). Peptides were eluted using 10% acetic acid and purified by reverse-phase HPLC as previously described (Parker et al., 2021). All fractions were pooled and dried by vacuum centrifugation.

Eluted peptides were dissolved in 20 μl loading buffer (0.1% [vol/vol] trifluoroacetic acid [TFA] and 1% [vol/vol] acetonitrile in water) and analyzed on an Orbitrap Astral mass spectrometer equipped with a Vanquish Neo UHPLC system. Peptides were trapped on a PepMap Neo 5 μm C18 300 μm × 5 mm Trap Cartridge (Thermo Fisher Scientific) and separated on an Aurora Ultimate XT 25 cm × 75 µm column (IonOpticks), packed with 1.7 µm C18 particles at a flow of 200 nl/min. A 60-min gradient from 5 to 50% mobile phase B (80% acetonitrile) in mobile phase A (0.1% FA in water) was applied for peptide elution. Electrospray was set at 1.5 kV. MS1 scans were acquired every 2 s in the Orbitrap at 240,000 resolution, automatic gain control (AGC) target 300%, and maximum injection time of 10 ms in a scan range between 300 and 1,800 m/z. Precursors with charges 2–5 plus were included. Dynamic exclusion was set 20 s with 5 ppm tolerance. Fragmentation was performed on monoisotopic peaks using higher-energy collisional dissociation, with an isolation width of 0.7 m/z. A normalized collision energy of 25% was applied. MS2 spectra were acquired in the Astral analyzer across m/z 120–1,500 at AGC target 30% and maximum injection time of 100 ms.

Raw data files were analyzed in FragPipe version 22 software. Spectral sequence annotation was performed in MSFragger (Teo et al., 2021) against the annotated *Homo sapiens* Swiss-Prot database downloaded on February 2, 2025, supplemented with the sequence of the CH505TFv4.1 (Saunders et al., 2017) or CH848 10.17DT SOSIP antigen (Saunders et al., 2019), respectively. No enzyme specificity was set, and peptide mass error tolerances were set at 5 ppm for precursors and 0.03 Da for MS2 fragments. A 1% false discovery rate was calculated using decoy database search. Label-free relative quantification of peptides was performed using IonQuant (Yu et al., 2021). Subsequent data manipulation and plotting were performed in R. 9-25mer peptides were retained for analysis. SOSIP-derived peptides were aligned to the SOSIP sequence and plotted in a heatmap format, indicating the number of unique peptides detected for each position in the protein. Relative abundance of peptides was calculated using the normalized signal intensities of each peptide and plotted as the log2 of the ratio of intensities of each peptide in the *SORL1*^KO^ versus NTg cells.

### Immunization and serum antibody ELISA

SW_HEL_-Cas9 bone marrow chimera mice were immunized at 14 wk of age (6 wk after ablation and bone marrow reconstitution) by subcutaneous injection of HEL conjugated to SRBCs as previously described (Brink et al., 2015). GC and PC responses were analyzed 14 days after immunization by flow cytometry. *Sorl1*^*−/−*^ and WT mice were immunized at 8–10 wk of age by subcutaneous injection of 50 µg NP-CGG adjuvanted with alum (Thermo Fisher Scientific) into each leg flank to target inguinal draining lymph nodes. Tail vein blood samples were taken before, 7, and 14 day after immunization. Serum NP-specific antibodies were detected by ELISA on Nunc polysorb plates coated with NP(8)-BSA or NP(36)-BSA (LGC, Biosearch Technologies) for capture. Immunoglobulin levels were detected by ELISA using SBA Clonotyping System HRP kit (Southern Biotech) according to the manufacturer’s instructions.

### SPR analysis

SPR measurements were performed on a Biacore Q instrument (Biacore) using a Sensor Chip NTA (Cytiva). The chip was charged with Ni^2+^ according to the manufacturer’s instructions, and recombinant His-tagged human SorLA protein (R&D Systems) was captured at 10 µg/ml. Binding experiments were performed by injecting analyte (IgM/IgG; concentrations ranging from 0 to 100 µg/ml) in HBS-P buffer (Cytiva), pH 7.4, at a flow rate of 10 μl/min at 25°C. Association and dissociation phases were monitored, and binding responses were recorded in response units and analyzed using Biacore evaluation software. A reference flow cell and buffer-only injections were used for background subtraction.

### Metabolic analysis using Agilent XF seahorse assay: Mito stress test

3 × 10^5^ cells were seeded/well in a poly-L-lysine–coated Agilent Seahorse XF Cell Culture Microplate. Plates were centrifuged at 200 g for 1 min with no brake, and cells were cultured in Seahorse XF RPMI medium (Agilent) supplemented with 11 mM glutamine, 2 mM glucose, and 1 mM sodium pyruvate for 30 min at 37°C in a non-CO_2_ incubator.

A XF sensor cartridge was hydrated with XF calibrant (Agilent) overnight at 37°C in a non-CO2 incubator. Metabolic stress compounds were diluted in Seahorse XF RPMI medium and added to injection ports for the following final concentrations: A—Seahorse XF RPMI medium with or without anti-IgM F(ab′)_2_ (5 μg/ml), B—Oligomycin (AOBIOUS INC) (5 μM), C—FCCP (APExBIO) (3 μM), and D—Rotenone (APExBIO) (2.5 μM) + Antimycin A (Sigma-Aldrich) (2.5 μM). Individual well (OCR and ECAR were measured on a Seahorse XFe96 Analyzer with Wave software.

### Statistics

For *in vivo* experiments, ELISA, soluble anti-IgM F(ab′)_2_ internalization, and Eα presentation, mice were age and sex matched, and investigator blinding through use of anonymous IDs was adopted throughout to avoid bias. Unless otherwise stated in figure legends, experimental units are individual mice. Sample sizes for individual experiments are detailed in figure legends. Appropriate statistical analysis was performed in GraphPad Prism version 11.0.0 for Windows (https://www.graphpad.com). Shapiro–Wilk normality test was used to determine if data were normally distributed for each dataset independently. Where normality could not be confidently assumed, nonparametric statistical tests were applied. P ≤ 0.05 indicates statistical significance; different levels of significance are shown in figures using standard notation.

### Online supplemental material

Fig. S1 shows resting and antigen-stimulated signalling downstream of the BCR (pSyk, pErk, and pAkt), showing no significant change in the phosphorylation cascade in the absence of SorLA. It also shows no difference in total RAB7 and RAB11 levels and no significant changes in IgM BCR recycling. Fig. S2 investigates lysosomal number and size, showing SorLA deletion does not alter global lysosomal organization in Ramos B cells. Fig. S3 shows gating strategies for bone marrow, lymph node, and spleen immunophenotyping. Fig. S4 describes the generation and validation of *SORL1*^KO^ versions of Ramos B cell lines expressing IgM BCRs with specificities of HIV-1 bnAbs CH31 and DH270.1. *SORL1* deletion in these cells results in reduced antigen uptake, similar to findings using surrogate antigen. Fig. S5 shows analysis of metabolic flux and cell viability, with no significant changes following *SORL1* deletion. It also illustrates the image analysis pipeline using the Labkit machine learning tool in IMARIS software.

## Supplementary Material

SourceData F2 is the source file for Fig. 2.

SourceData F4 is the source file for Fig. 4.

SourceData FS1 is the source file for Fig. S1.

SourceData FS4 is the source file for Fig. S4.

## Data Availability

The mass spectrometry proteomics data have been deposited to the ProteomeXchange Consortium via the PRIDE partner repository. All other data are available in the main text or the supplementary materials.
