# Supplementary material for: SorLA regulates B cell receptor uptake, trafficking, and B cell–mediated immune responses in vivo
Source: J Exp Med. 2026 Aug 4;223(9):e20252357. doi: 10.1084/jem.20252357 (PMC13435973; doi:10.1084/jem.20252357)
Supplement: Supplementary file 1 [file jem_20252357_sourcedataf2.pdf]

2C

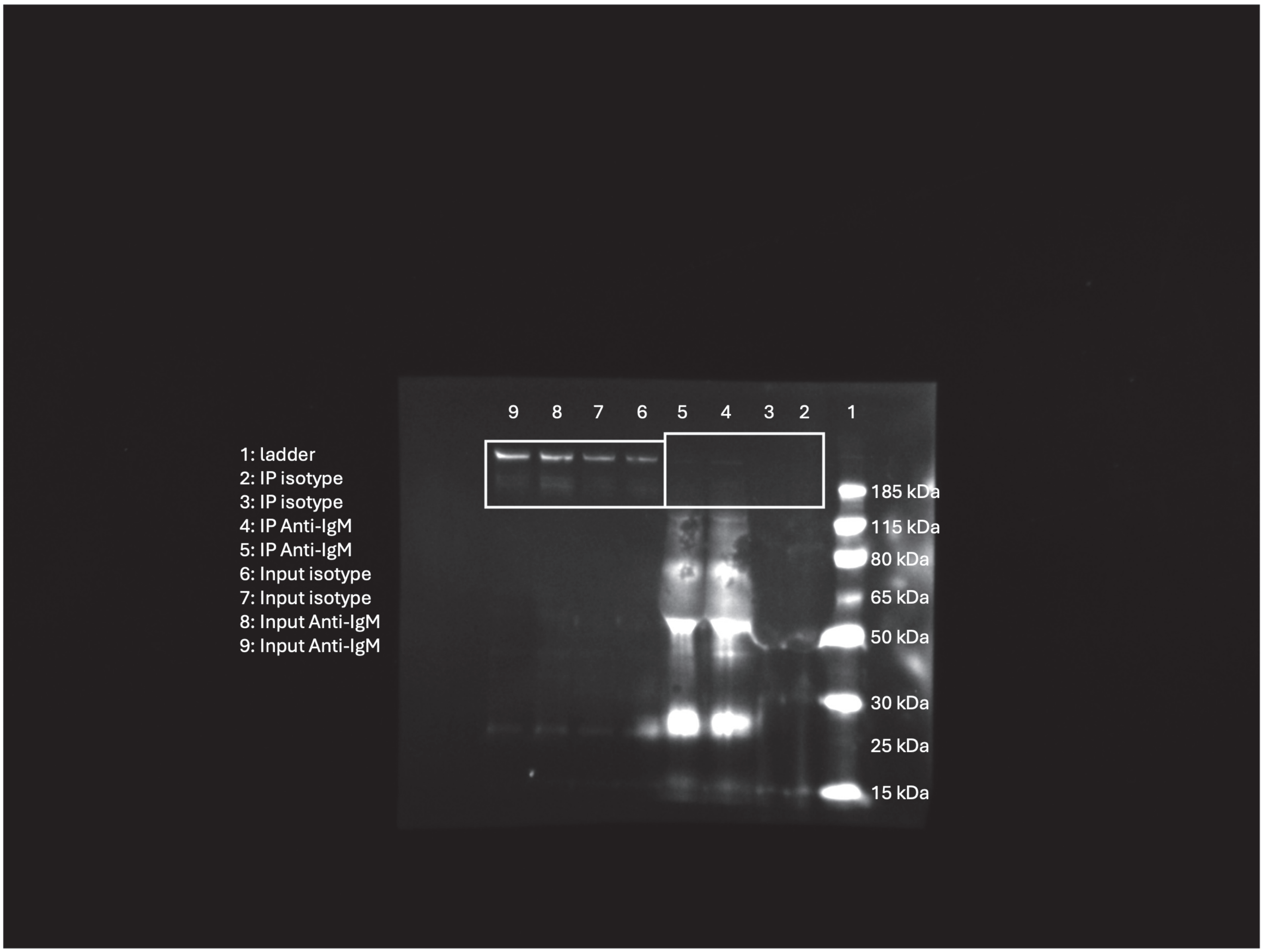

9 8 7 6 5 4 3 2 1
1: ladder
2: IP isotype
3: IP isotype
4: IP Anti-IgM
5: IP Anti-IgM
6: Input isotype
7: Input isotype
8: Input Anti-IgM
9: Input Anti-IgM
185 kDa
115 kDa
80 kDa
65 kDa
50 kDa
30 kDa
25 kDa
15 kDa

2F

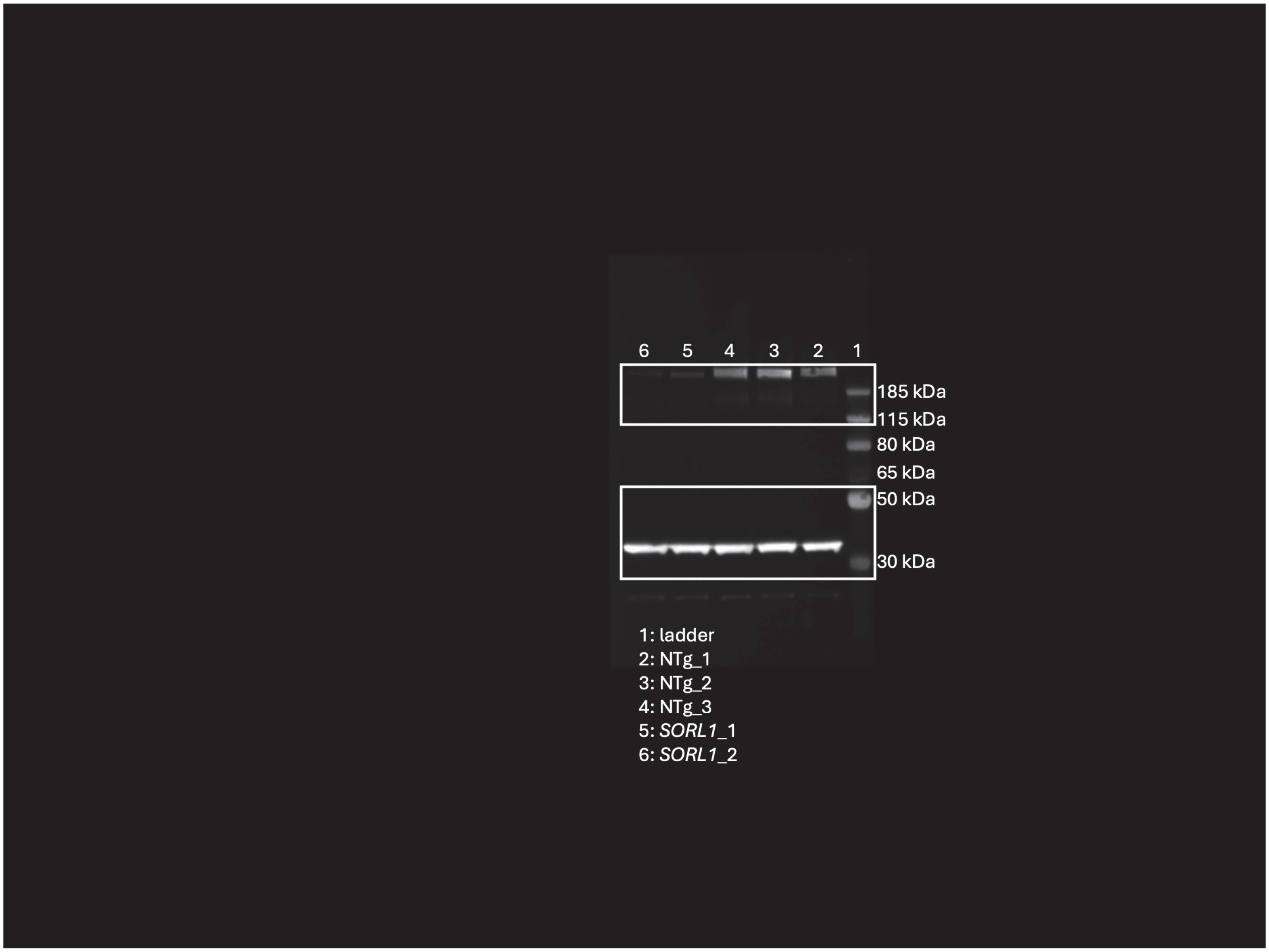
6
5
4
3
2
1
185 kDa
115 kDa
80 kDa
65 kDa
50 kDa
30 kDa
1: ladder
2: NTg_1
3: NTg_2
4: NTg_3
5: SORL1_1
6: SORL1_2

2H

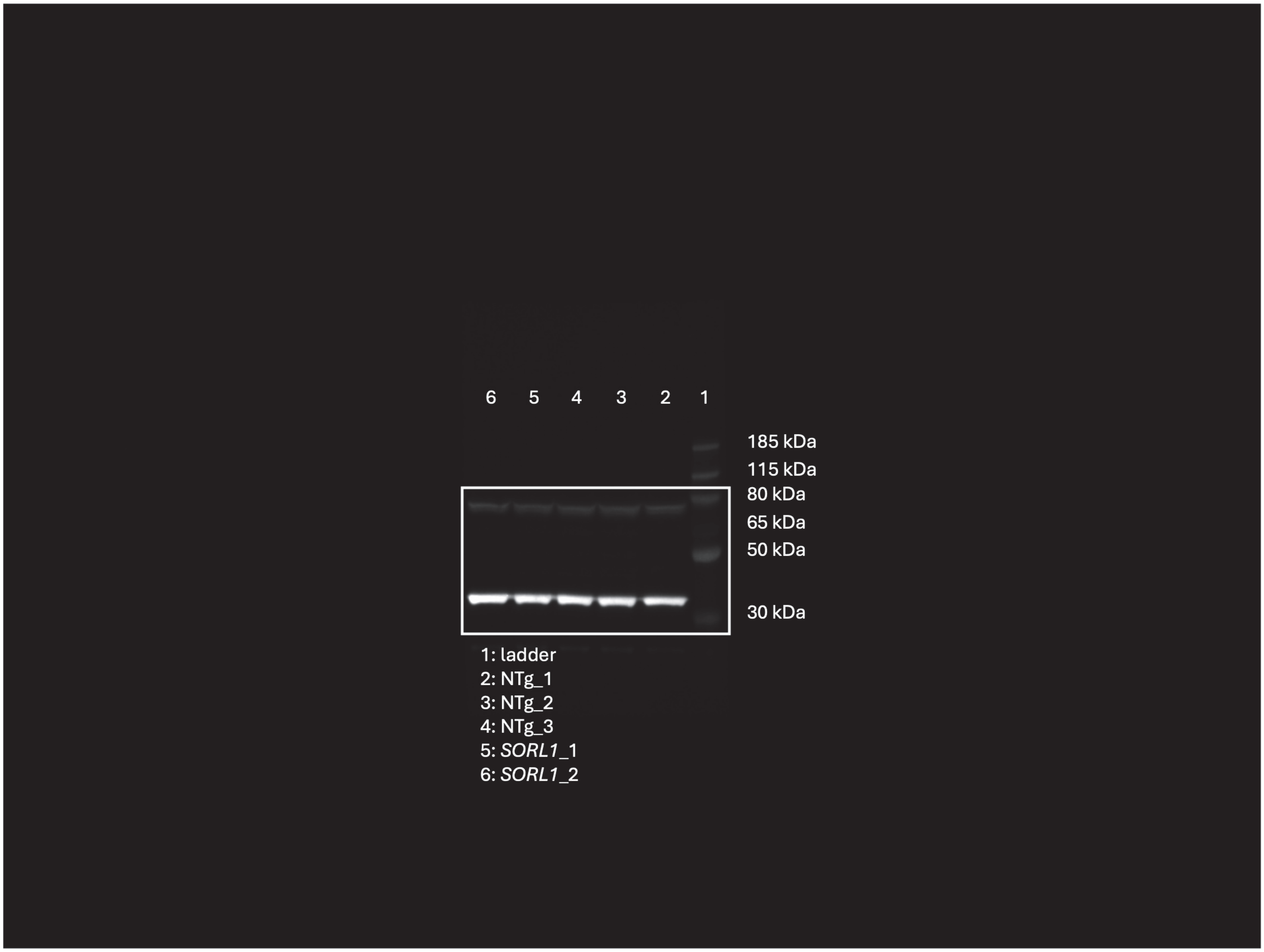
6 5 4 3 2 1
185 kDa
115 kDa
80 kDa
65 kDa
50 kDa
30 kDa
1: ladder
2: NTg_1
3: NTg_2
4: NTg_3
5: *SORL1*_1
6: *SORL1*_2
