# Supplementary material for: SorLA regulates B cell receptor uptake, trafficking, and B cell–mediated immune responses in vivo
Source: J Exp Med. 2026 Aug 4;223(9):e20252357. doi: 10.1084/jem.20252357 (PMC13435973; doi:10.1084/jem.20252357)
Supplement: Supplementary file 2 [file jem_20252357_sourcedataf4.pdf]

4E

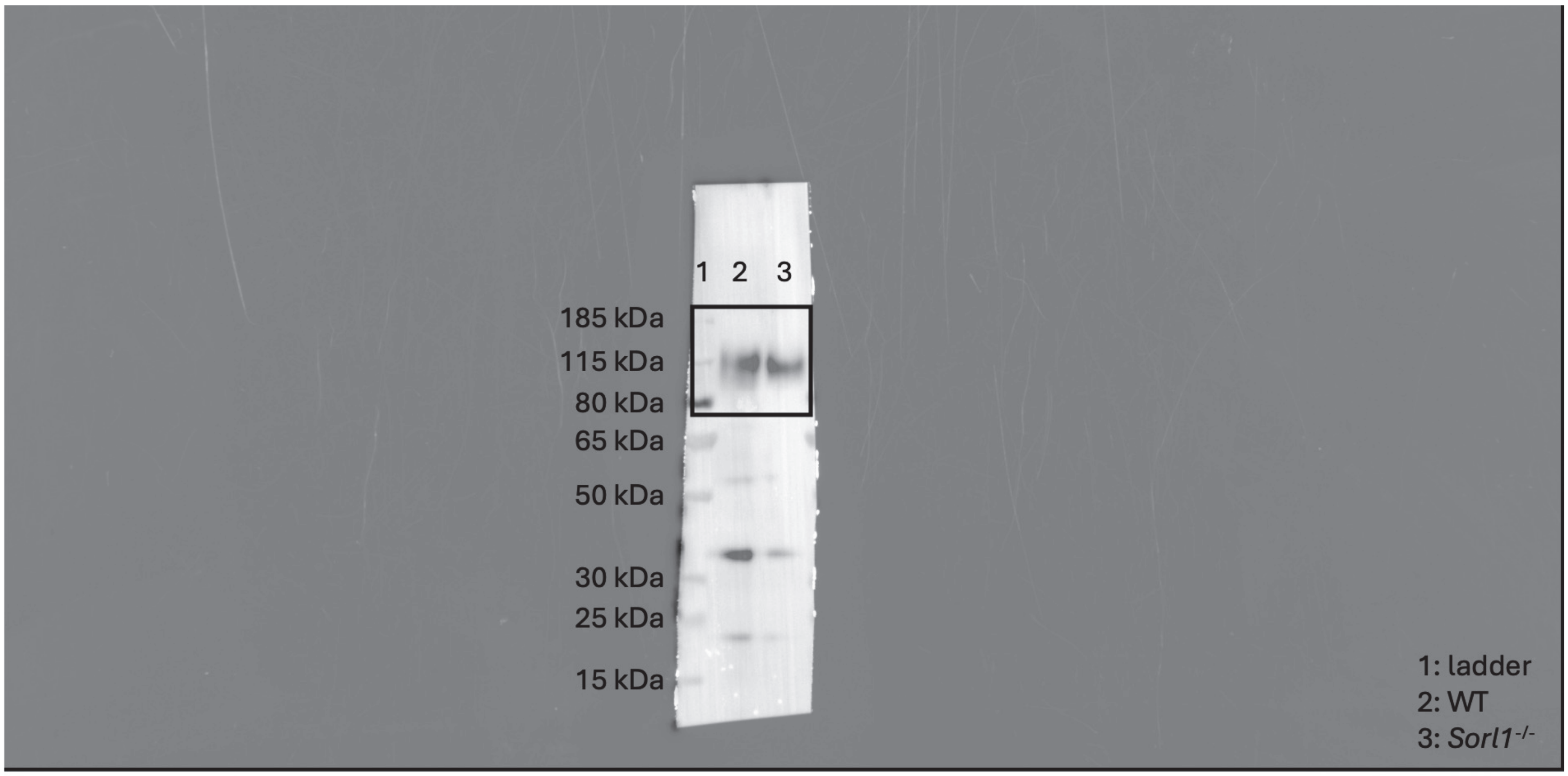
1 2 3
185 kDa
115 kDa
80 kDa
65 kDa
50 kDa
30 kDa
25 kDa
15 kDa
1: ladder
2: WT
3: *Sorl1*-/-

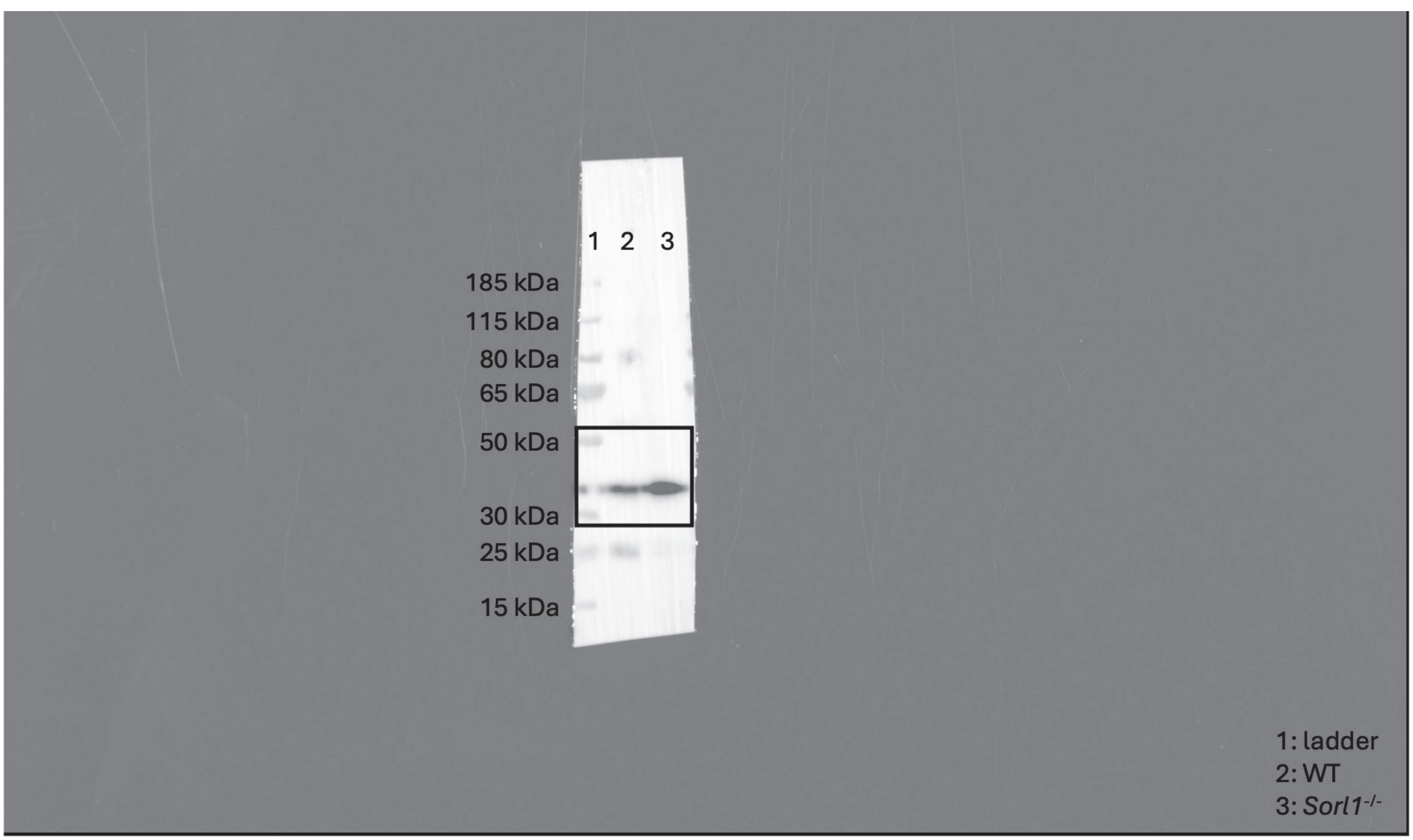
1 2 3
185 kDa
115 kDa
80 kDa
65 kDa
50 kDa
30 kDa
25 kDa
15 kDa
1: ladder
2: WT
3: *Sorl1*-/-

4F

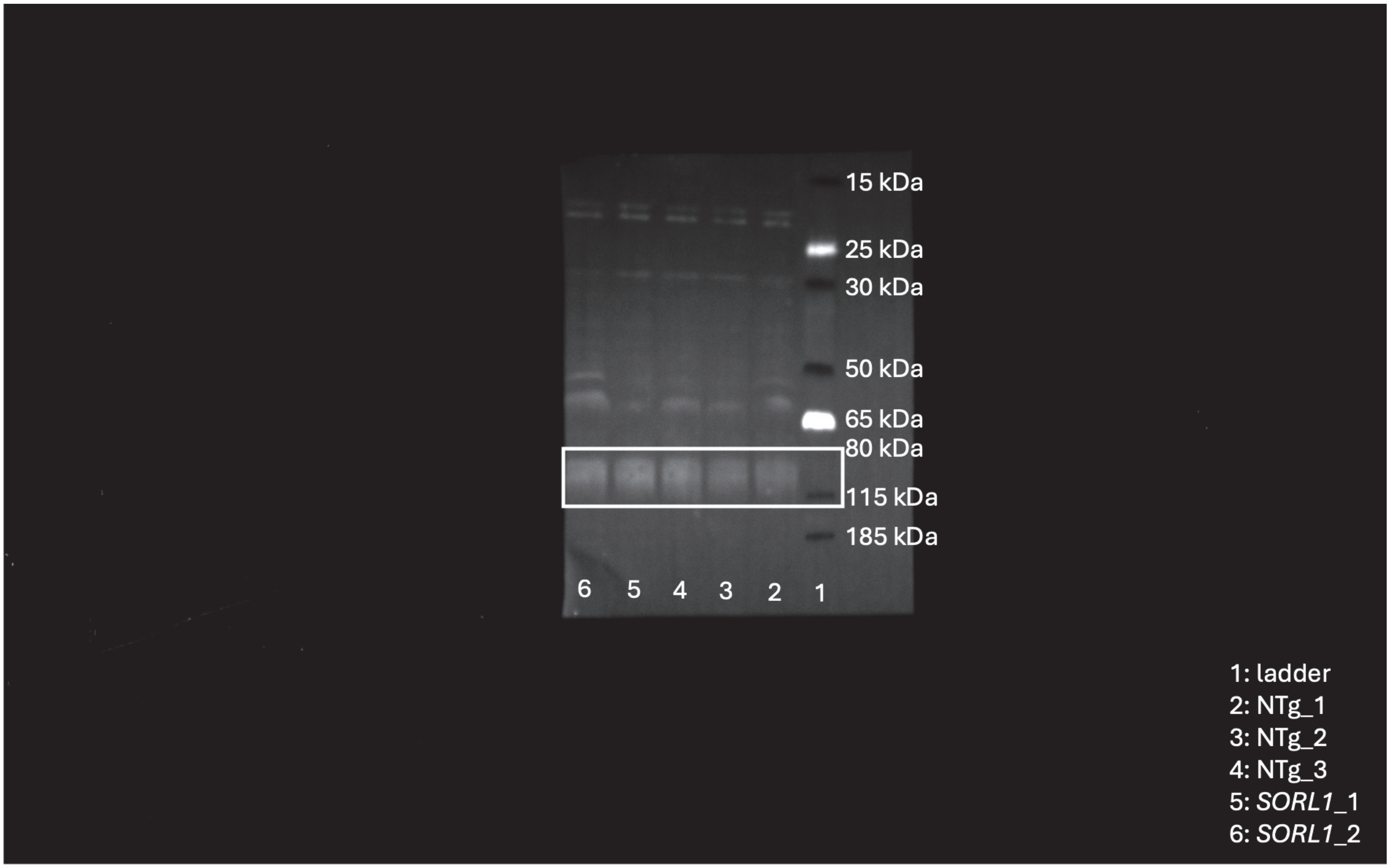
15 kDa
25 kDa
30 kDa
50 kDa
65 kDa
80 kDa
115 kDa
185 kDa
6 5 4 3 2 1
1: ladder
2: NTg_1
3: NTg_2
4: NTg_3
5: *SORL1*_1
6: *SORL1*_2

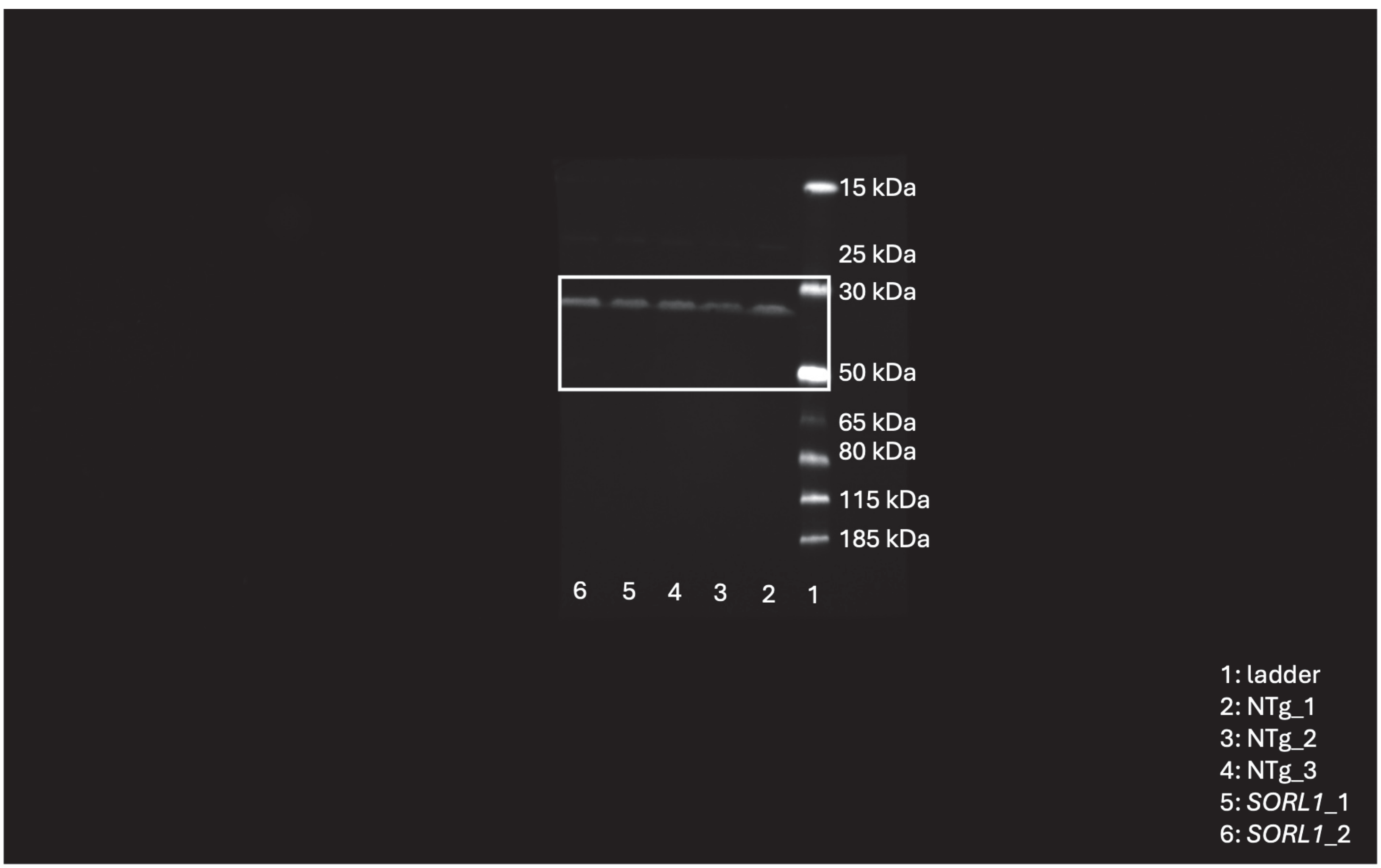
15 kDa
25 kDa
30 kDa
50 kDa
65 kDa
80 kDa
115 kDa
185 kDa
6 5 4 3 2 1
1: ladder
2: NTg_1
3: NTg_2
4: NTg_3
5: *SORL1*_1
6: *SORL1*_2

4H

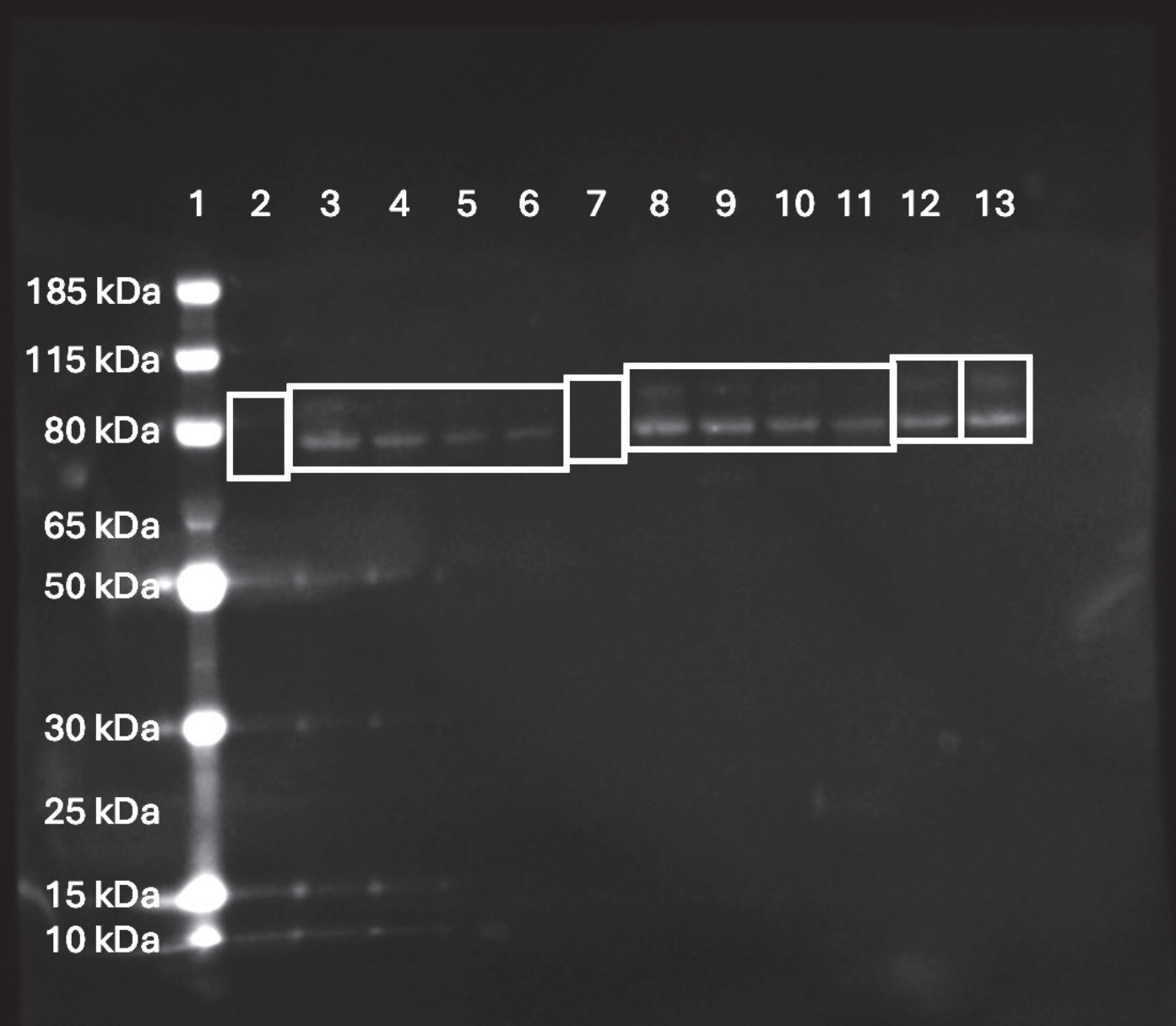

1: ladder
2: NTg_1 No biotin
3: NTg_1 0 hours
4: NTg_1 3 hours
5: NTg_1 6 hours
6: NTg_1 12 hours
7: *SORL1*_1 No biotin
8: *SORL1*_1 0 hours
9: *SORL1*_1 3 hours
10: *SORL1*_1 6 hours
11: *SORL1*_1 12 hours
12: NTg_1 No anti-IgM F(ab')$_2$
13: *SORL1*_1 No anti-IgM F(ab')$_2$

4K

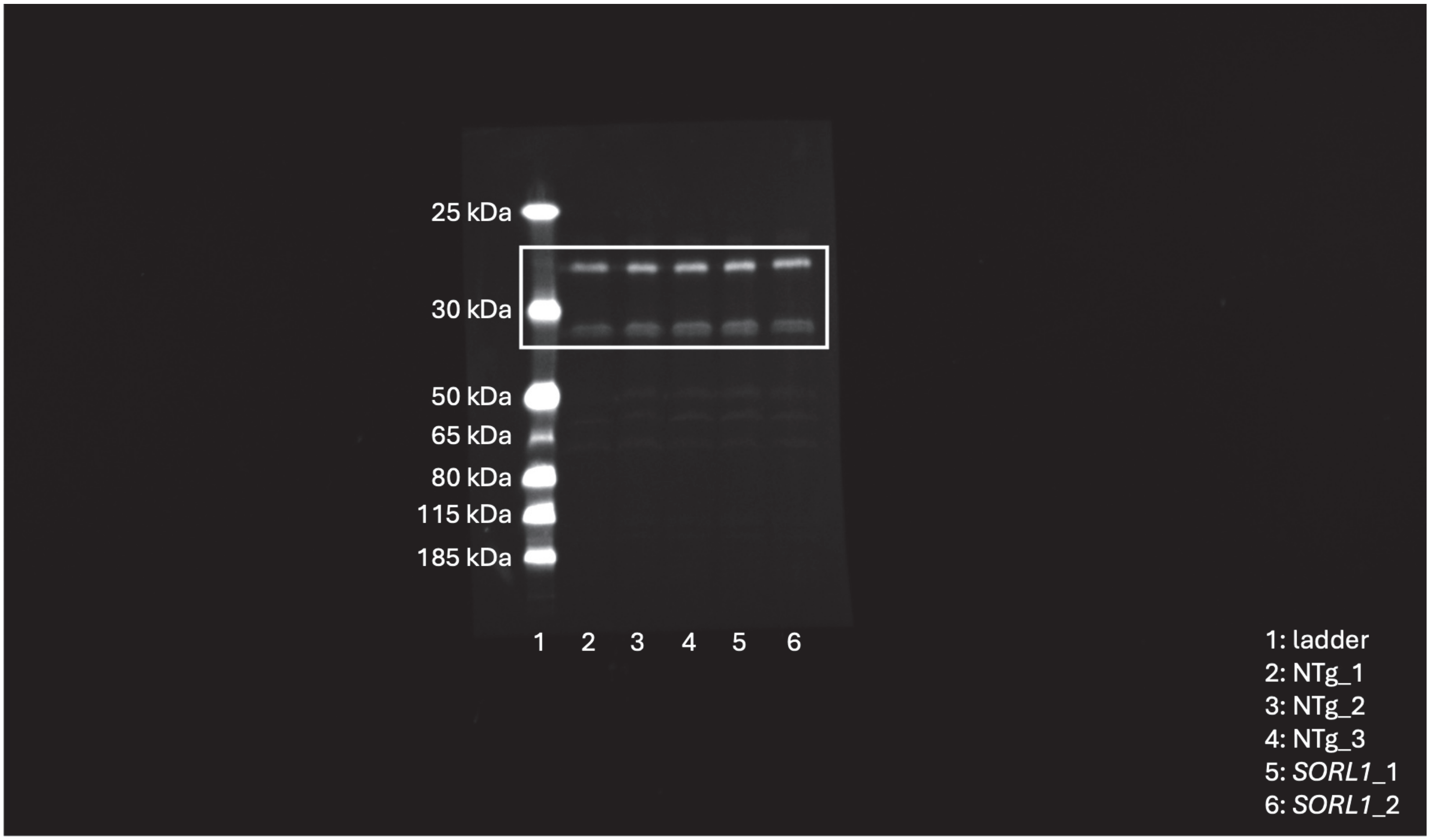

25 kDa
30 kDa
50 kDa
65 kDa
80 kDa
115 kDa
185 kDa
1 2 3 4 5 6
1: ladder
2: NTg_1
3: NTg_2
4: NTg_3
5: *SORL1*_1
6: *SORL1*_2

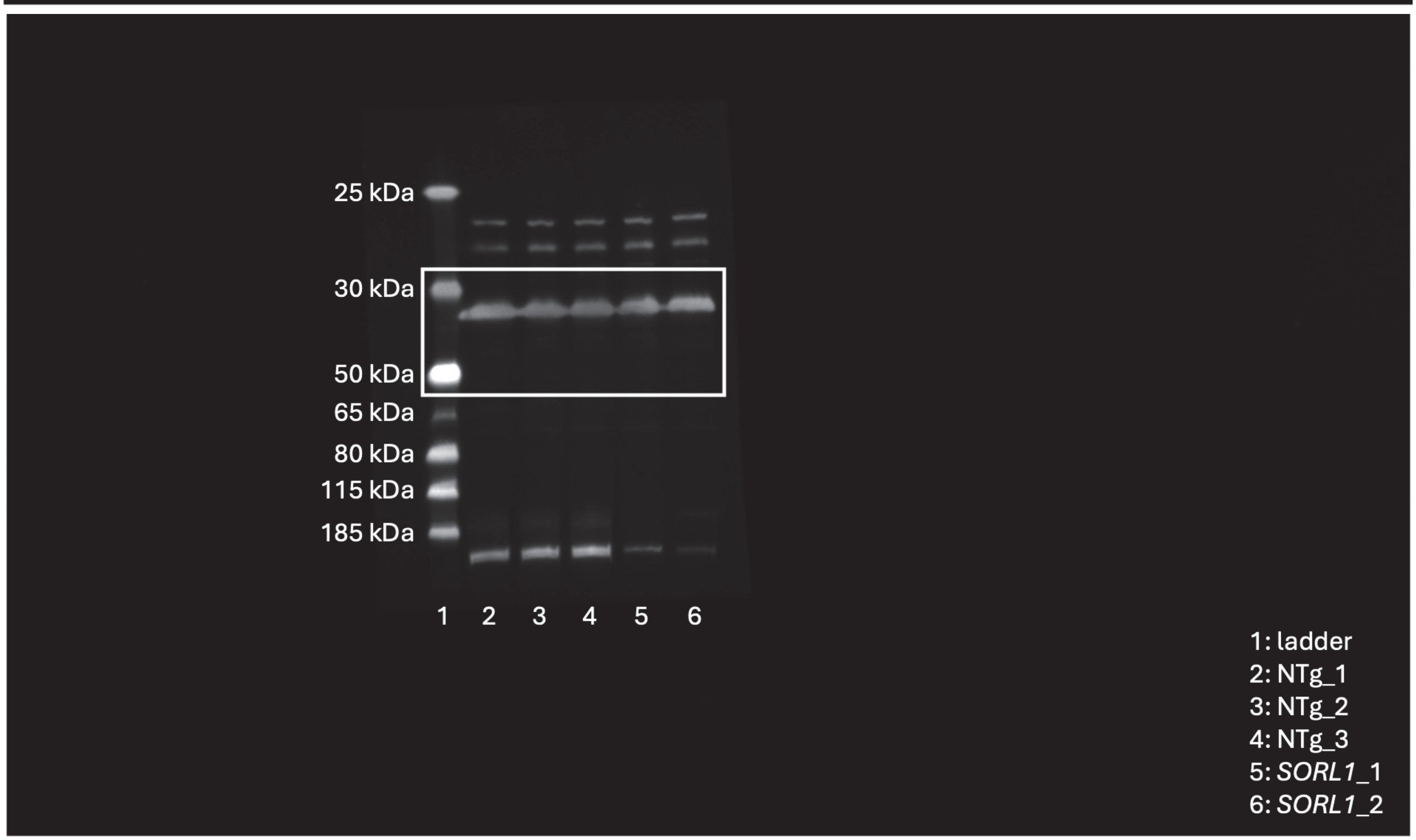

25 kDa
30 kDa
50 kDa
65 kDa
80 kDa
115 kDa
185 kDa
1 2 3 4 5 6
1: ladder
2: NTg_1
3: NTg_2
4: NTg_3
5: *SORL1*_1
6: *SORL1*_2
