# Supplementary material for: SorLA regulates B cell receptor uptake, trafficking, and B cell–mediated immune responses in vivo
Source: J Exp Med. 2026 Aug 4;223(9):e20252357. doi: 10.1084/jem.20252357 (PMC13435973; doi:10.1084/jem.20252357)
Supplement: Supplementary file 3 [file jem_20252357_sourcedatafs1.pdf]

SF 1A

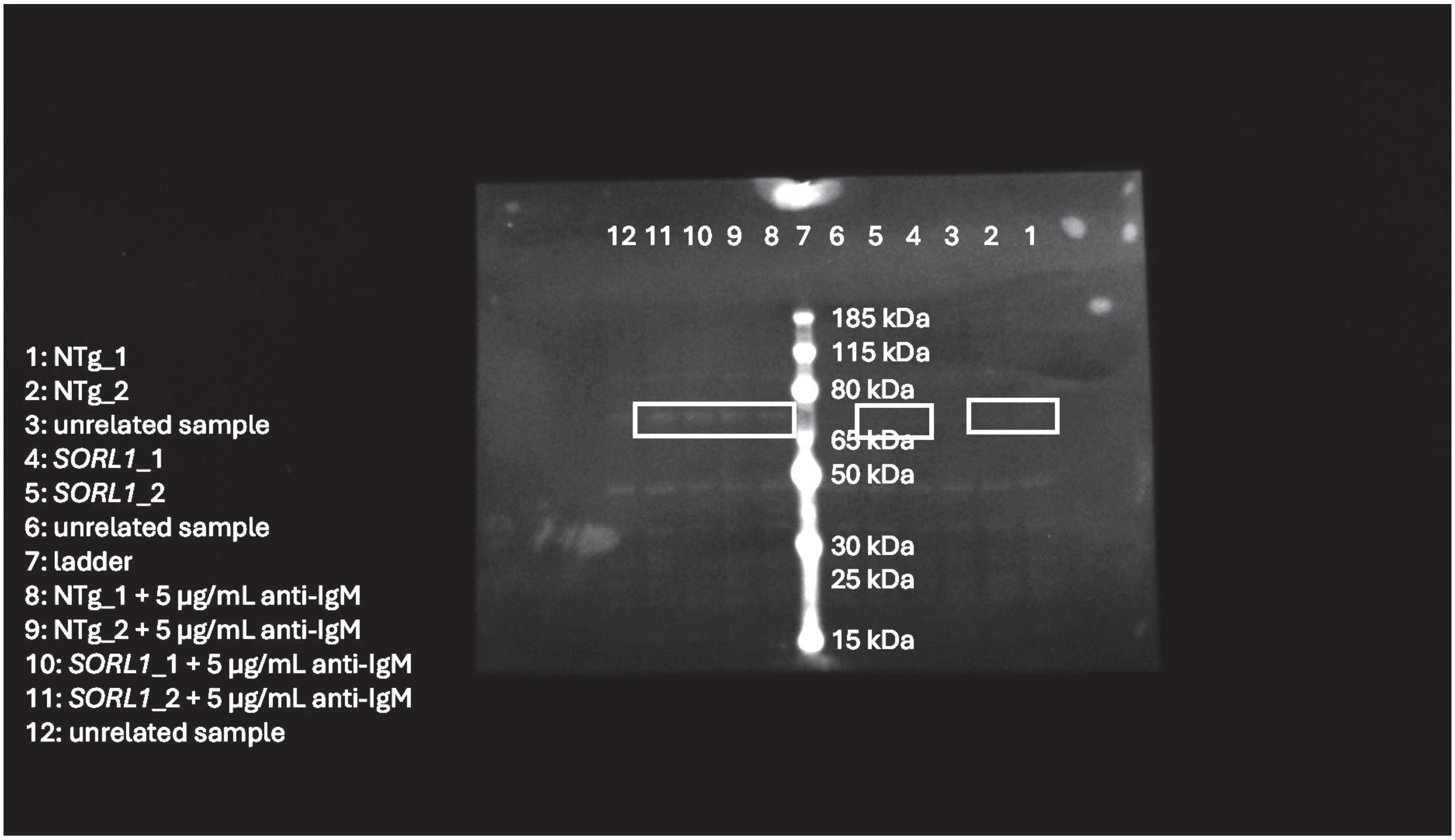
12 11 10 9 8 7 6 5 4 3 2 1
185 kDa
115 kDa
80 kDa
65 kDa
50 kDa
30 kDa
25 kDa
15 kDa
1: NTg_1
2: NTg_2
3: unrelated sample
4: SORL1_1
5: SORL1_2
6: unrelated sample
7: ladder
8: NTg_1 + 5 µg/mL anti-IgM
9: NTg_2 + 5 µg/mL anti-IgM
10: SORL1_1 + 5 µg/mL anti-IgM
11: SORL1_2 + 5 µg/mL anti-IgM
12: unrelated sample

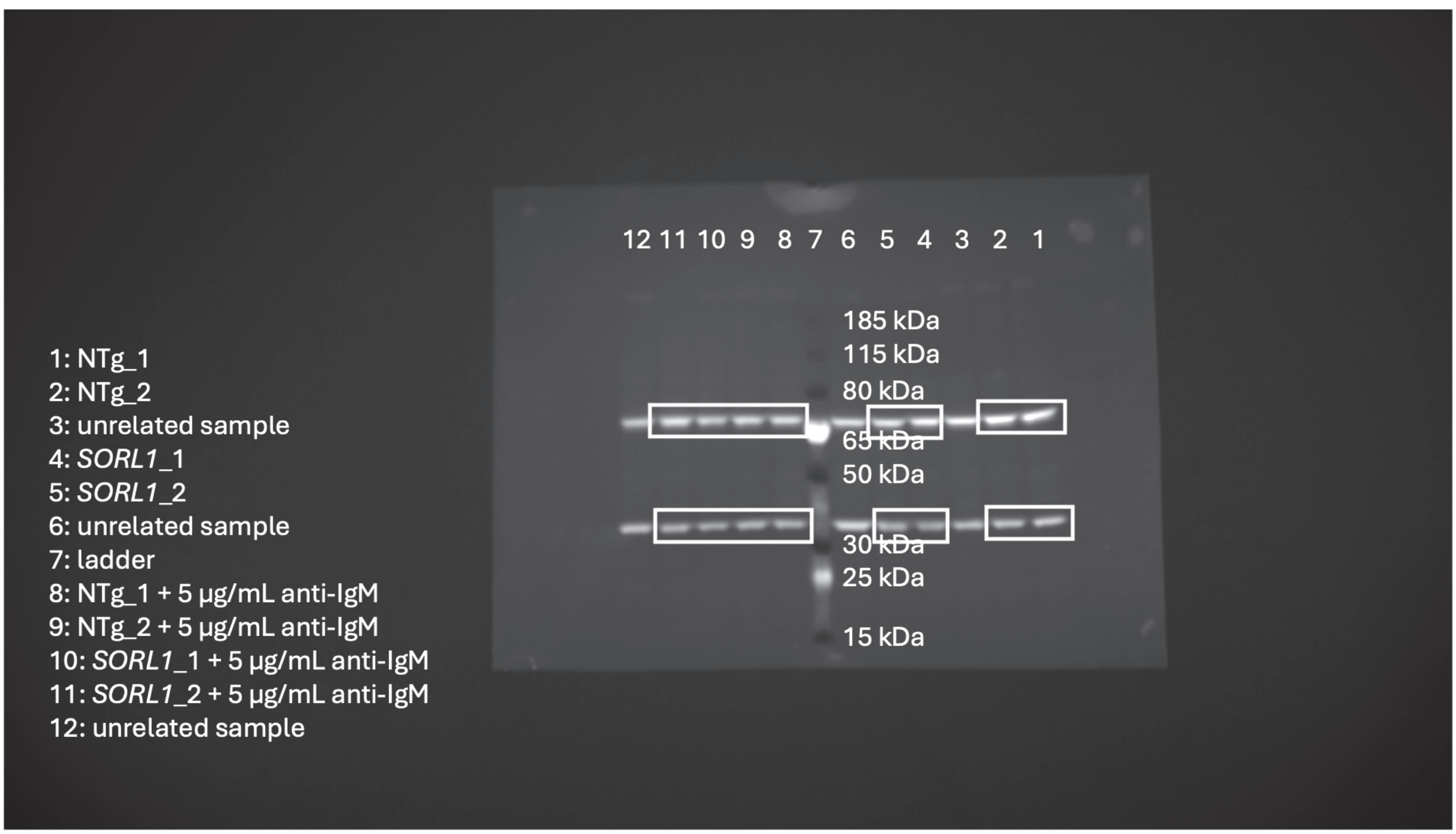
12 11 10 9 8 7 6 5 4 3 2 1
185 kDa
115 kDa
80 kDa
65 kDa
50 kDa
30 kDa
25 kDa
15 kDa
1: NTg_1
2: NTg_2
3: unrelated sample
4: SORL1_1
5: SORL1_2
6: unrelated sample
7: ladder
8: NTg_1 + 5 µg/mL anti-IgM
9: NTg_2 + 5 µg/mL anti-IgM
10: SORL1_1 + 5 µg/mL anti-IgM
11: SORL1_2 + 5 µg/mL anti-IgM
12: unrelated sample

# SF 1D

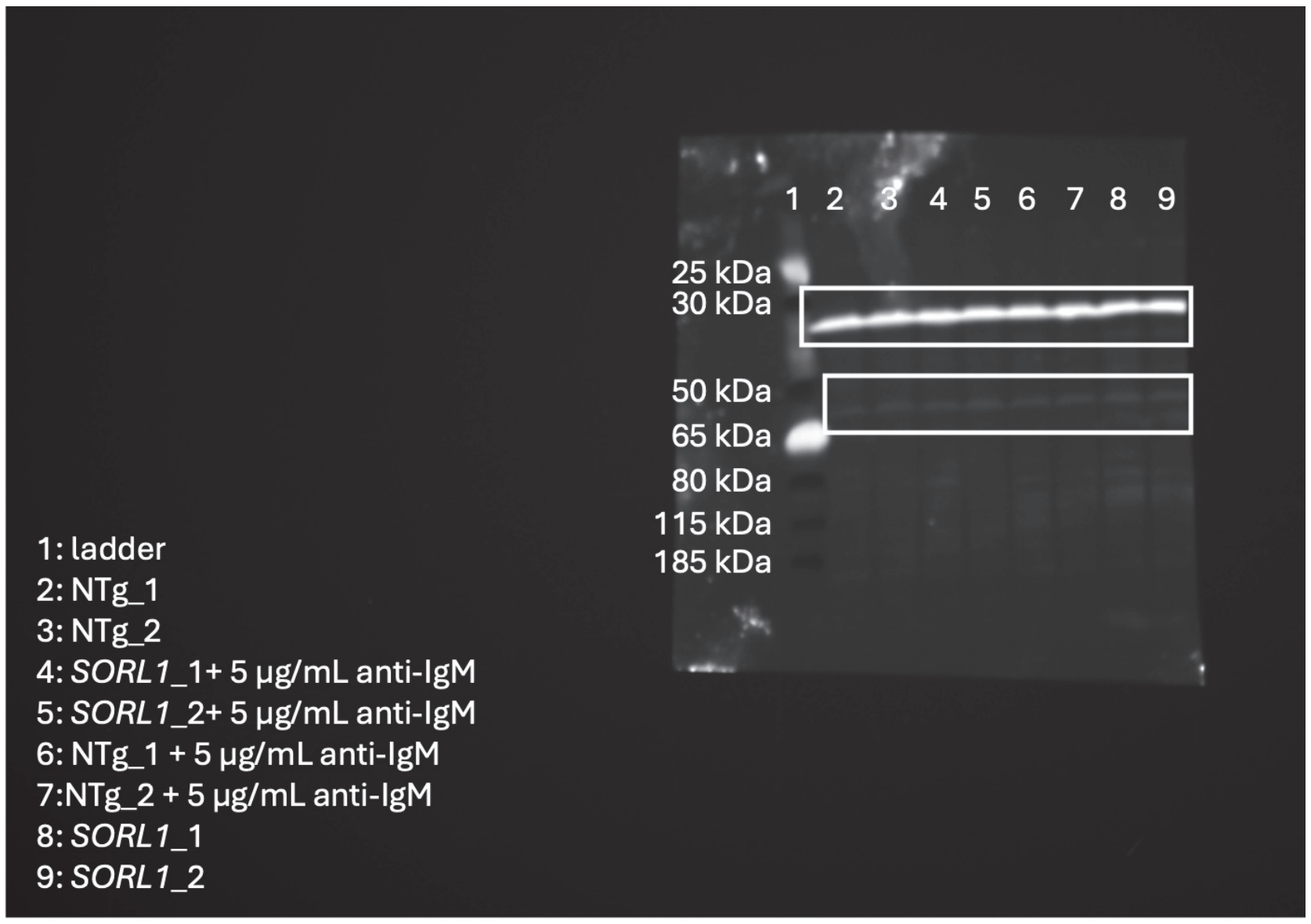
1 2 3 4 5 6 7 8 9
25 kDa
30 kDa
50 kDa
65 kDa
80 kDa
115 kDa
185 kDa
1: ladder
2: NTg_1
3: NTg_2
4: *SORL1*_1+ 5 µg/mL anti-IgM
5: *SORL1*_2+ 5 µg/mL anti-IgM
6: NTg_1 + 5 µg/mL anti-IgM
7:NTg_2 + 5 µg/mL anti-IgM
8: *SORL1*_1
9: *SORL1*_2

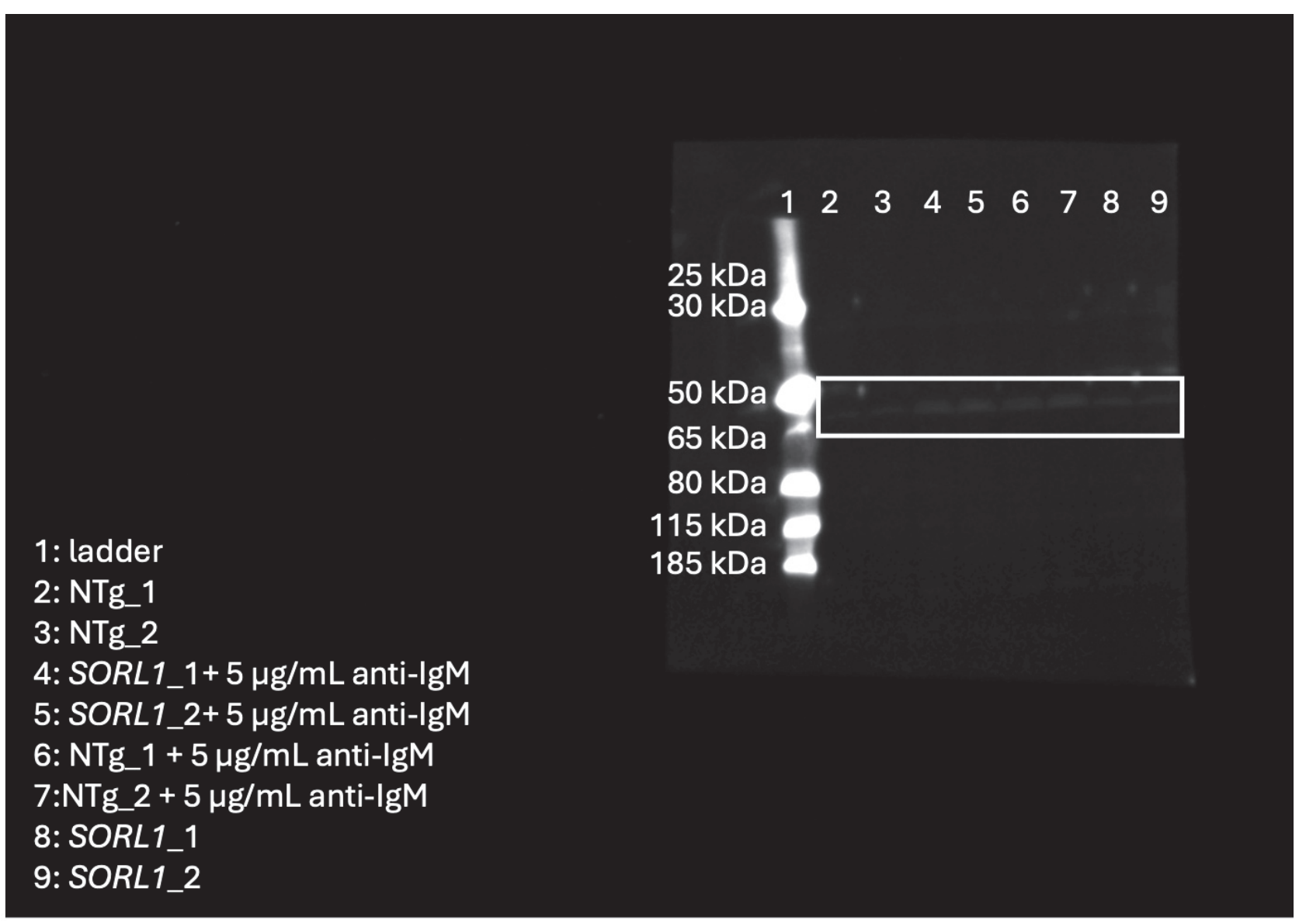
1 2 3 4 5 6 7 8 9
25 kDa
30 kDa
50 kDa
65 kDa
80 kDa
115 kDa
185 kDa
1: ladder
2: NTg_1
3: NTg_2
4: *SORL1*_1+ 5 µg/mL anti-IgM
5: *SORL1*_2+ 5 µg/mL anti-IgM
6: NTg_1 + 5 µg/mL anti-IgM
7:NTg_2 + 5 µg/mL anti-IgM
8: *SORL1*_1
9: *SORL1*_2

# SF 1D

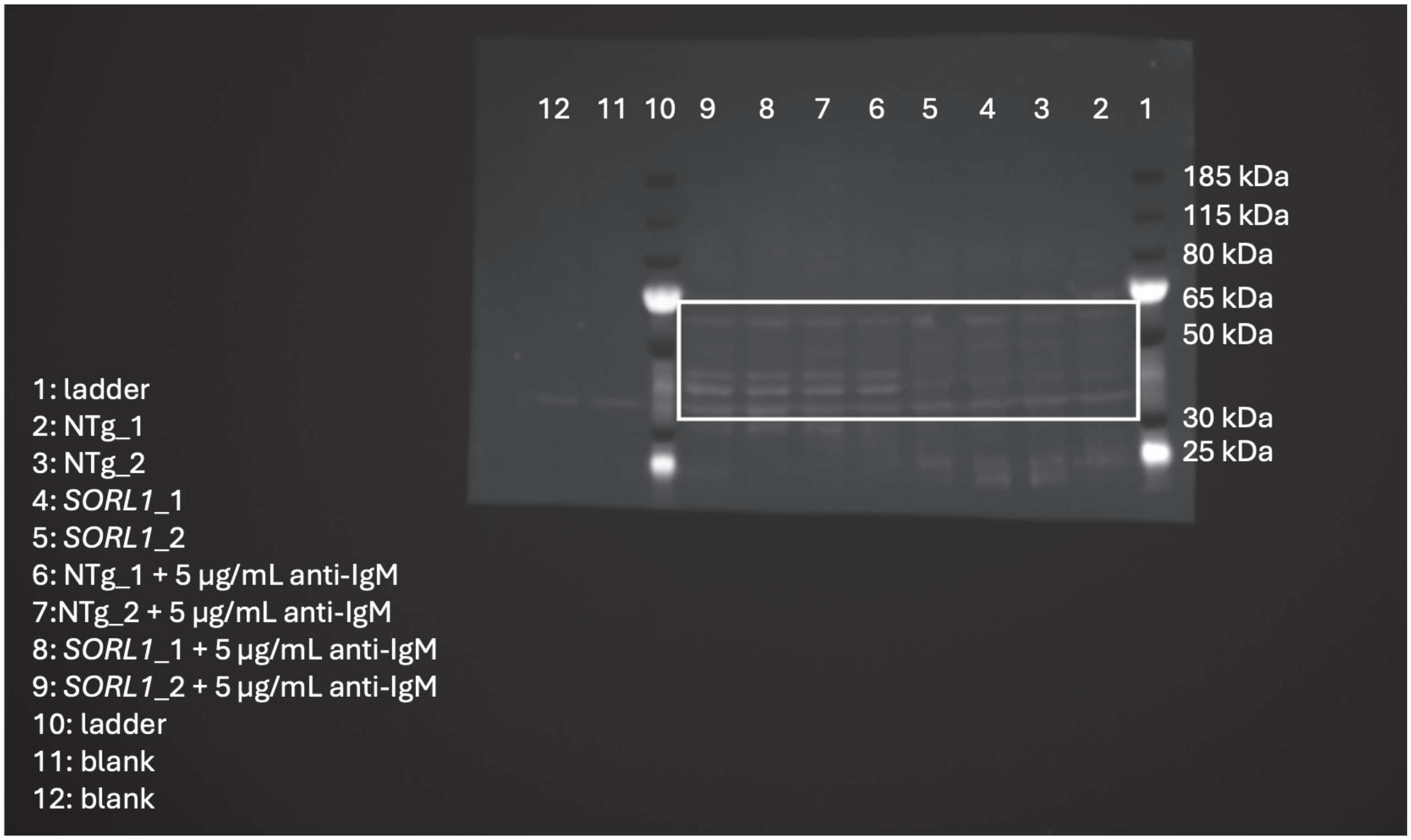
12 11 10 9 8 7 6 5 4 3 2 1
185 kDa
115 kDa
80 kDa
65 kDa
50 kDa
30 kDa
25 kDa
1: ladder
2: NTg_1
3: NTg_2
4: *SORL1*_1
5: *SORL1*_2
6: NTg_1 + 5 μg/mL anti-IgM
7:NTg_2 + 5 μg/mL anti-IgM
8: *SORL1*_1 + 5 μg/mL anti-IgM
9: *SORL1*_2 + 5 μg/mL anti-IgM
10: ladder
11: blank
12: blank

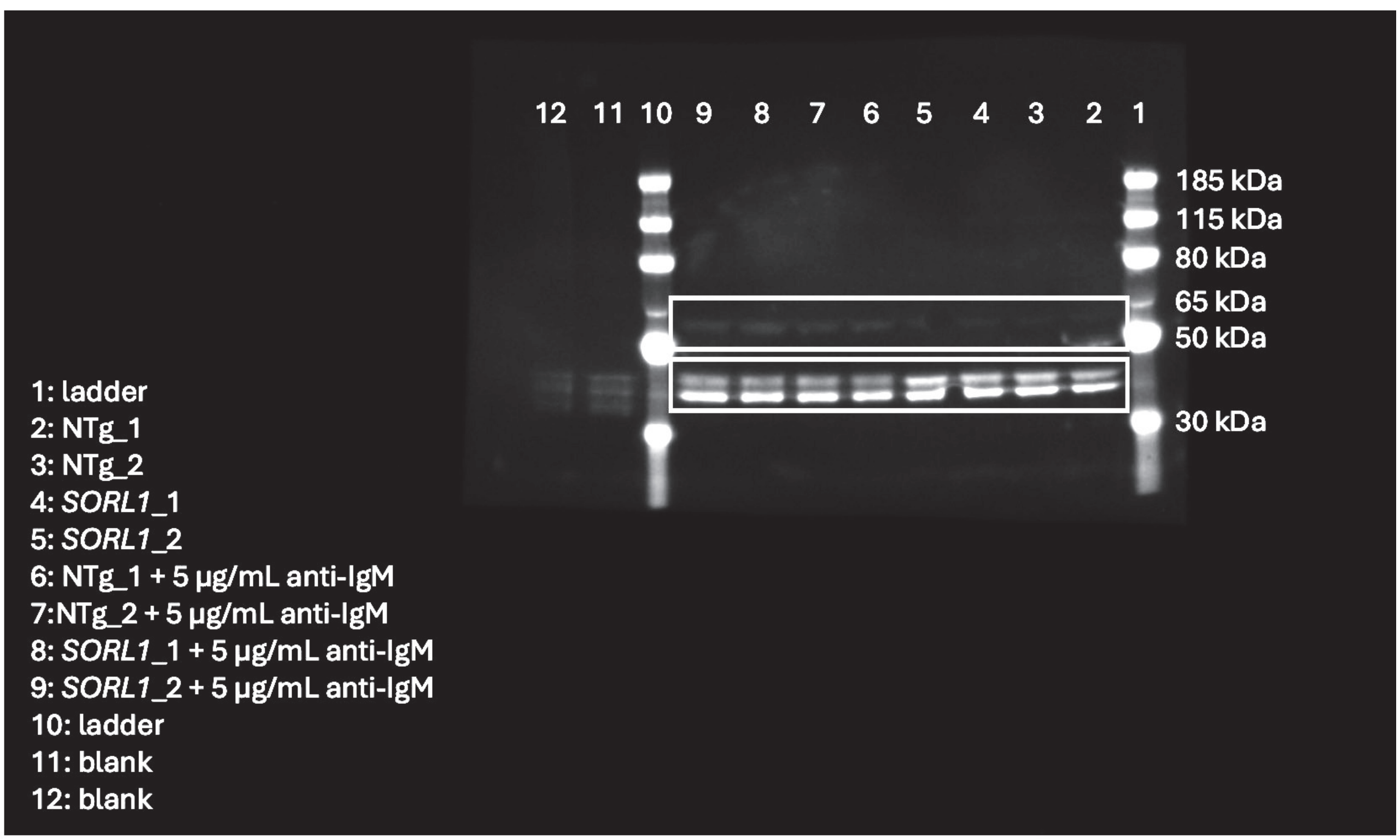
12 11 10 9 8 7 6 5 4 3 2 1
185 kDa
115 kDa
80 kDa
65 kDa
50 kDa
30 kDa
1: ladder
2: NTg_1
3: NTg_2
4: *SORL1*_1
5: *SORL1*_2
6: NTg_1 + 5 μg/mL anti-IgM
7:NTg_2 + 5 μg/mL anti-IgM
8: *SORL1*_1 + 5 μg/mL anti-IgM
9: *SORL1*_2 + 5 μg/mL anti-IgM
10: ladder
11: blank
12: blank

# SF 1J

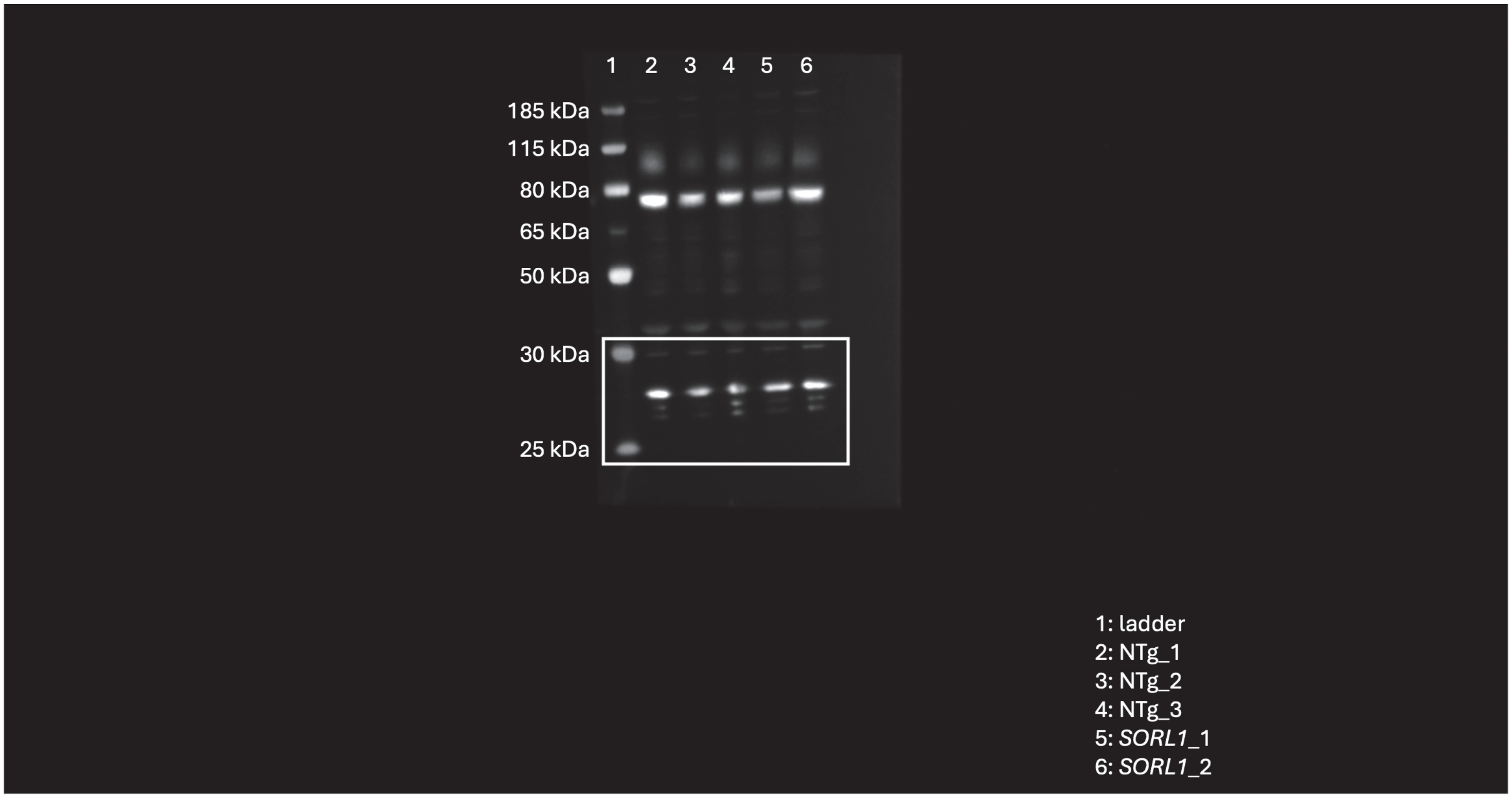
1 2 3 4 5 6
185 kDa
115 kDa
80 kDa
65 kDa
50 kDa
30 kDa
25 kDa
1: ladder
2: NTg_1
3: NTg_2
4: NTg_3
5: *SORL1*_1
6: *SORL1*_2

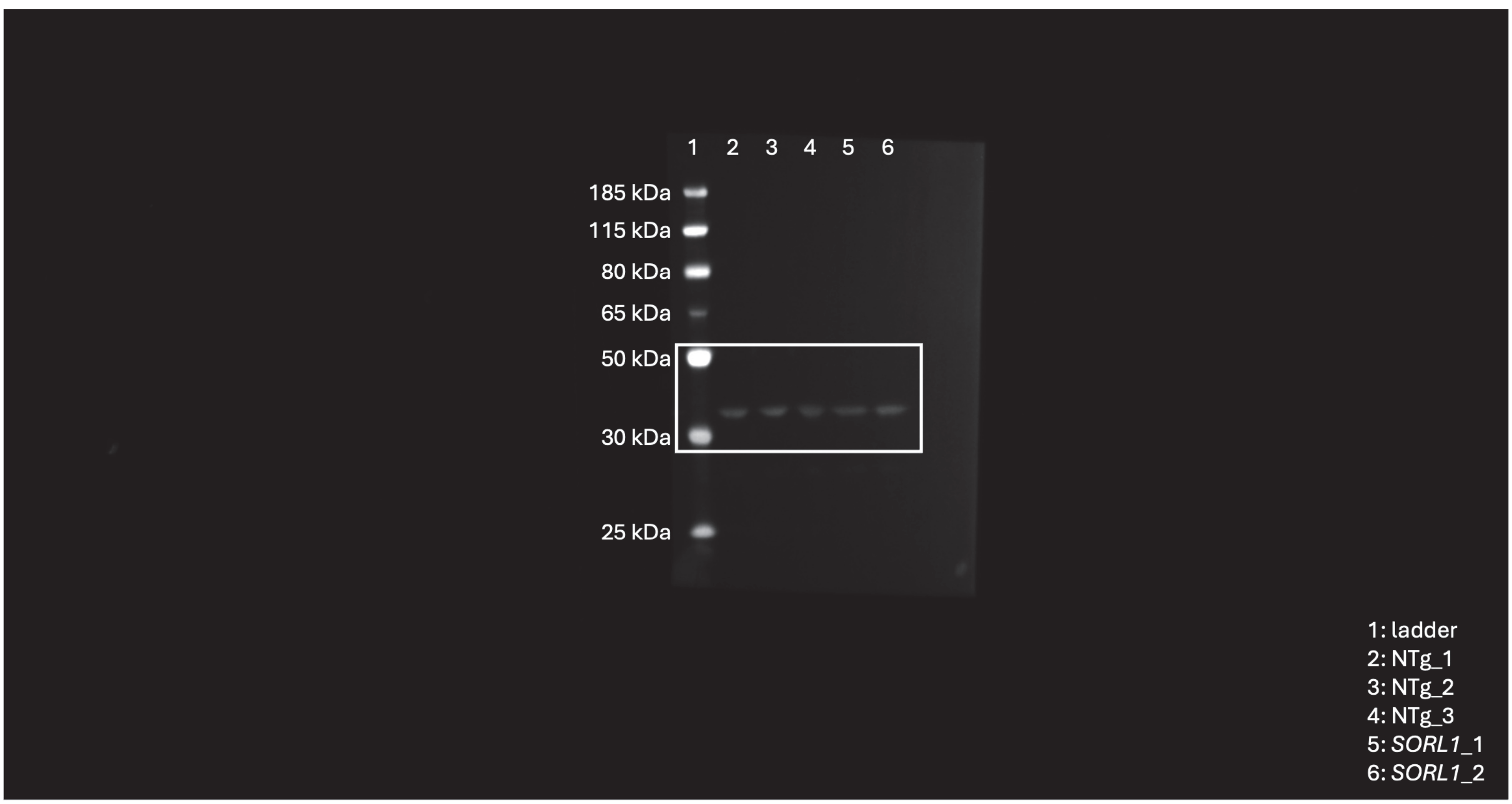
1 2 3 4 5 6
185 kDa
115 kDa
80 kDa
65 kDa
50 kDa
30 kDa
25 kDa
1: ladder
2: NTg_1
3: NTg_2
4: NTg_3
5: *SORL1*_1
6: *SORL1*_2

# SF 1K

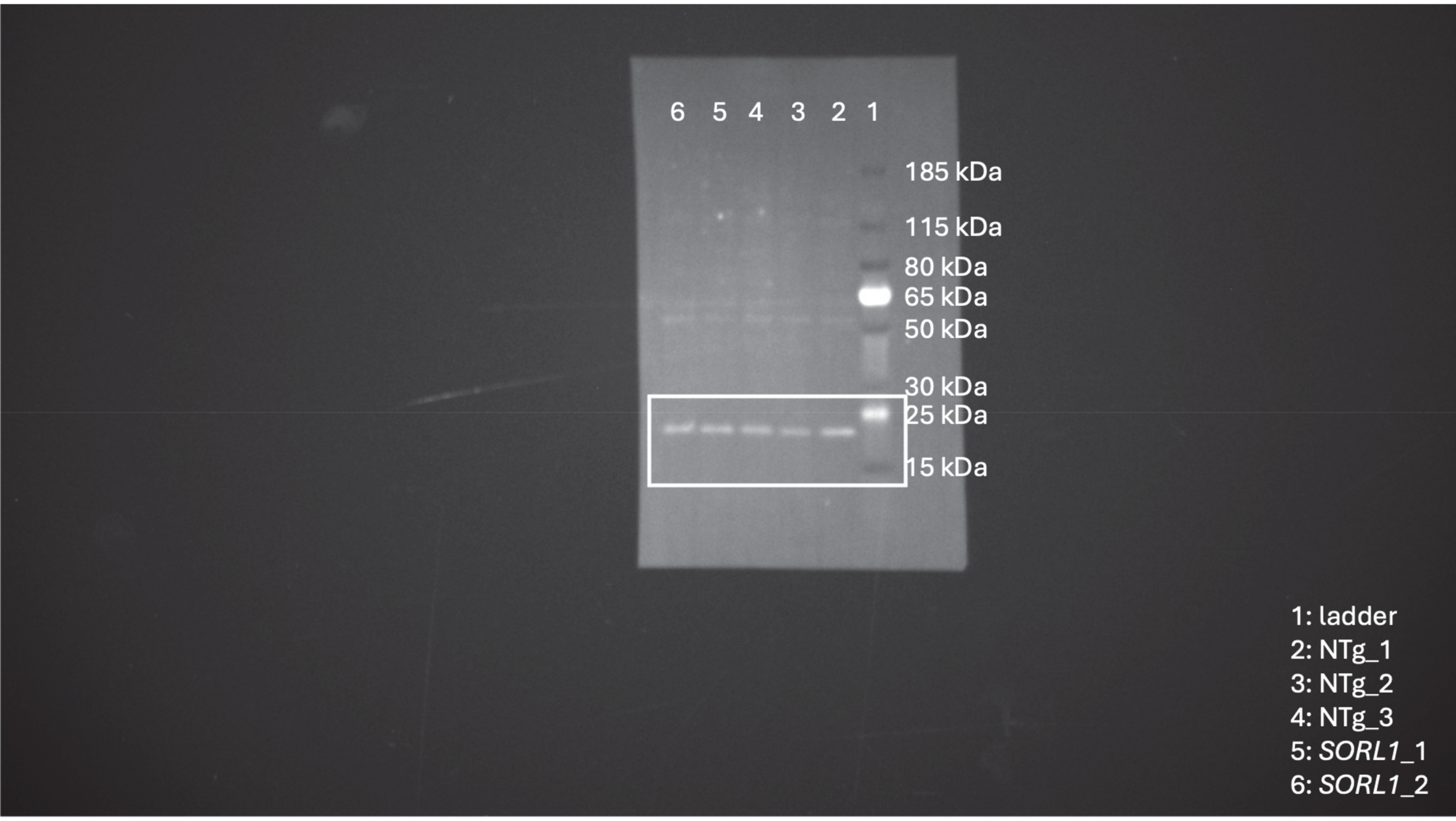
6 5 4 3 2 1
185 kDa
115 kDa
80 kDa
65 kDa
50 kDa
30 kDa
25 kDa
15 kDa
1: ladder
2: NTg_1
3: NTg_2
4: NTg_3
5: *SORL1*_1
6: *SORL1*_2

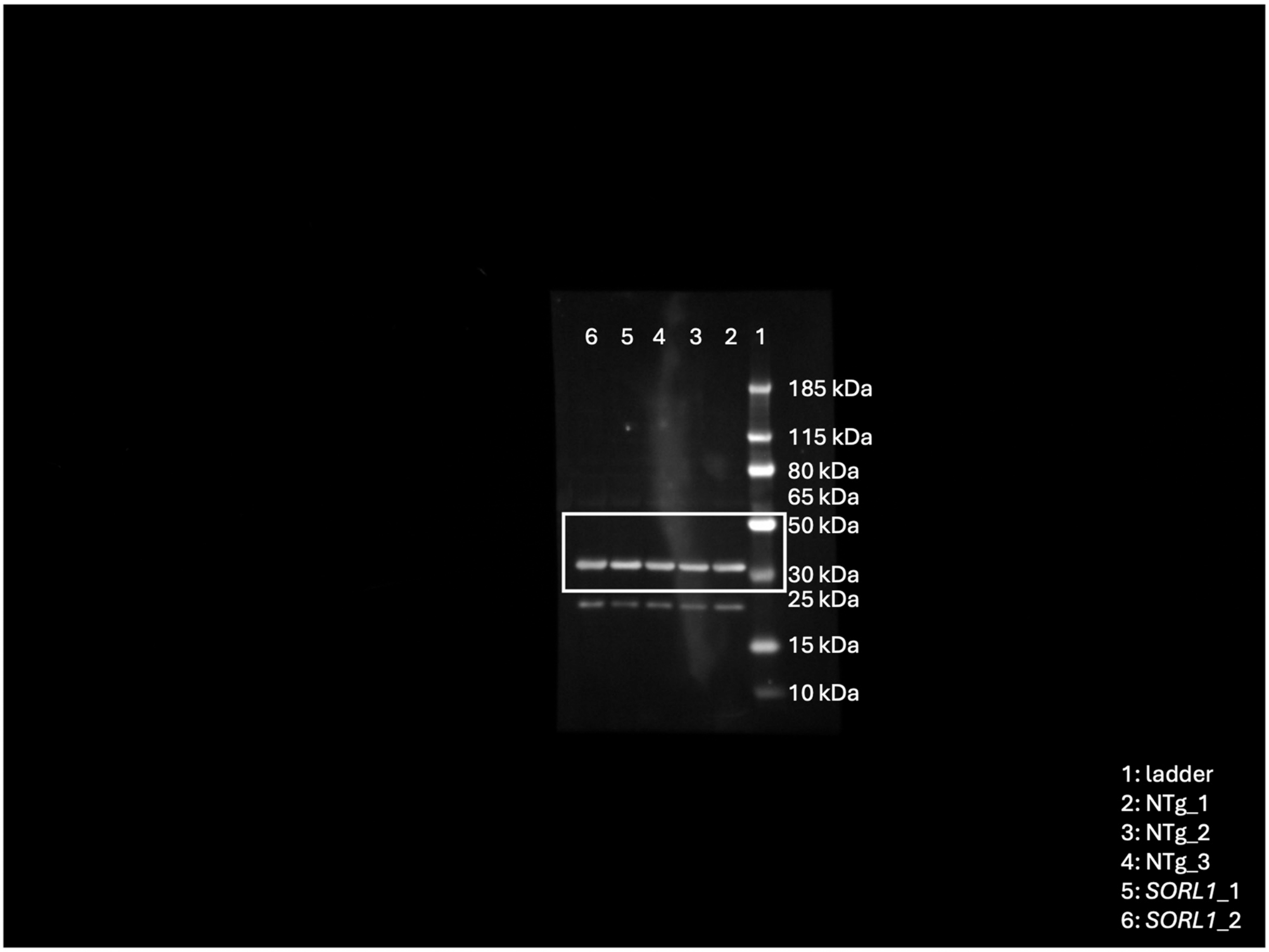
6 5 4 3 2 1
185 kDa
115 kDa
80 kDa
65 kDa
50 kDa
30 kDa
25 kDa
15 kDa
10 kDa
1: ladder
2: NTg_1
3: NTg_2
4: NTg_3
5: *SORL1*_1
6: *SORL1*_2
