# Supplementary material for: SorLA regulates B cell receptor uptake, trafficking, and B cell–mediated immune responses in vivo
Source: J Exp Med. 2026 Aug 4;223(9):e20252357. doi: 10.1084/jem.20252357 (PMC13435973; doi:10.1084/jem.20252357)
Supplement: Supplementary file 4 [file jem_20252357_sourcedatafs4.pdf]

SF 4A

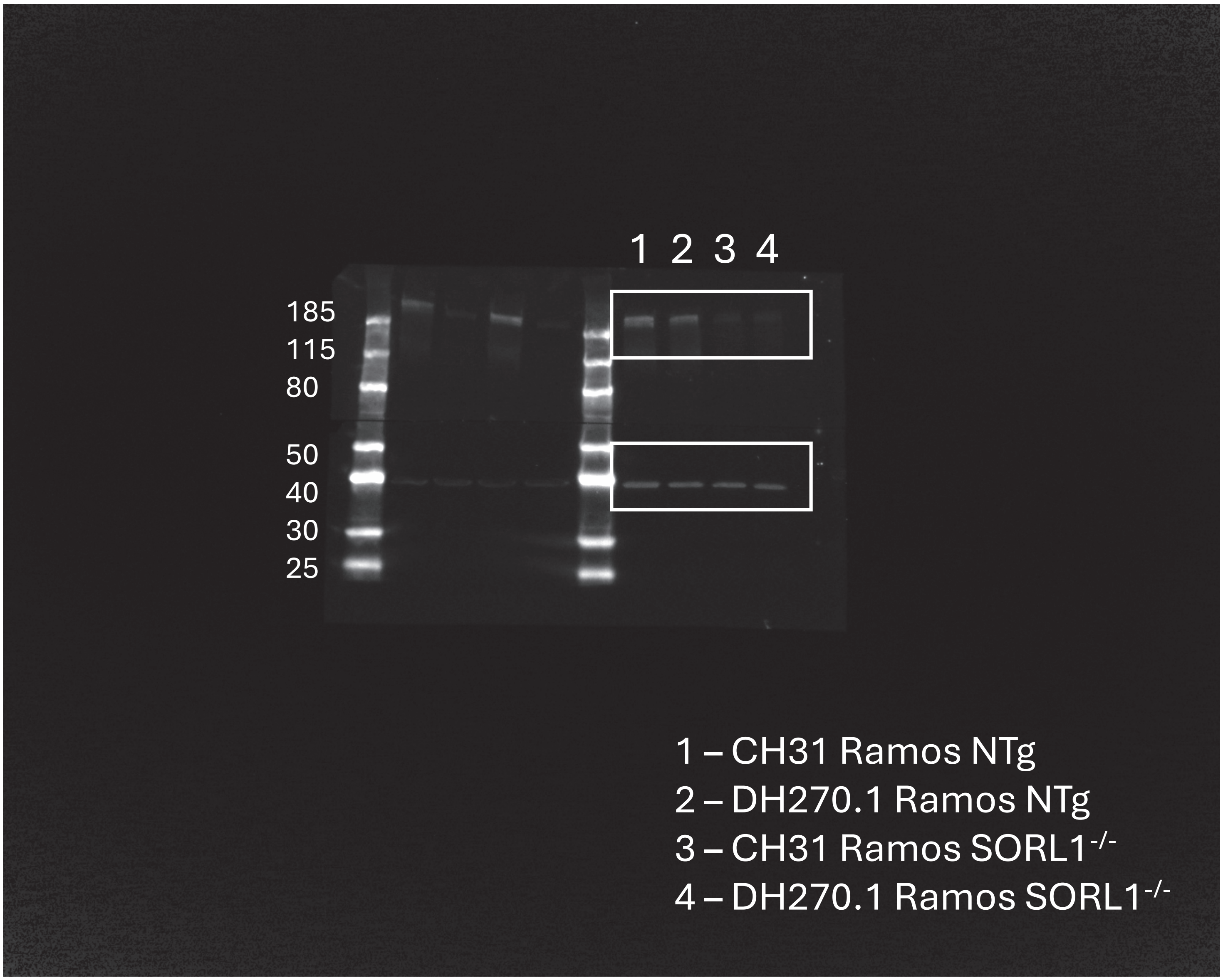
1 2 3 4
185
115
80
50
40
30
25
1 – CH31 Ramos NTg
2 – DH270.1 Ramos NTg
3 – CH31 Ramos SORL1-/-
4 – DH270.1 Ramos SORL1-/-
